# A Facile Approach to Bis(isoxazoles), Promising Ligands of the AMPA Receptor

**DOI:** 10.3390/molecules26216411

**Published:** 2021-10-23

**Authors:** Dmitry A. Vasilenko, Kirill S. Sadovnikov, Kseniya N. Sedenkova, Dmitry S. Karlov, Eugene V. Radchenko, Yuri K. Grishin, Victor B. Rybakov, Tamara S. Kuznetsova, Vladimir L. Zamoyski, Vladimir V. Grigoriev, Vladimir A. Palyulin, Elena B. Averina

**Affiliations:** 1 Department of Chemistry, Lomonosov Moscow State University, 119991 Moscow, Russia; vda-ga@yandex.ru (D.A.V.); 11seconds@mail.ru (K.S.S.); sedenkova@med.chem.msu.ru (K.N.S.); dkar89@gmail.com (D.S.K.); genie@qsar.chem.msu.ru (E.V.R.); grishin@nmr.chem.msu.ru (Y.K.G.); rybakov20021@yandex.ru (V.B.R.); kuzn@med.chem.msu.ru (T.S.K.); vv1950@gmail.com (V.V.G.); 2 Institute of Physiologically Active Compounds, Russian Academy of Sciences, Chernogolovka, 142432 Moscow, Russia; vzam@yandex.ru

**Keywords:** heterocycles, medicinal chemistry, heterocyclization, tetranitromethane, isoxazole, bivalent ligand, AMPA receptor, PAM

## Abstract

A convenient synthetic approach to novel functionalized bis(isoxazoles), the promising bivalent ligands of the AMPA receptor, was elaborated. It was based on the heterocyclization reactions of readily available electrophilic alkenes with the tetranitromethane-triethylamine complex. The structural diversity of the synthesized compounds was demonstrated. In the electrophysiological experiments using the patch clamp technique on Purkinje neurons, the compound 1,4-phenylenedi(methylene)bis(5-aminoisoxazole-3-carboxylate) was shown to be highly potent positive modulator of the AMPA receptor, potentiating kainate-induced currents up to 70% at 10^−11^ M.

## 1. Introduction

An isoxazole ring occurs in various natural and synthetic compounds with a wide spectrum of biological activities [1]. Isoxazole derivatives are known as marketed drugs [2,3,4,5,6] and as promising candidates for the anticancer, antiviral, antifungal, antimicrobial, immunomodulating, analgesic, and antipsychotic drugs, among others [7,8,9,10,11,12,13]. One of the isoxazole-containing compounds, α-amino-3-hydroxy-5-methyl-4-isoxazolepropionic acid (AMPA), is a specific agonist for the AMPA receptor, one of the most important subtypes of glutamate receptors. Involved in many significant neurophysiological processes related to the fast synaptic excitatory transmission, memory formation, etc., the AMPA receptor is a promising pharmacological target [14,15]. Its positive allosteric modulators (PAMs) are potential candidates for the development of cognition enhancers and drugs against the Alzheimer’s and Parkinson’s diseases, multiple sclerosis, soft cognitive disorders, age-related cognition and memory impairments, autism, depression, drug addiction, etc. [16,17,18,19,20,21,22,23,24]. They attract significant interest due to their neuroprotective effect and the lack of the excitotoxicity problems that result from an overdose of a direct agonist. Therefore, the creation of compounds acting as allosteric modulators of AMPA receptors is currently one of the most promising directions for the progress of neuropharmacology.

We were able to develop a series of novel AMPA receptor PAMs based on different scaffolds, which possessed very high levels of experimentally confirmed activity [25,26,27,28,29]. In the design of the compounds for the present study, we took into account that the dimeric and larger monomeric positive allosteric modulators of the AMPA receptor are known to bind more efficiently and demonstrate significantly higher potency in the nanomolar or picomolar concentration ranges [27,28,29,30,31,32], compared with their monomeric units or other small monomeric ligands. This is due to the U-shaped form of the AMPA receptor’s allosteric binding site, which allows both structural fragments in the molecule to interact with the receptor simultaneously. When selecting and prioritizing potential PAM chemotypes for the synthesis and biological evaluation, we considered the pharmacophore and QSAR models [33,34,35] that we have developed, the results of the molecular docking and molecular dynamics simulations, the predicted physico-chemical and ADMET (absorption, distribution, metabolism, excretion, toxicity) profiles, and our previous experience in designing the modulators with some related scaffolds and validating their activity [29,32]. In particular, the structures containing the phenylene-based linkers and aminoisoxazole moieties were identified among the most promising potential PAMs.

Recently, we have elaborated a novel preparative method for the synthesis of 3-EWG-5-nitroisoxazoles, based on the heterocyclization of electrophilic alkenes via treatment with the tetranitromethane-triethylamine (TNM-TEA) complex [36,37,38,39]. Until now, this method has been used to produce polysubstituted isoxazoles containing one heterocyclic fragment in a molecule. In this work, with a focus on the future exploitation of bis(isoxazoles) as bivalent ligands of the AMPA receptor, electrophilic dienes containing two electrophilic double bonds were studied for the first time in the simultaneous heterocyclization of both double bonds under the action of the TNM-TEA complex. We report the synthesis of the desired compounds, as well as the results of their electrophysiological evaluation, the molecular modeling aiming to elucidate the mechanism of action, and the prediction of their physico-chemical and ADMET properties.

## 2. Results and Discussion

### 2.1. Chemistry

Easily available unsaturated esters **2a**-**i** containing two electrophilic double bonds were utilized in the heterocyclization reaction (Table 1).

The starting dienes **2a**-**i** were synthesized from the diols **1a**-**i** using two equivalents of acryl chloride and Et_3_N [40]. Initially, the reaction was performed in CH_2_Cl_2_; however, in the case of the diols **1a**-**f**, the products were obtained in moderate yields due to the low solubility of the starting compounds **1a**-**f**. We found that changing the solvent to CH_3_CN in the acylation reaction significantly improved the yield of **2a**-**f**. For the synthesis of **2g** and **2h**, THF was used due to the low solubility of the corresponding diols **1g** and **1h** in the other solvents. Thus, under the modified conditions, the acrylic esters **2a**-**i** bearing the linear, aromatic, and polycyclic moieties were obtained in good yields.

**Scheme 1:**
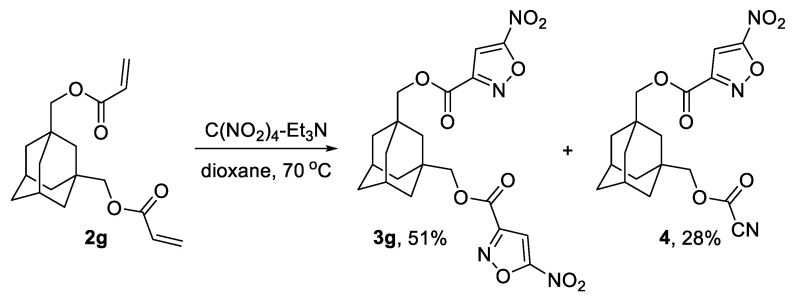
Heterocyclization of the adamantane-containing diene **2g**.

The electrophilic dienes **2a**-**i** were involved in the heterocyclization reaction upon treatment with the in situ generated TNM-TEA complex in 1,4-dioxane at 70 °C to give bis(5-nitroisoxazoles) **3a**-**h** in moderate to good yields. It was found that the yields of the heterocycles **3a**-**c** with the linear linkers tended to increase with the elongation of the carbon chain. The bis(5-nitroisoxazoles) **3d**-**f** bearing the benzyl ester fragments were obtained in modest yields due to the partial oxidation of the benzyl groups in the presence of tetranitromethane, which can act as an oxidizer [41]. It should be noted that the heterocyclization of the adamantane-containing diene **2g** led to the target bis(isoxazole) **3g** also giving a side product **4** (Scheme 1).

**Scheme 2:**
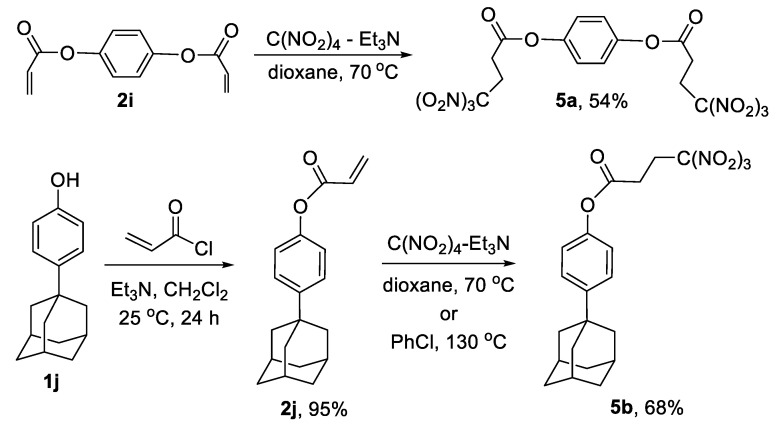
Reaction of the aryl acrylic esters **2i** and **2j** with TNM-TEA.

Surprisingly, we failed to involve the hydroquinone derivative, diene **2i** with isolated double bonds, in the heterocyclization as only bis(4,4,4-trinitrobutanoic ester) **5a** was isolated as the main product (Scheme 2). Trinitro-substituted adduct formation is an alternative pathway for the reaction of electrophilic alkenes with the TNM-TEA complex according to the previously suggested mechanism [36,38]. To affirm whether this route is general for the aryl acrylic esters, we obtained alkene **2j** and employed it in the reaction with TNM-TEA, which also afforded only Michael adduct **5b** under various conditions (Scheme 2).

**Figure 1:**
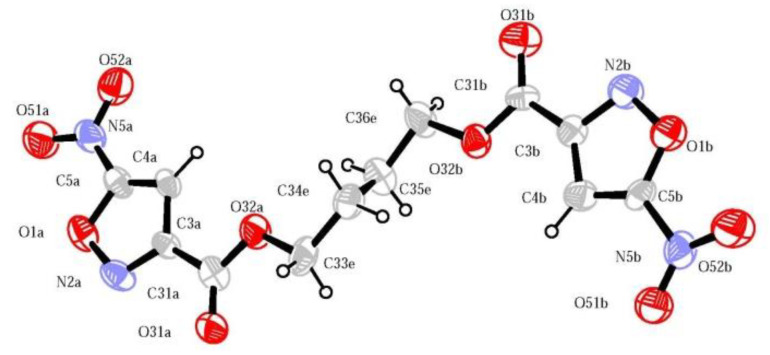
The molecular structure of compound **3c**. Displacement ellipsoids are drawn at the 50% probability level; H atoms are presented as spheres with an arbitrary radius.

All synthesized compounds were characterized and identified using ^1^H and ^13^C NMR spectroscopy (see Supplementary Materials). The structure of the obtained bis(5-nitroisoxazole) **3c** was additionally confirmed by the X-ray analysis (Figure 1) (see Supplementary Materials).

**Scheme 3:**
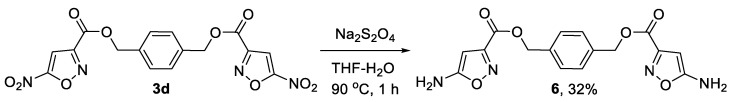
Synthesis of bis(5-aminoisoxazole) **6**.

According to molecular modeling, bis(5-aminoisoxazoles) can be of interest as bivalent modulators of the AMPA receptor and compound **6** is among the most promising ones based on the pharmacophore and QSAR models, the results of the molecular docking and molecular dynamics simulations, and the predicted physico-chemical and ADMET profiles. In view of this, we have studied the reduction of 5-nitro substituted heterocycle **3d** in various conditions [12,13,42,43]. The attempt to reduce 5-nitroisoxazole **3d** using SnCl_2_ in EtOH failed due to its poor solubility, yet the application of the system Na_2_S_2_O_4_/THF/H_2_O allowed us to obtain the target bis(5-aminoisoxazole) **6** in a satisfactory yield (Scheme 3).

**Scheme 4:**
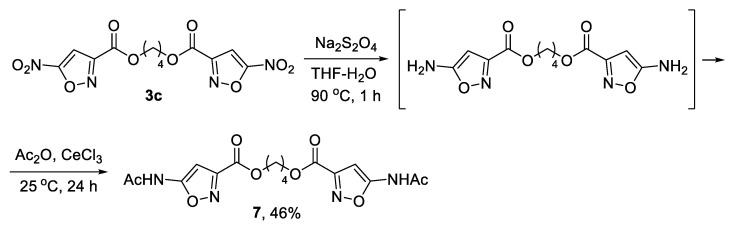
Reduction of bis(5-nitroisoxazole) **3c**.

Unfortunately, the attempts to obtain other bis(5-aminoisoxazoles) failed because of their extremely low solubility in all common solvents. In particular, the application of the different reduction conditions for bis(5-nitroisoxazole) **3c** resulted in a negligibly small amount of corresponding 5-amino derivatives, and only acylated product **7** was successfully isolated in a good yield (Scheme 4). 

### 2.2. Electrophysiological Evaluation

Next, bis(5-aminoisoxazole) **6** was examined as a positive modulator of the AMPA receptor. The electrophysiological experiments were carried out using the patch clamp technique on freshly isolated Purkinje neurons, as described earlier [28,29]. The influence of the compound **6** on the kainate-induced currents is shown in Table 2. The potentiation of the kainate-induced AMPA receptor currents was observed in a wide concentration range (10^–12^–10^–6^ M) and had a bell-shaped concentration dependence with maximum potentiation at 10^–11^ M.

**Figure 2:**
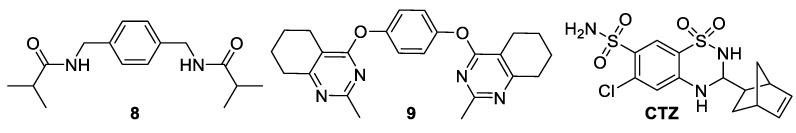
The examples of the known positive modulators of the AMPA receptor.

As can be seen, the activity of the compound **6** was comparable or superior, in terms of effective concentration and/or maximum potentiation, to that of some of the most potent known positive modulators of the AMPA receptor **8**, **9**, and was significantly higher than that of cyclothiazide (**CTZ**) (Figure 2) [29,32,44].

### 2.3. Molecular Modeling

**Figure 3:**
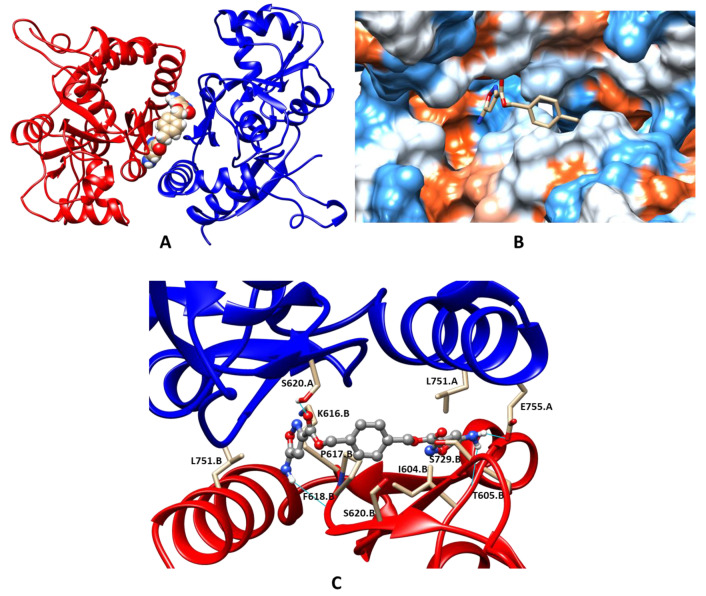
Binding mode of the PAM **6** refined using molecular dynamics simulation. (**A**) General view of the dimeric ligand-binding domain of AMPA receptor (GluA2) and location of the binding site. (**B**) Binding pockets in the protein molecular surface colored by local hydrophobicity (brown for hydrophobic and blue for hydrophilic). (**C**) Detailed view of the binding site. The ligand is represented by a grey ball-and-stick model, the amino acid residues located within 3 Å of it are represented by beige stick models. Hydrogen bonds are shown as cyan lines.

**Figure 4:**
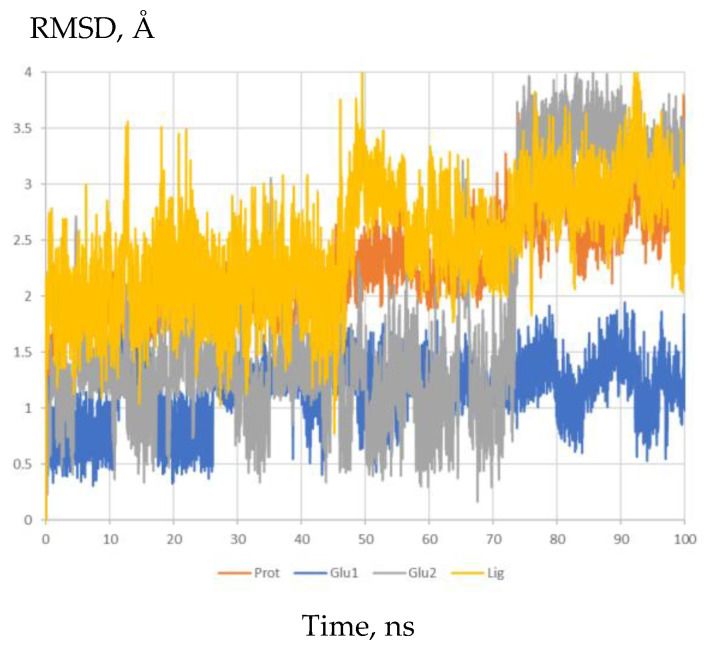
RMSD of the protein, glutamate, and ligand (**6**) heavy atoms during molecular dynamics simulation of the PAM complex with the dimeric ligand-binding domain of the GluA2 AMPA receptor.

In order to elucidate the probable mechanism of action of the positive allosteric modulator **6**, its interaction with the dimeric ligand-binding domain of the GluA2 AMPA receptor was modeled using a molecular docking and molecular dynamics simulation. The compound’s binding mode in the PAM binding site, at the interface between ligand-binding domains, was stable over the entire course of the simulation (100 ns). Similarly to the other larger dimeric modulators [28], the **6** molecule attained a slightly unsymmetric position, occupying a central subpocket as well as one of the side subpockets of the symmetrical PAM binding site (Figure 3A,B). The binding was stabilized by steric fit, hydrophobic interactions, and a number of hydrogen bonds (Figure 3B,C). The plot of the root mean square deviations (RMSD) for the protein, glutamate, and ligand (**6**) heavy atoms (Figure 4) as well as the visual inspection of the trajectory confirm that the system stability was retained over the entire course of the production simulation (100 ns), although the ligand position was slightly adjusted compared to the docking pose. Overall, these results confirmed that the compound **6** could indeed act as a positive AMPA receptor modulator binding in the validated PAM binding site.

### 2.4. Prediction of ADMET, Physicochemical, and PAINS Profiles

Several ADMET and physicochemical properties for the compound **6** were calculated (Table 3). They demonstrated a high predicted value for the intestinal absorption, enabling its oral administration. The predicted lipophilicities and aqueous solubilities were also appropriate for potential drug-like compounds according to the commonly accepted rules of thumb. Due to the moderate predicted blood–brain barrier permeability (brain concentration is expected to be about 10% of the plasma concentration), acceptable CNS bioavailability could be anticipated. Both parameters of the cardiac toxicity risk (hERG p*K_i_* and pIC_50_) (4.1–6.0 log units) were in the lower or medium parts of their possible ranges (3–9 log units), indicating a likely absence of the hERG liabilities. The integral quantitative estimate of drug-likeness (QED) was greater than 0.6, confirming the favorable likely properties. The pan assay interference compounds (PAINS) filter check did not identify any alerts. 

Overall, the predicted ADMET, physicochemical, and PAINS properties of the positive allosteric modulator **6** were quite acceptable for the potential lead compounds at the early drug development stages, although additional checks and structure optimization would likely be required.

## 3. Materials and Methods

### 3.1. Chemistry

The ^1^H and ^13^C NMR spectra were recorded on the 400 MHz spectrometers Bruker Avance 400 and Agilent 400-MR (400.0 MHz for ^1^H; 100.6 MHz for ^13^C) at room temperature. The chemical shifts δ were measured in ppm with respect to the solvent (^1^H: CDCl_3_, δ = 7.26 ppm, CD_3_OD, δ = 3.31 ppm, CD_3_C(O)CD_3_, δ = 2.05 ppm, (CD_3_)_2_SO, δ = 2.50 ppm; ^13^C: CDCl_3_, δ = 77.16 ppm, CD_3_OD, δ = 49.0 ppm, CD_3_C(O)CD_3_, δ = 29.84 ppm, (CD_3_)_2_SO δ = 39.52 ppm). Chemical shifts (δ) are given in ppm; *J* values are given in Hz. When necessary, the assignments of signals in the NMR spectra were made using 2D techniques. The accurate mass measurements (HRMS) were performed on a Bruker micrOTOF II instrument, using electrospray ionization (ESI). The measurements were performed in a positive ion mode (interface capillary voltage 4500 V), or in a negative ion mode (3200 V). The melting points (mp) were uncorrected. The analytical thin layer chromatography was carried out with Silufol silica gel plates (supported on aluminum), and the detection was performed by a UV lamp (254 and 365 nm) and chemical staining (5% aqueous solution of KMnO_4_). The column chromatography was performed on silica gel (230–400 mesh, Merck, Darmstadt, Germany). 

Diols **1d** [45], **1e** [46], **1f** [47], **1g** [48] were synthesized by the described methods. Dienes **2a-f,i** were also synthesized by the described method [40].

All other starting materials were commercially available.

#### 3.1.1. General Procedure for the Preparation of Compounds **2g,h**

Triethylamine (1.40 mL, 1.01 g, 10.0 mmol) was added to the solution of starting diol **1** (5.0 mmol) in THF (70 mL) under an Ar atmosphere. The mixture was cooled to (–10)–(–5) °C and the acryloyl chloride (0.81 mL, 0.91 g, 10.0 mmol) was added dropwise. The resulting mixture was stirred at this temperature for 3 h, and then at room temperature for 24 h. After cooling, the reaction mixture was filtered through a layer of Al_2_O_3,_ which was washed with CH_2_Cl_2_. The filtrate was concentrated in vacuo, and the obtained residue was purified by silica-gel column chromatography to afford the desired product.

*Adamantane*-*1,3*-*diylbis(methylene) diacrylate (**2g**)* was isolated to a pure state as a colorless oil with a 46% (0.70 g) yield; R_f_ 0.28 (petroleum ether:EtOAc = 20:1); ^1^H NMR (CDCl_3_, 400 MHz): δ 1.32–1.38 (m, 2H, CH_2_), 1.42–1.56 (m, 8H, 4CH_2_), 1.58–1.66 (m, 2H, CH_2_), 2.04–2.12 (m, 2H, 2CH), 3.78 (s, 4H, 2CH_2_O), 5.80 (dd, ^2^*J* = 1.6 Hz, ^3^*J* = 10.6 Hz, 2H, 2CH_2_), 6.11 (dd, ^3^*J* = 10.6 Hz, ^3^*J* = 17.4 Hz, 2H, 2CHCO), 6.35 (dd, ^2^*J* = 1.6 Hz, ^3^*J* = 17.4 Hz, 2H, 2CH_2_); ^13^C NMR (CDCl_3_, 100.6 MHz): δ 28.0 (2CH), 33.8 (CH_2_), 36.3 (CH_2_), 38.8 (4CH_2_), 41.1 (2C), 73.6 (2CH_2_O), 128.6 (2CH=), 130.6 (2CH=), 166.3 (C=O); HRMS-ESI (M + Na^+^): Found: 327.1555. Calculated for C_18_H_24_NaO_4_^+^: 327.1567.

*[(3s,7s)*-*7*-*(Acryloyloxy)bicyclo[3.3.1]non*-*3*-*yl]methyl acrylate (**2h**)* was isolated to a pure state as colorless oil with a 54% (0.64 g) yield; R_f_ 0.49 (petroleum ether:EtOAc = 10:1); ^1^H NMR (CDCl_3_, 400 MHz): δ 1.10–1.20 (m, 1H, CH_2_), 1.37– 1.54(m, 2H, CH_2_), 1.69–1.81 (m, 2H, CH_2_), 1.82–1.92 (m, 2H, CH_2_), 1.90–2.10 (m, 4H, 2CH_2_), 2.10–2.20 (m, 2H, 2CH), 3.94 (d, ^3^*J*= 6.6 Hz, 2H, CH_2_O), 5.16–5.26 (m, 1H, CHO), 5.80 (dd, ^2^*J* = 1.5 Hz, ^3^*J* = 10.5 Hz, 1H, CH_2_), 5.82 (dd, ^2^*J* = 1.5 Hz, ^3^*J* = 10.5 Hz, 1H, CH_2_), 6.10 (dd, ^3^*J* = 10.5 Hz, ^3^*J* = 17.3 Hz, 2H, 2CH), 6.38 (dd, ^2^*J* = 1.5 Hz, ^3^*J* = 17.3 Hz, 2H, CH_2_); ^13^C NMR (CDCl_3_, 100.6 MHz): δ 23.5 (2CH), 28.1 (CH_2_), 29.4 (2CH_2_), 29.7 (CH), 37.3 (2CH_2_), 70.3 (CHO + CH_2_O), 128.7 (=CH), 129.4 (=CH), 130.6 (=CH_2_), 130.6 (=CH_2_), 165.6 (C=O), 166.4 (C=O); HRMS-ESI (M + Na^+^): Found: 301.1412. Calculated for C_16_H_22_NaO_4_^+^: 301.1410.

#### 3.1.2. Synthesis of Compound **2j**

Triethylamine (0.70 mL, 0.51 g, 5.0 mmol) was added to the solution of **1j** (1.14 g, 5.0 mmol) in CH_2_Cl_2_ (10.00 mL) under an Ar atmosphere. The mixture was cooled to (–10)–(–5) °C and the acryloyl chloride (0.40 mL, 0.46 g, 5.0 mmol) was added dropwise. The resulting mixture was stirred at this temperature for 3 h, and then at room temperature for 24 h. The reaction mixture was poured into water (15 mL) and extracted with dichloromethane (4 × 20 mL). The combined organic layer was washed with brine solution (3 × 20 mL) and was dried over anhydrous Mg_2_SO_4_. The solvent was evaporated in vacuo, and the residue was purified by flash chromatography on silica gel to afford the desired product.

*4*-*(Adamantan*-*1*-*yl)phenyl acrylate (**2j**)* was isolated to a pure state as a colorless solid with a 95% (1.34 g) yield; mp. = 98–99 °C; ^1^H NMR (CDCl_3_, 400 MHz): δ 1.71–1.84 (m, 6H, 3CH_2_(Ad)), 1.88–1.97 (m, 6H, 3CH_2_(Ad)), 2.07–2.15 (m, 3H, 3CH(Ad)), 5.99 (dd, *^2^J* = 1.2 Hz, *^3^J* = 10.6 Hz, 1H, CH_2_), 6.32 (dd, *^3^J* = 10.6 Hz, *^3^J* = 17.6 Гц, 1H, CHCO), 6.60 (dd, *^2^J* = 1.2 Hz, *^3^J* = 17.6 Hz, 1H, CH_2_), 7.05–7.11 (m, 2H, 2CH(Ar)), 7.35–7.41 (m, 2H, 2CH(Ar)). ^13^C NMR (CDCl_3_, 100.6 MHz): δ 29.0 (3CH), 36.1 (C) 36.8 (3CH_2_), 43.3 (3CH_2_), 120.9 (2CH(Ar)), 126.0 (2CH(Ar)), 128.2 (=CH), 132.4 (=CH_2_), 148.3 (C), 149.0 (C), 164.8 (C=O); HRMS-ESI (M + H^+^): Found: 283.1691. Calculated for C_19_H_23_O_2_^+^: 283.1693.

#### 3.1.3. General Procedure for the Preparation of Bis(isoxazoles) **3a-h**, **4**, **5a**

Triethylamine (0.56 mL, 0.40 g, 4.0 mmol) was added dropwise to a solution of tetranitromethane (0.60 mL, 0.98 g, 5.0 mmol) in 1,4-dioxane (6.00 mL) at 0 °C (ice bath). The reaction mixture was stirred for 5 min, and alkene (1.0 mmol) in 2.00 mL of 1,4-dioxane was added dropwise. Then, the cooling factor was removed and the mixture was stirred at 70 °C for 2 h. The solvent was removed under reduced pressure, and the product was isolated by column chromatography.

*Ethane*-*1,2*-*diyl bis(5*-*nitroisoxazole*-*3*-*carboxylate) (**3a**)* was isolated to a pure state as a colorless solid with a 46% (0.16 g) yield; mp.= 138–141 °C; R_f_ 0.24 (petroleum ether:EtOAc = 3:1); ^1^H NMR (CDCl_3_, 400 MHz): δ 4.81 (s, 4H, 2CH_2_O), 7.42 (s, 2H, 2CH); ^13^C NMR (CDCl_3_, 100.6 MHz): δ 64.0 (2CH_2_O), 102.4 (2CH), 157.6 (2C), 157.7 (2C), 166.0 (2CNO_2_); Anal. Calculated for C_6_H_6_N_2_O_5_: C, 35.10; H, 1.77; N, 16.37. Found: C, 35.12; H, 1.79; N, 16.09.

*Propane*-*1,3*-*diyl bis(5*-*nitroisoxazole*-*3*-*carboxylate) (**3b**)* was isolated to a pure state as a colorless solid with a 55% (0.20 g) yield; mp. = 75–77 °C; R_f_ 0.14 (petroleum ether:EtOAc = 4:1). ^1^H NMR (CDCl_3_, 400 MHz): δ 2.35 (quint., ^3^*J* = 6.1 Hz, 2H, CH_2_), 4.59 (t, ^3^*J* = 6.1 Hz, 4H, 2CH_2_O), 7.43 (s, 2H, 2CH); ^13^C NMR (CDCl_3_, 100.6 MHz): δ 27.7 (CH_2_), 63.7 (2CH_2_O), 102.5 (2CH), 157.7 (2C), 158.0 (2C), 165.8 (2CNO_2_); Anal. Calculated for C_6_H_6_N_2_O_5_: C, 37.09; H, 2.26; N, 15.73. Found: C, 37.57; H, 2.07 N, 15.21.

*Butane*-*1,4*-*diyl bis(5*-*nitroisoxazole*-*3*-*carboxylate) (**3c**)* was isolated to a pure state as a colorless solid with a 64% (0.24 g) yield; mp. = 124 °C; R_f_ 0.16 (petroleum ether:EtOAc = 4:1). ^1^H NMR (CDCl_3_, 400 MHz): δ 1.95–2.03 (m, 4H, 2CH_2_), 4.48–4.56 (m, 4H, 2CH_2_O), 7.41 (s, 2H, 2CH); ^13^C NMR (CDCl_3_, 100.6 MHz): δ 25.2 (2CH_2_), 66.5 (2CH_2_O), 102.4 (2CH), 157.8 (2C=O), 158.2 (2C), 165.9 (2CNO_2_).

*1,4*-*Phenylenedi(methylene) bis(5*-*nitroisoxazole*-*3*-*carboxylate) (**3d**)* was isolated to a pure state as a colorless solid with a 32% (0.13 g) yield; mp. = 150–151 °C; R_f_ 0.24 (petroleum ether:EtOAc = 4:1); ^1^H NMR (CDCl_3_, 400 MHz): δ 5.46 (s, 4H, 2CH_2_O), 7.40 (s, 2H, 2CH), 7.50 (s, 4H, 4CH(Ar)); ^13^C NMR (CDCl_3_, 100.6 MHz): δ 68.3 (2CH_2_O), 102.5 (2CH), 129.4 (4CH(Ar)), 135.1 (2C(Ar)), 157.6 (2C=O), 158.1 (2C), 165.8 (2CNO_2_); HRMS-ESI (M + Na^+^): Found: 441.0296. Calculated for C_16_H_10_N_4_NaO_10_^+^: 441.0289.

*1,3*-*Phenylenedi(methylene) bis(5*-*nitroisoxazole*-*3*-*carboxylate)****(3e)*** was isolated to a pure state as a colorless solid with a 40% (0.17 g) yield; mp. = 108–110 °C; R_f_ 0.22 (petroleum ether:EtOAc = 3:1). ^1^H NMR (CDCl_3_, 400 MHz): δ 5.47 (s, 4H, 2CH_2_O), 7.41 (s, 2H, 2CH), 7.45–7.50 (m, 3H, 3CH(Ar)), 7.53–7.56 (m, 1H, CH(Ar)); ^13^C NMR (CDCl_3_, 100.6 MHz): δ 68.3 (2CH_2_O), 102.5 (2CH), 129.1 (CH(Ar)), 129.52 (2CH(Ar)), 129.59 (CH(Ar)), 134.9 (2C(Ar)), 157.6 (2C=O), 158.1 (2C), 165.8 (2CNO_2_); HRMS-ESI (M + Na^+^): Found: 441.0283. Calculated for C_16_H_10_N_4_NaO_10_^+^: 441.0289.

*1,2*-*Phenylenedi(methylene) bis(5*-*nitroisoxazole*-*3*-*carboxylate) (**3f**)* was isolated to a pure state as a colorless solid with a 22% (92 mg) yield; mp. = 112–114 °C; R_f_ 0.56 (petroleum ether:EtOAc = 3:1); ^1^H NMR (CDCl_3_, 400 MHz): δ 5.62 (s, 4H, 2CH_2_O), 7.40 (s, 2H, 2CH), 7.43–7.49 (m, 2H, 2CH(Ar)), 7.52–7.59 (m, 2H, 2CH(Ar)); ^13^C NMR (CDCl_3_, 100.6 MHz): δ 66.3 (2CH_2_O), 102.5 (2CH), 120.1 (2CH(Ar)), 131.3 (2CH(Ar)), 133.3 (2C(Ar)), 157.5 (2C=O), 158.0 (2C), 165.8 (2CNO_2_); HRMS-ESI (M + Na^+^): Found: 441.0285. Calculated for C_16_H_10_N_4_NaO_10_^+^: 441.0289.

*Adamantane*-*1,3*-*diylbis(methylene) bis(5*-*nitroisoxazole*-*3*-*carboxylate) (**3g**)* was isolated to a pure state as a colorless solid with a 51% (0.24 g) yield; mp. = 127–129 °C; Rf 0.27 (petroleum ether:EtOAc = 5:1); ^1^H NMR (CDCl_3_, 400 MHz): δ 1.46–1.53 (m, 2H, CH_2_(Ad)), 1.54–1.67 (m, 8H, 4CH_2_(Ad)), 1.67–1.73 (m, 2H, CH_2_(Ad)), 2.13–2.24 (m, 2H, 2CH(Ad)), 4.09 (m, 4H, 2CH_2_O), 7.39 (s, 2H, 2CH); ^13^C NMR (CDCl_3_, 100.6 MHz): δ 27.7 (2CH(Ad)), 34.1 (2C(Ad)), 36.0 (CH_2_(Ad)), 38.5 (4CH_2_(Ad)), 40.7 (CH_2_(Ad)), 75.7 (2CH_2_O), 102.4 (2CH), 157.9 (2C=O), 158.3 (2C), 165.8 (2CNO_2_); HRMS-ESI (M + Na^+^): Found: 499.1065. Calculated for C_20_H_20_N_4_NaO_10_^+^: 499.1072.

*(3*-*(((Cyanocarbonyl)oxy)methyl)adamantan*-*1*-*yl)methyl 5*-*nitroisoxazole*-*3*-*carboxylate (**4**)* was isolated to a pure state as a colorless solid with a 28% (0.11 g) yield; mp. = 67–68 °C; Rf 0.47 (petroleum ether:EtOAc = 5:1); ^1^H NMR (CDCl_3_, 400 MHz): δ 1.41–1.45 (m, 2H, CH_2_), 1.47–1.72 (m, 10H, 5CH_2_), 2.15–2.22 (m, 2H, CH), 3.98 (s, 2H, CH_2_O), 4.09 (s, 2H, CH_2_O), 7.40 (s, 1H, CH); ^13^C NMR (CDCl_3_, 100.6 MHz): δ 27.6 (2CH), 33.9 (C), 34.0 (C), 35.9 (CH_2_), 38.2 (2CH_2_), 38.4 (2CH_2_), 40.5 (CH_2_), 75.6 (CH_2_O), 77.4 (CH_2_O), 102.5 (CH), 109.4 (C), 144.5 (C), 157.9 (C), 158.2 (C); HRMS-ESI (M + K^+^): Found: 428.0850. Calculated for C_18_H_19_N_3_KO_7_^+^: 428.0855.

*((3s,7s)*-*7*-*{[(5*-*nitroisoxazol*-*3*-*yl)carbonyl]oxy}bicyclo[3.3.1]non*-*3*-*yl)methyl 5*-*nitroisoxazole*-*3*-*carboxylate* (***3h***) was isolated to a pure state as a light yellow oil with a 65% (0.29 g) yield; R_f_ 0.21 (petroleum ether:EtOAc = 7:1); ^1^H NMR (CDCl_3_, 400 MHz): δ 1.19–1.27 (m, 1H, CH_2_), 1.53–1.63 (m, 2H, 2CH_2_), 1.82–1.91 (m, 2H, 2CH_2_), 1.91–1.97 (m, 1H, CH_2_), 1.97–2.13 (m, 4H, 4CH_2_), 2.15–2.29 (m, 3H, 3CH), 4.35 (d, ^3^*J*= 7.1 Hz, 2H, CH_2_O), 5.45–5.54 (m, 1H, CHO), 7.39 (s, H, CH), 7.44 (s, H, CH); ^13^C NMR (CDCl_3_, 100.6 MHz): δ 23.1 (2CH), 27.7 (CH_2_), 28.9 (2CH_2_), 29.6 (CH), 37.2 (2CH_2_), 72.4 (CH_2_O), 74.0 (CHO), 102.50 (CH), 102.54 (CH), 157.2 (C=O), 157.7 (C=O), 158.5 (C), 158.8 (C), 165.7 (2CNO_2_); HRMS-ESI (M + K^+^): Found: 489.0660. Calculated for C_18_H_18_KN_4_O_10_^+^: 489.0655.

*1,4*-*Phenylene bis(4,4,4*-*trinitrobutanoate) (**5a**)* was isolated to a pure state as a colorless solid with a 54% (0.28 g) yield; mp. = 150–151 °C; Rf 0.53 (petroleum ether:EtOAc = 5:1); ^1^H NMR (CD_3_C(O)CD_3_, 400 MHz): δ 3.24 (t, ^3^*J* = 7.5 Hz, 2H, CH_2_), 3.87 (br.t, ^3^*J* = 7.5 Hz, 2H, CH_2_), 7.22 (c, 4H, 4CH); ^13^C NMR (CD_3_C(O)CD_3_, 100.6 MHz): δ 29.1 (2CH_2_), 29.9 (2CH_2_), 123.4 (4CH(Ar)), 131.1 (2C(NO_2_)_3_), 149.2 (2C(Ar)), 169.5 (2C=O); HRMS-ESI (M + NH_4_^+^): Found: 538.0643. Calculated for C_14_H_16_N_7_O_16_^+^: 538.0648.

#### 3.1.4. Synthesis of Compound **5b**

Triethylamine (0.28 mL, 0.20 g, 2.0 mmol) was added dropwise to a solution of tetranitromethane (0.30 mL, 0.49 g, 2.5 mmol) in 1,4-dioxane (4.00 mL) at 0 °C (ice bath). The reaction mixture was stirred for 5 min, and alkene **2j** (282 mg, 1.0 mmol) in 2.00 mL of 1,4-dioxane was added dropwise. Then, the cooling factor was removed, and the mixture was stirred at 70 °C for 2 h. The solvent was removed under reduced pressure, and the product was isolated by column chromatography.

*4*-*(Adamantan*-*1*-*yl)phenyl 4,4,4*-*trinitrobutanoate (**5b**)* was isolated to a pure state as a colorless solid with a 68% (0.59 g) yield; mp. = 125–127 °C; R_f_ 0.72 (petroleum ether:EtOAc = 10:1); ^1^H NMR (CDCl_3_, 400 MHz): δ 1.70–1.84 (m, 6H, 3CH_2_(Ad)), 1.86–1.95 (m, 6H, 3CH_2_(Ad)), 2.06–2.15 (m, 3H, 3CH(Ad)), 3.03 (t, *^3^J* = 7.5 Hz, 2H, CH_2_), 3.50 (br. t, *^3^J* = 7.5 Hz, 2H, CH_2_), 7.00–7.06 (m, 2H, 2CH(Ar)), 7.35–7.41 (m, 2H, 2CH(Ar)). ^13^C NMR (CDCl_3_, 100.6 MHz): δ 28.7 (CH_2_), 29.0 (3CH(Ad)), 29.8 (CH_2_), 36.1 (C(Ad)), 36.8 (3CH_2_(Ad)), 43.3 (3CH_2_(Ad)), 120.6 (2CH(Ar)), 126.2 (2CH(Ar)), 128.9 (C(NO_2_)_3_), 148.0 (C), 149.7 (C), 168.3 (C=O); HRMS-ESI (M + NH_4_^+^): Found: 451.1828. Calculated for C_20_H_27_N_4_O_4_^+^: 451.1823.

#### 3.1.5. Synthesis of Compound **6**

Na_2_S_2_O_4_ (1.05 g, 6.0 mmol) was added to a solution of 1,4-phenylenedi(methylene) bis(5-nitroisoxazole-3-carboxylate) (418 mg, 1.0 mmol) in a mixture of THF (8.00 mL) and H_2_O (8.00 mL). The resulting mixture was stirred at 90 °C for 1 h and then cooled down to room temperature. Then, H_2_O (8.00 mL) and HCl conc. (3.70 mL) were added. The resulting mixture was stirred at 60 °C for 15 min, and then cooled down to room temperature and quenched with NaHCO_3_. The mixture was poured into brine (20 mL) and extracted with EtOAc (4 × 20 mL). The combined organic layers were washed with brine (20 mL) and dried (anhyd. MgSO_4_). The solvent was evaporated in vacuo, and the title product was isolated by column chromatography.

*1,4*-*Phenylenedi(methylene) bis(5*-*aminoisoxazole*-*3*-*carboxylate)****(6)*** was isolated to a pure state as a colorless solid with a 32% (0.12 g) yield; mp. = 150–151 °C; R_f_ 0.24 (petroleum ether:EtOAc = 4:1); ^1^H NMR (CD_3_OD, 400 MHz): δ 5.35 (c, 4H, 2CH_2_O), 5.38 (c, 2H, 2CH), 7.47 (c, 4H, 4CH(Ar)). ^13^C NMR (CD_3_OD, 100.6 MHz): δ 67.8 (2CH_2_O), 79.2 (2CH), 129.7 (4CH(Ar)), 137.2 (2C(Ar)), 158.1 (2C=O), 161.7 (2C), 173.8 (2C); HRMS-ESI (M + Na^+^): Found: 381.0802. Calculated for C_16_H_14_N_4_NaO_6_^+^: 381.0806.

#### 3.1.6. Synthesis of Compound **7**

Na_2_S_2_O_4_ (1.05 g, 6.0 mmol) was added to a solution of butane-1,4-diyl bis(5-nitroisoxazole-3-carboxylate) (0.37 mg, 1.0 mmol) in a mixture of THF (8.00 mL) and H_2_O (8.00 mL). The resulting mixture was stirred at 90 ^o^C for 1 h and then cooled down to room temperature. Then, the resulting mixture was concentrated in vacuo. The residue was suspended in Ac_2_O (5.00 mL, 5.40 g, 53.0 mmol), and cerium (III) chloride (493 mg, 2.0 mmol) was added. The reaction mixture was stirred for 24 h, and then poured into water and neutralized with a solution of NaHCO_3_ (up to pH 7–8). The product was extracted by CH_2_Cl_2_ (4 × 20 mL). The combined organic extract was dried over MgSO_4_. The solvent was removed under reduced pressure and the product was isolated by column chromatography.

*Butane*-*1,4*-*diyl bis(5*-*acetamidoisoxazole*-*3*-*carboxylate) (**7**)* was isolated to a pure state as a colorless solid with a 46% (0.18 g) yield; mp. = 225–226 ^o^C with decomposition; R_f_ 0.34 (CH_2_Cl_2_:MeOH = 20:1). ^1^H NMR (DMSO, 400 MHz): δ 1.77–1.85 (m, 2H, 2CH_2_), 2.12 (s, 6H, 2CH_3_), 4.30–4.41 (m, 2H, 2CH_2_O), 6.47 (c, 2H, 2CH), 11.94 (c, 2H, 2NH). ^13^C NMR (CDCl_3_-CD_3_OD, 100.6 MHz): δ 23.0 (2CH_3_), 25.1 (2CH_2_), 65.4 (2CH_2_O), 89.0 (2CH), 157.0 (2C), 159.9 (2C=O), 162.9 (2C), 167.6 (2C=O); HRMS-ESI (M + H^+^): Found: 395.1192. Calculated for C_16_H_19_N_4_O_8_^+^: 395.1197.

### 3.2. Electrophysiological Evaluation

In vitro electrophysiological experiments were carried out using a patch clamp technique with the local fixation of potential, as described earlier [28,29]. Freshly isolated single Purkinje neurons from the cerebellum of 12–15-day old Wistar rats were used as a test system. Transmembrane currents were induced by the activation of the AMPA receptors with a solution of their partial agonist kainic acid, using a fast superfusion of solutions, where 30 μL of the agonist buffer (the agonist concentration was varied in the range of 10^–6^–10^–4^ M) were added to the constant flow of the neuron-washing buffer. The applications for the control and for each concentration of a compound were performed in triplicate. The transmembrane currents for the individual neurons were recorded using 2.5–5.5 MΩ borosilicate microelectrodes in a whole-cell configuration with an EPC-9 device from HEKA, Germany. The data were processed by the Pulsfit program from HEKA, Germany. Cyclothiazide (CTZ), as a well-known positive allosteric modulator of AMPA receptors, was used as a reference ligand. The experimental results for compound **6** are presented in Table 2.

### 3.3. Molecular Modeling

The structure of the dimeric ligand-binding domain of the GluA2 AMPA receptor was obtained from the Protein Data Bank (PDB: 4FAT) [49]. Upon the removal of the ions and small molecules (except for the two receptor-bound glutamate agonist molecules), the protein was allowed to relax during the molecular dynamics simulation for 100 ns (see below for the simulation protocol). The most frequently occurring structure was identified by the clustering of the frames in the stable part of the trajectory (40–100 ns). The ligand structure was converted to 3D and preoptimized in the MMFF94 force field using Avogadro 1.2.0 software [50], and then the ligand and protein structures were prepared for molecular docking using AutoDock Tools 1.5.6 [51]. The molecular docking to the positive allosteric modulator binding site was performed with AutoDock Vina 1.1.2 software [52] (grid box size 22 Å × 29 Å × 40 Å, exhaustiveness = 16). The pose with the best scoring function value and ligand position was selected, and the complex model was built using the USCF Chimera 1.15 software [53].

The molecular dynamics simulations were performed using the CHARMM36/CGenFF 4.4 force field [54,55] in the GROMACS 2021.2 software [56]. The initial models of the systems were built using the Ligand Reader & Modeler and Solution Builder modules of the CHARMM-GUI web service [57,58]. The protein molecule was inserted into a rectangular box of water in the TIP3P model; the distance from the protein to the box border was no less than 10 Å. Individual, randomly selected water molecules were replaced with potassium and chlorine ions to ensure the electrical neutrality of the system and the total concentration of KCl about 0.15 M. For each system, the molecular mechanics minimization (up to 5000 steps) was performed on the CPU, followed by equilibration for 125 ps at the temperature of 300 K and a constant volume using the v-rescale thermostat on the NVIDIA GeForce RTX 3080 GPU. The production simulation was performed on the GPU at the constant pressure of 1 atm and the temperature of 300 K using the v-rescale thermostat and the Parrinello–Rahman barostat. The hydrogen atom movements were constrained using the LINCS algorithm. For the analysis and visualization of the results, the CPPTRAJ software [59] in the AmberTools 21 package [60] and UCSF Chimera were used.

### 3.4. Prediction of ADMET, Physicochemical, and PAINS Profiles

The lipophilicity (LogPow) and aqueous solubility (pSaq) were estimated by the ALogPS 3.0 neural network model implemented in the OCHEM platform [61]. Human intestinal absorption (HIA) [62], blood–brain barrier permeability (LogBB) [63,64], and hERG-mediated cardiac toxicity risk (channel affinity p*K_i_* and inhibitory activity pIC_50_) [65] were estimated using the integrated online service for ADMET properties prediction (ADMET Prediction Service) [66]. This server implements predictive QSAR models based on accurate and representative training sets, fragmental descriptors, and artificial neural networks. The quantitative estimate of drug-likeness (QED) values [67] were calculated and the pan assay interference compounds (PAINS) alerts were checked using RDKit version 2020.03.4 software [68].

## 4. Conclusions

In conclusion, we have developed an efficient, simple, and concise approach to the previously unknown functionalized bis(isoxazoles), based on the heterocyclization of readily available electrophilic dienes upon treatment with a TNM-TEA complex. The reaction tolerates a variety of linkers in bis(heterocycles) including aliphatic, aromatic, and polycyclic moieties. The remarkable activity of bis(isoxazoles) as positive allosteric modulators of the AMPA receptor was demonstrated by the example of bis(5-aminoisoxazole) **6**, which caused a potentiation of the kainate-induced AMPA receptor currents in a wide concentration range (10^–12^–10^–6^ M). The molecular modeling confirmed that this compound could interact with the validated PAM binding site. Its predicted ADMET, physicochemical, and PAINS properties were quite acceptable for potential lead compounds at the early drug development stages. These results allow us to expect further beneficial applications of this structural type of heterocycles in the search for new compounds with nootropic and neuroprotective properties.

## Figures and Tables

**Table 1:**
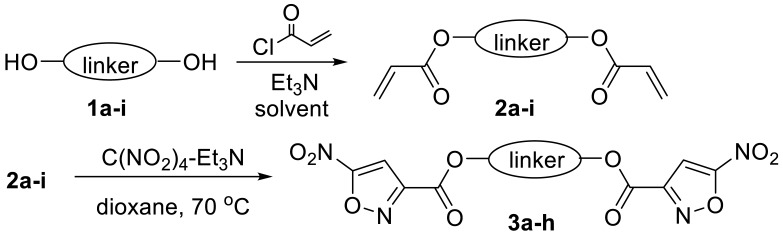
Synthesis of electrophilic dienes **2** and bis(5-nitroisoxazoles) **3**.

Entry	Compound	Linker	Solvent ^1^	Yield, % ^2^	
				2	3
1	**a**	–(CH_2_)_2_–	MeCN	73	46
2	**b**	–(CH_2_)_3_–	MeCN	76	55
3	**c**	–(CH_2_)_4_–	MeCN	81	64
4	**d**	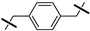	MeCN	85	32
5	**e**	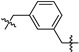	MeCN	84	40
6	**f**	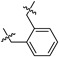	MeCN	82	22
7	**g**	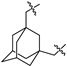	THF	46	51
8	**h**	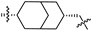	THF	54	65
9	**i**	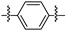	CH_2_Cl_2_	99	–

^1^ For the acylation of diols **1a**-**i**; ^2^ Isolated yields.

**Table 2:** The effect of various concentrations of compounds on the kainate-induced AMPA receptor currents in rat cerebellum Purkinje cells.

Compound	Number of Neurons	Compound Concentration/M, Current Amplitude (% to Control, ±SD)						
		10^–1^^2^	10^–1^^1^	10^–1^^0^	10^–^^9^	10^–^^8^	10^–^^7^	10^–^^6^
**6**	4	141 ± 7	172 ± 9	152 ± 7	144 ± 5	129 ± 4	113 ± 4	105 ± 3
**8** [29]	5		106 ± 6	118 ± 7	140 ± 8	128 ± 5	119 ± 4	119 ± 3
**9** [32]	7	108 ± 5	132 ± 5	143 ± 9	170 ± 11	123 ± 8	85 ± 6	78 ± 4
**CTZ**	8						100 ± 3	145 ± 11

**Table 3:** Predicted physicochemical and ADMET profiles of compound **6**.

Compound	MW	LogPow	pSaq	LogBB	HIA	hERG p*K_i_*	hERG pIC_50_	QED
**6**	358.31	1.92	3.22	−0.98	99	5.98	4.13	0.62

Note: MW—molecular weight, LogPow—octanol-water partition coefficient, pSaq—aqueous solubility [–log(M)], LogBB—blood–brain barrier permeability, HIA—human intestinal absorption [%], hERG *pK_i_*—hERG potassium channel affinity [–log(M)], hERG pIC_50_—hERG potassium channel inhibitory activity [–log(M)], QED—quantitative estimate of drug-likeness.

## Data Availability

Data of the compounds are available from the authors.

## Supplementary Materials

Copies of the NMR spectra and the X-ray data are available online.

## Sample Availability

Samples of the compounds are available from the authors.
