# Supplementary material for: A Facile Approach to Bis(isoxazoles), Promising Ligands of the AMPA Receptor
Source: Molecules. 2021 Oct 23;26(21):6411. doi: 10.3390/molecules26216411 (PMC8588558; doi:10.3390/molecules26216411)
Supplement: Supplementary file 1 [file molecules-26-06411-s001.zip › molecules-1413658-supplementary.pdf]

Facile approach to bis(isoxazoles) – promising ligands of AMPA receptor

Dmitry A. Vasilenko,[a] Kirill S. Sadovnikov,[a] Kseniya N. Sedenkova,[a] Dmitry S. Karlov,[a] Eugene V. Radchenko,[a] Yuri K. Grishin,[a] Victor B. Rybakov,[a] Tamara S. Kuznetsova,[a] Vladimir L. Zamoyski,[b] Vladimir V. Grigoriev,[a,b] Vladimir A. Palyulin,[a] Elena B. Averina*[a]

[a] *Department of Chemistry, Lomonosov Moscow State University, 119991 Moscow, Russian Federation. E-mail: elaver@med.chem.msu.ru*

[b] *Institute of Physiologically Active Compounds, Russian Academy of Sciences, 142432 Chernogolovka, Moscow Region, Russian Federation*

SUPPORTING INFORMATION

TABLE OF CONTENTS

1. Copies of NMR Spectra……………………………………………………….......… 2

2. X-Ray Data for Compound **3c**.. 43

1.Copies of NMR Spectra

Adamantane-1,3-diyldi(methylene) bisacrylate **2g** (^{1}H NMR)

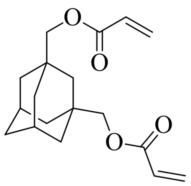

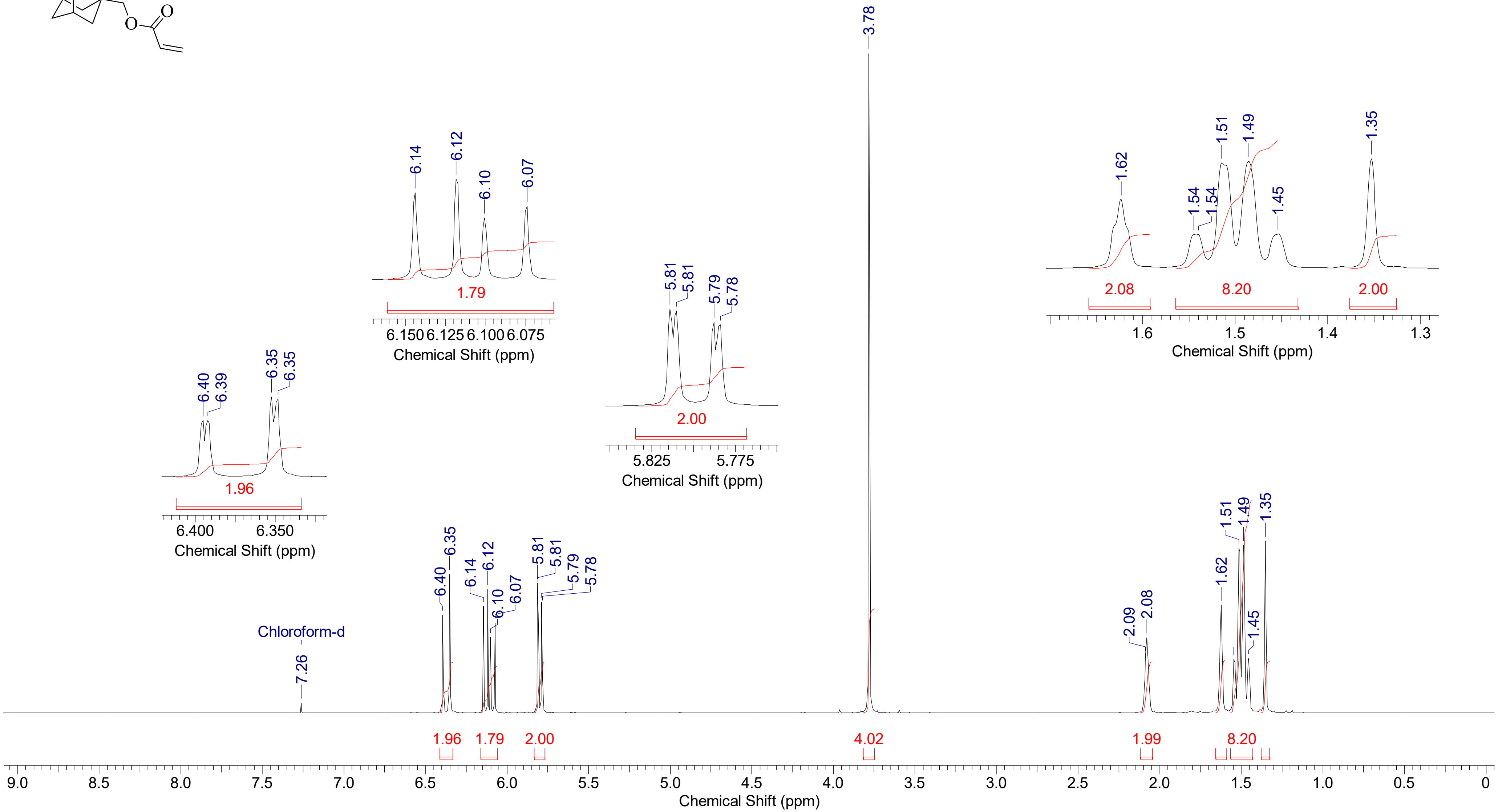

Adamantane-1,3-diyldi(methylene) bisacrylate **2g** (^{13}C NMR)

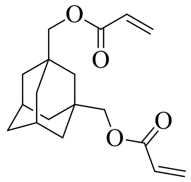

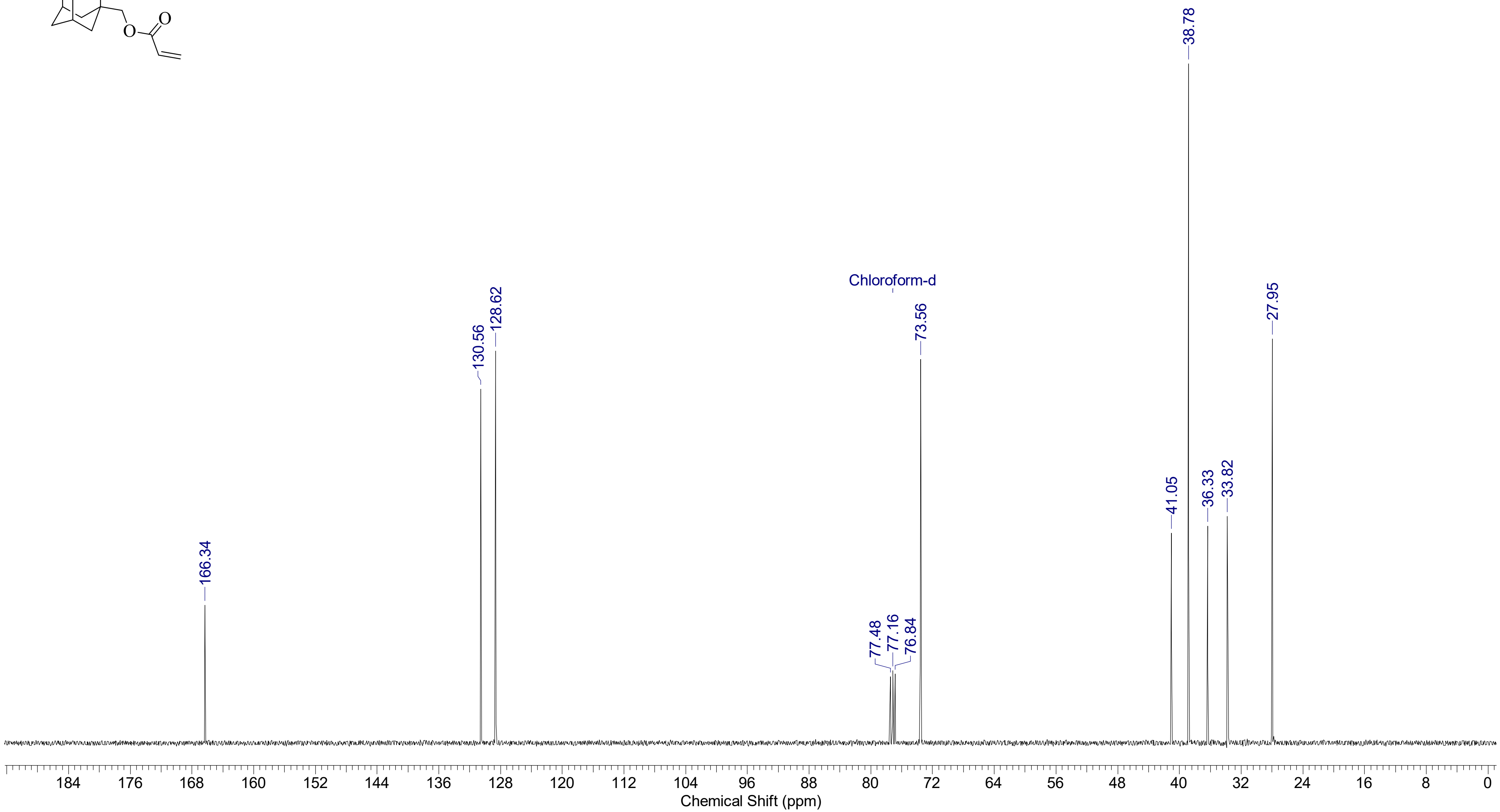

[(3s,7s)-7-(Acryloyloxy)bicyclo[3.3.1]non-3-yl]methyl acrylate **2h** (^{1}H NMR)

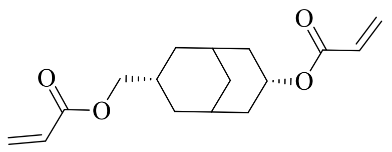

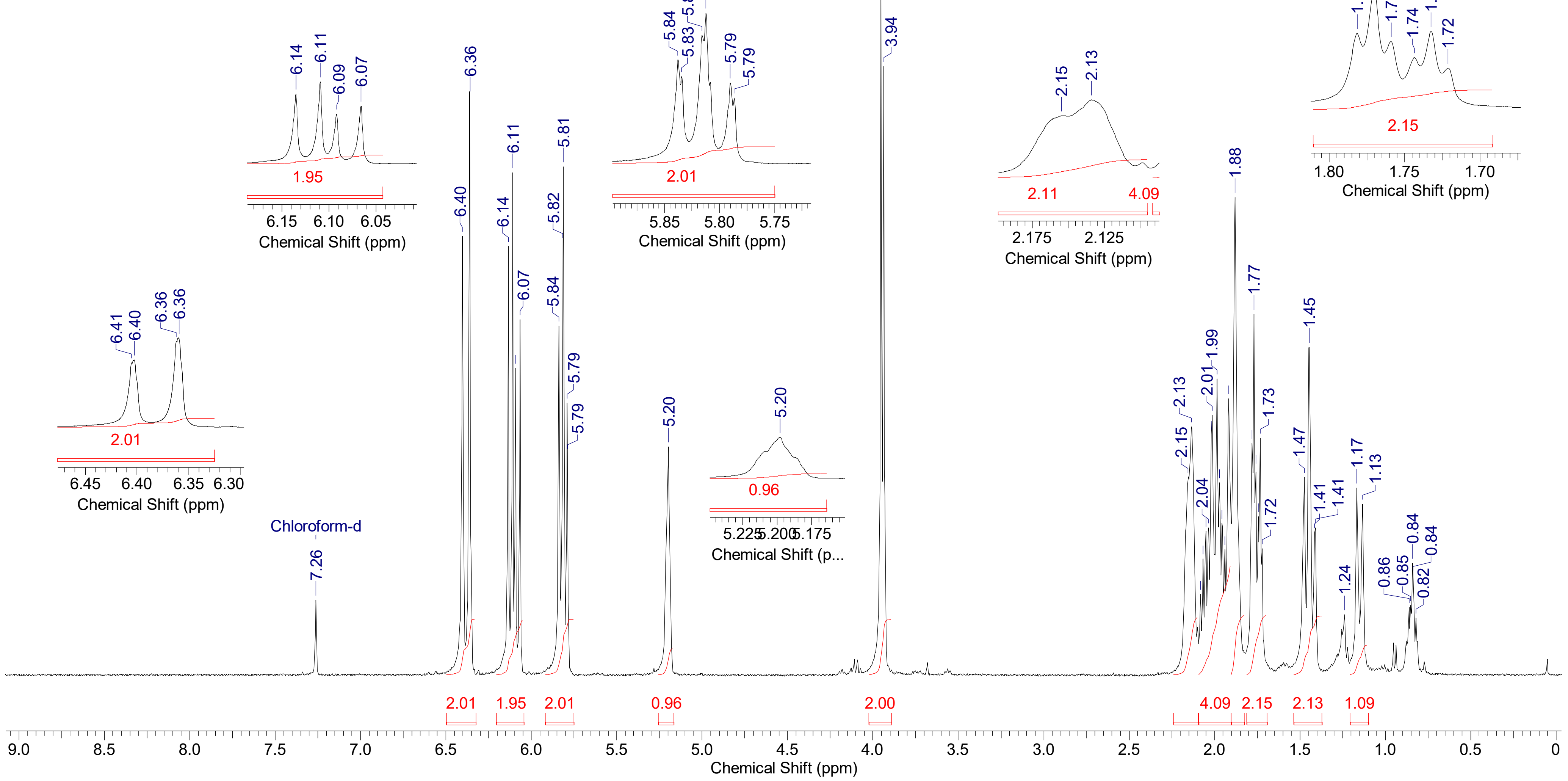

[(3s,7s)-7-(Acryloyloxy)bicyclo[3.3.1]non-3-yl]methyl acrylate **2h** (^{13}C NMR)

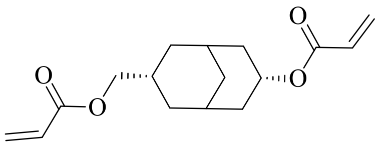

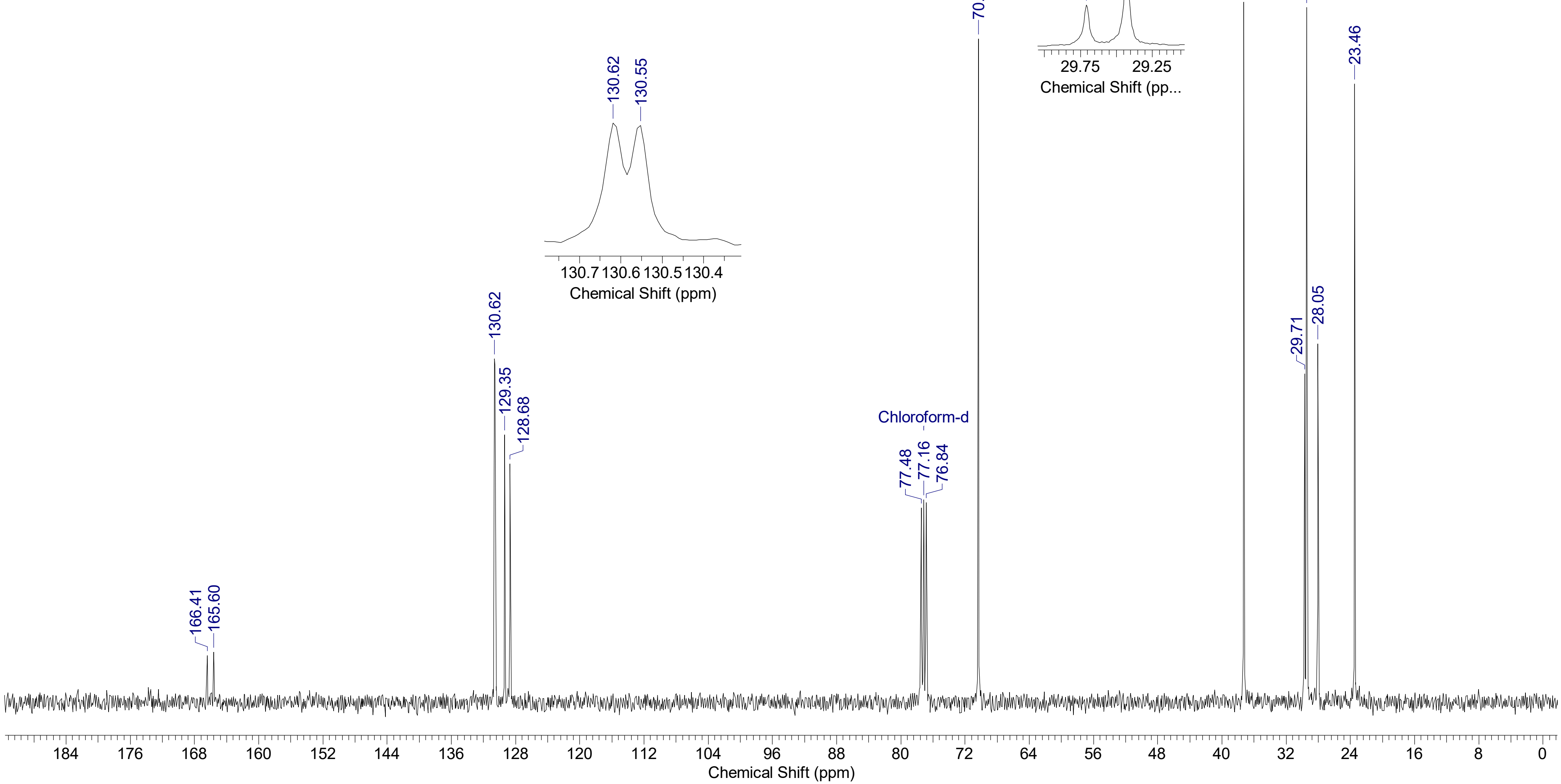

4-(Adamantan-1-yl)phenyl acrylate, **2j** (^{1}H NMR)

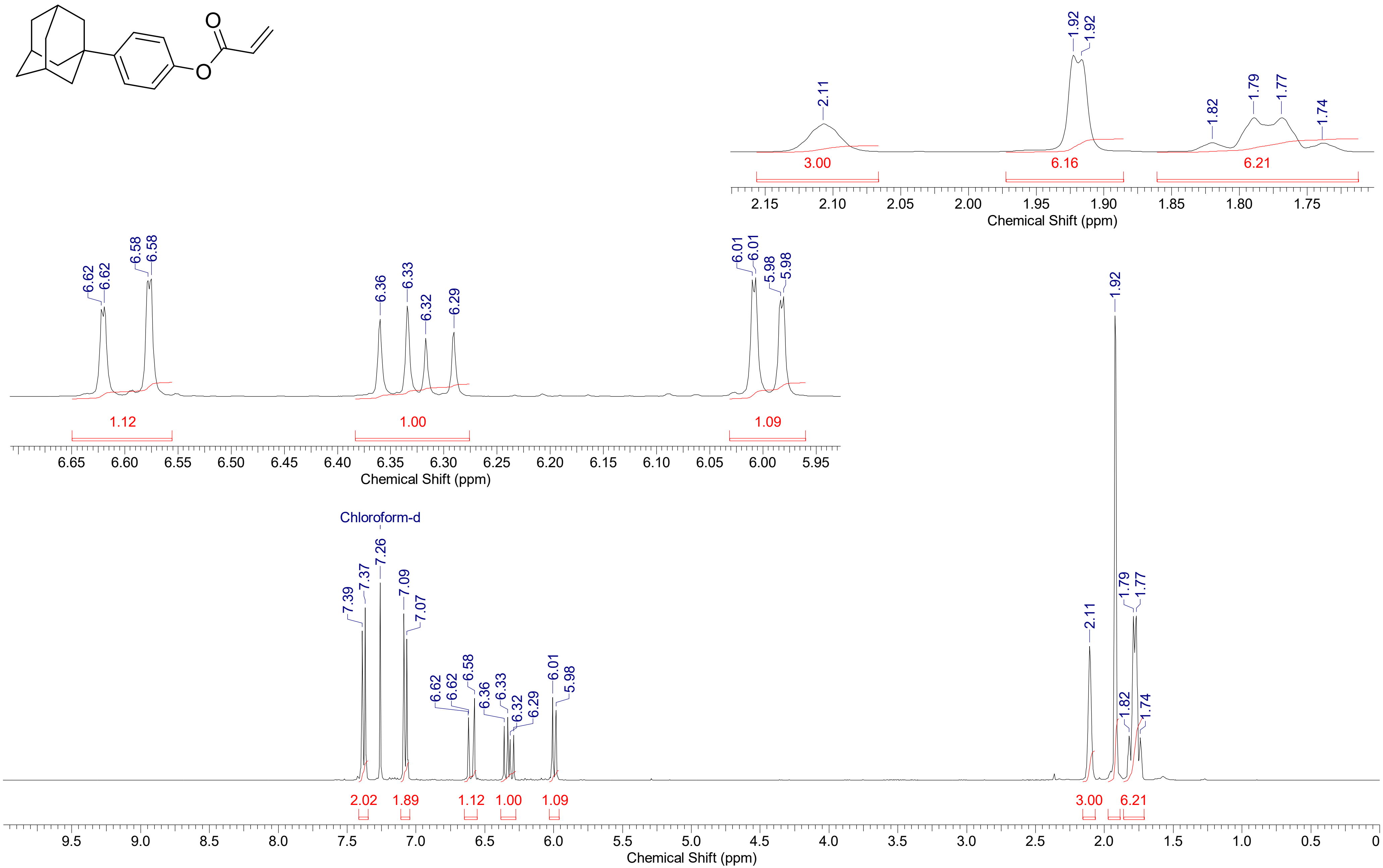

4-(Adamantan-1-yl)phenyl acrylate, **2j** (^{13}C NMR)

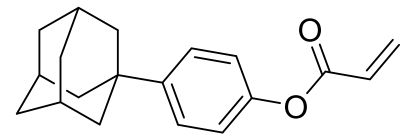

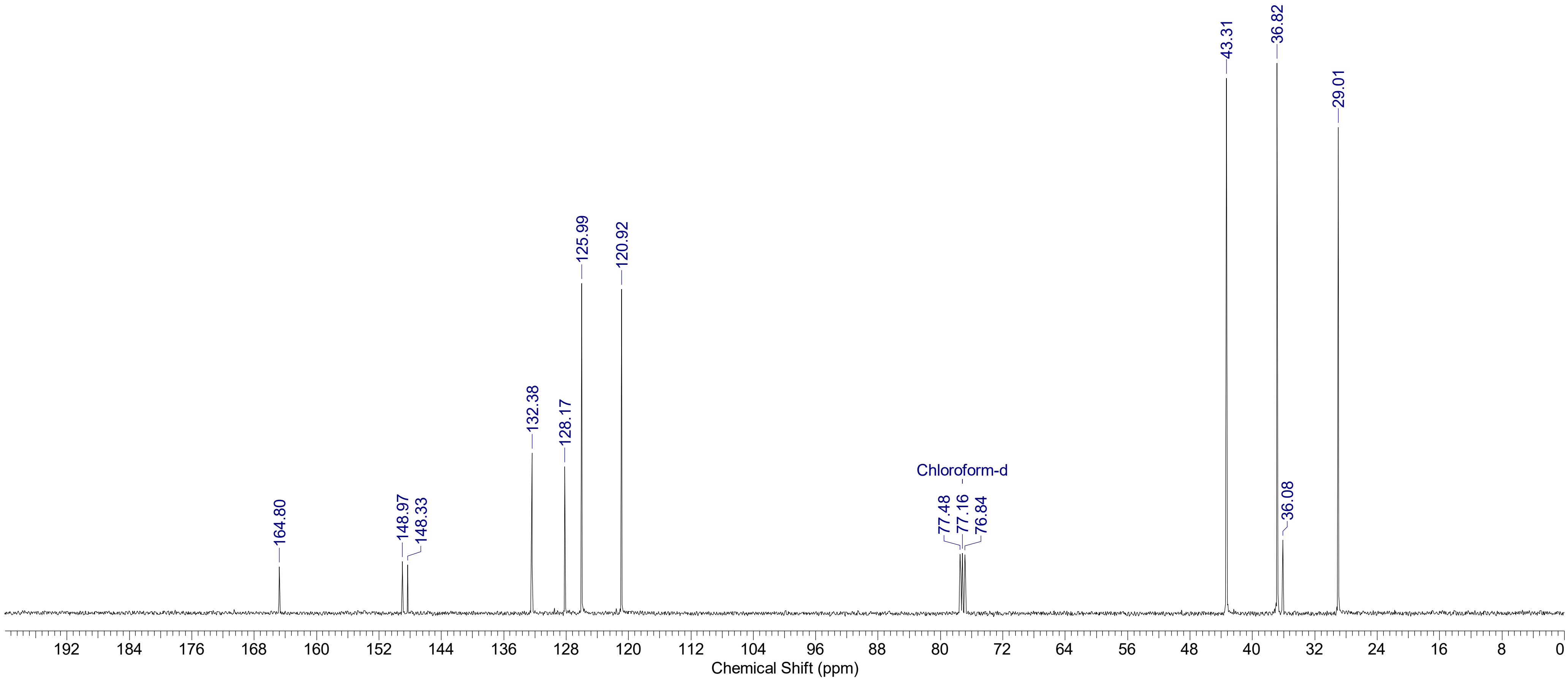

Ethane-1,2-diyl bis(5-nitroisoxazole-3-carboxylate) **3a** (^{1}H NMR)

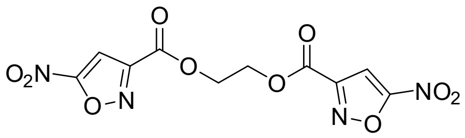

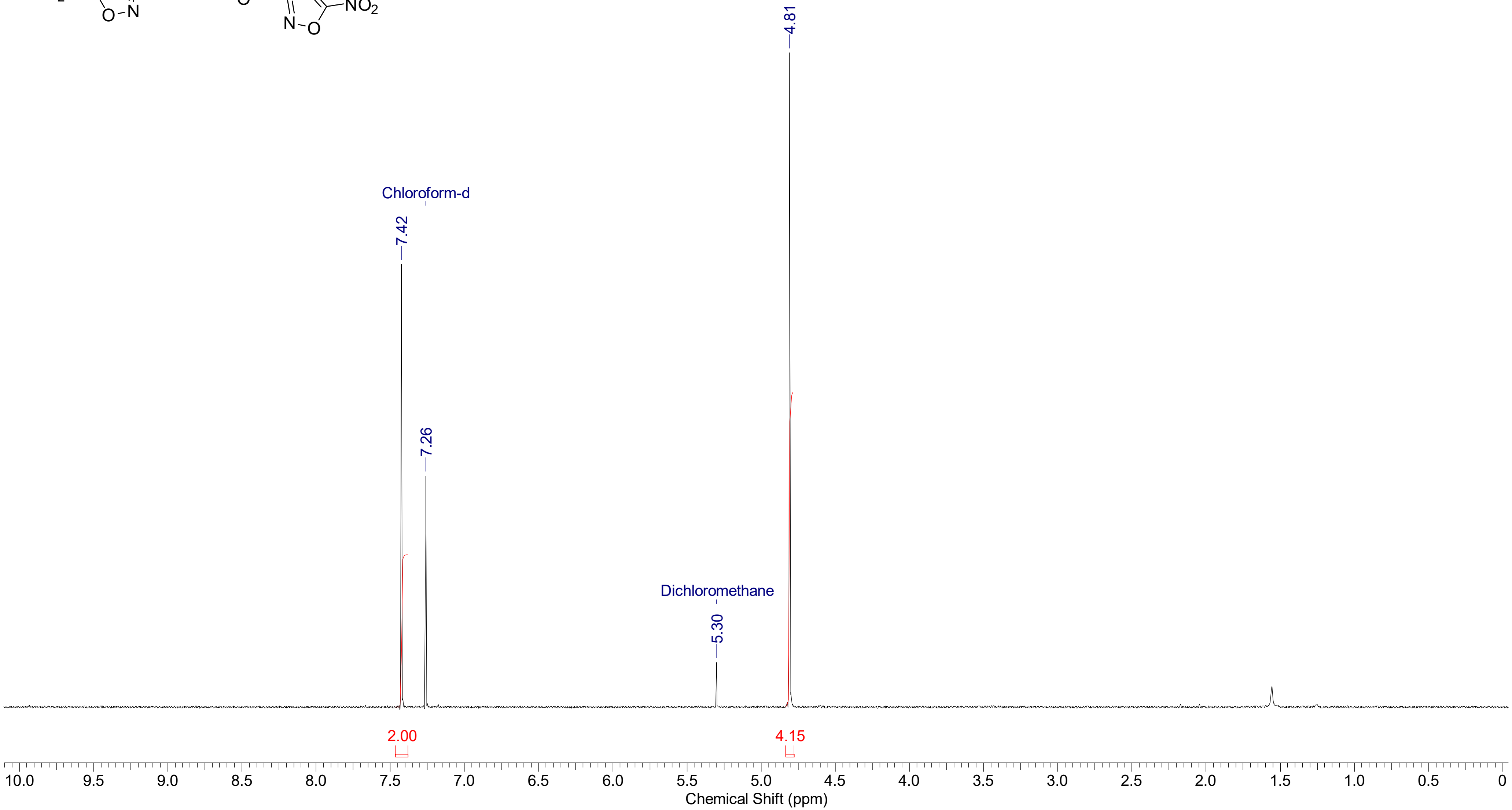

Ethane-1,2-diyl bis(5-nitroisoxazole-3-carboxylate) **3a** (^{13}C NMR)

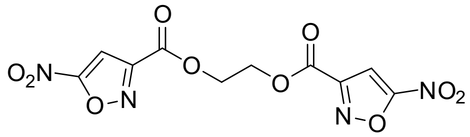

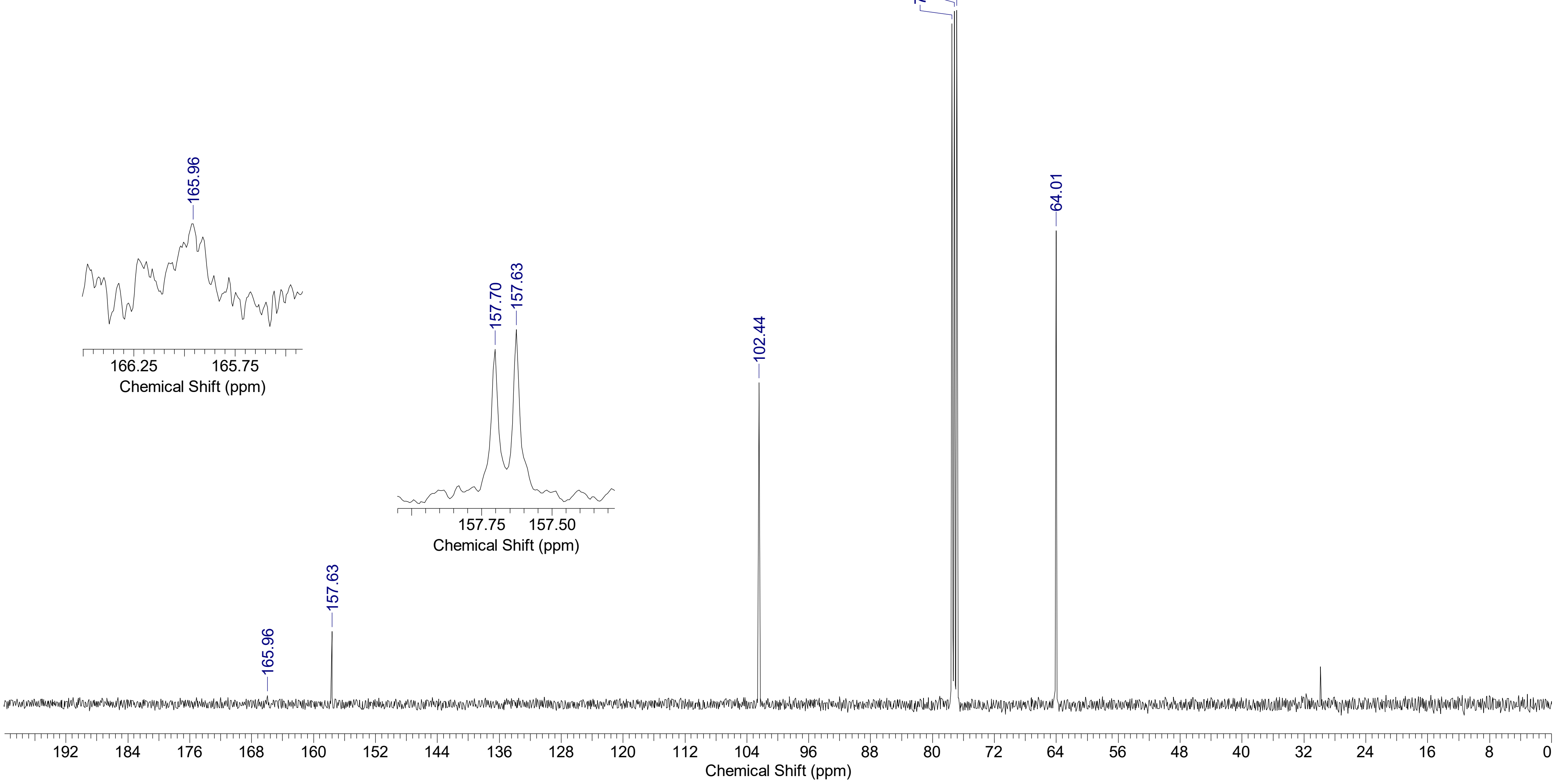

Propane-1,3-diyl bis(5-nitroisoxazole-3-carboxylate) **3b** (^{1}H NMR)

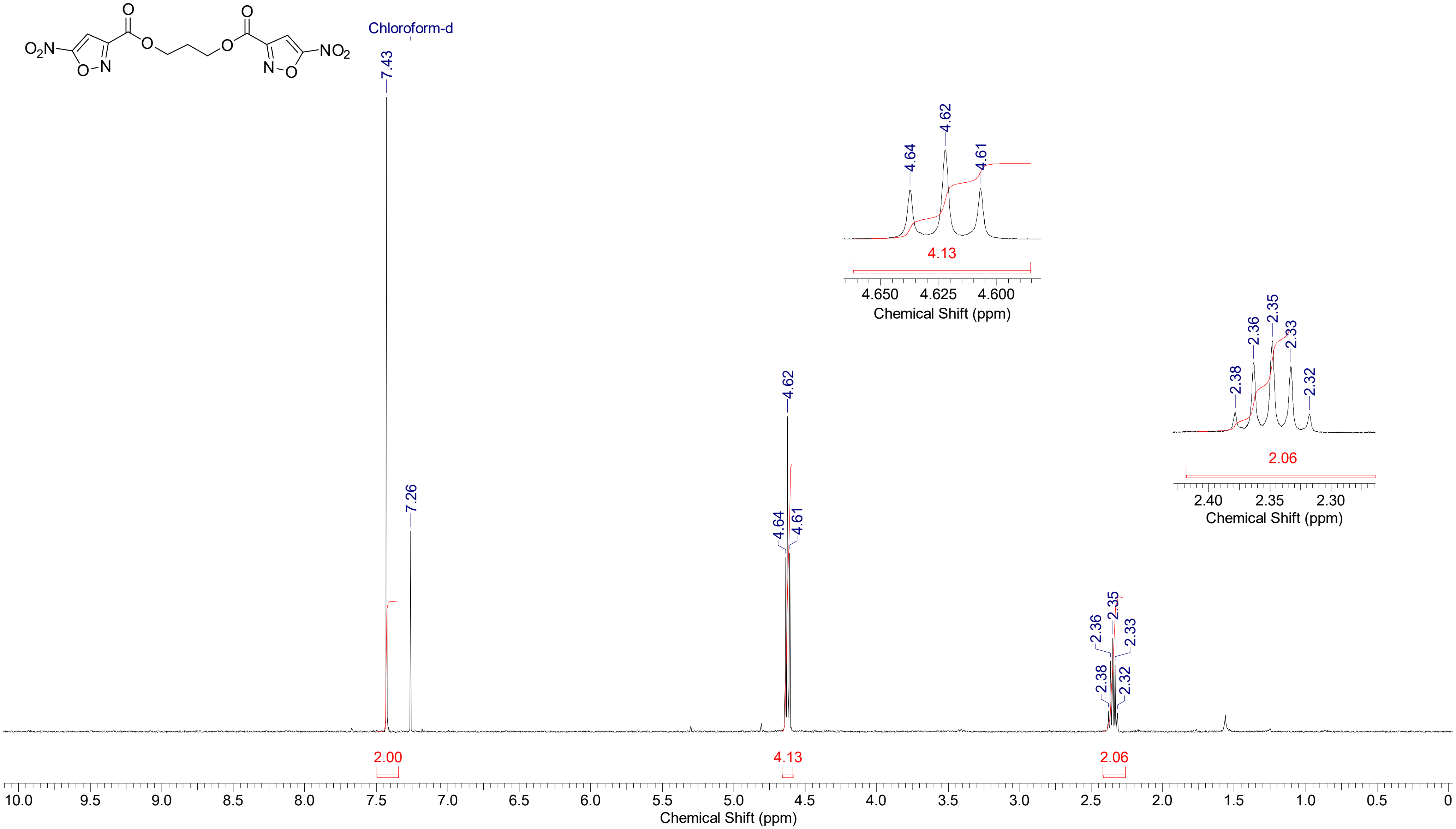

Propane-1,3-diyl bis(5-nitroisoxazole-3-carboxylate) **3b** (^{13}C NMR)

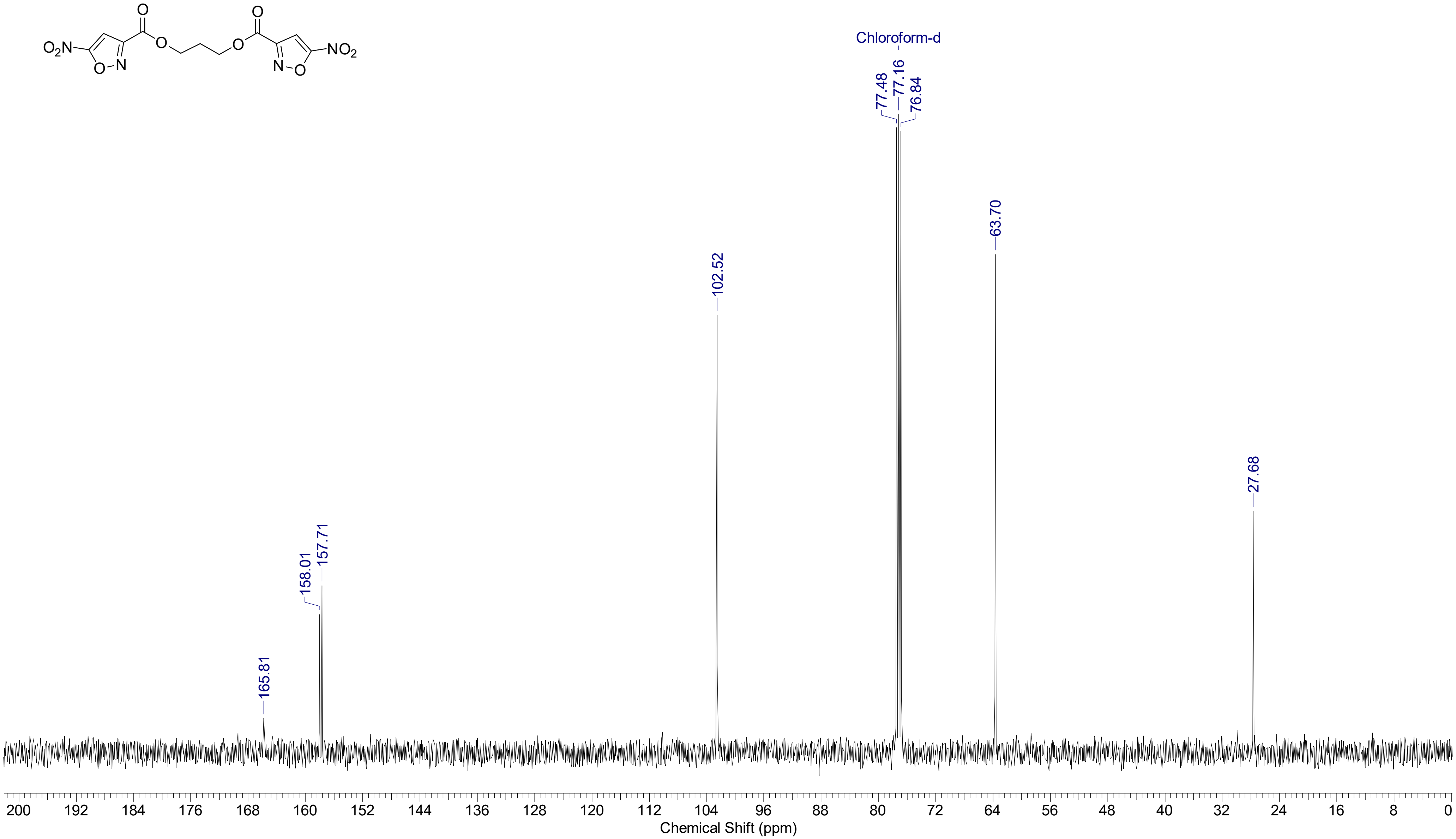

Butane-1,4-diyl bis(5-nitroisoxazole-3-carboxylate) **3c** (^{1}H NMR)

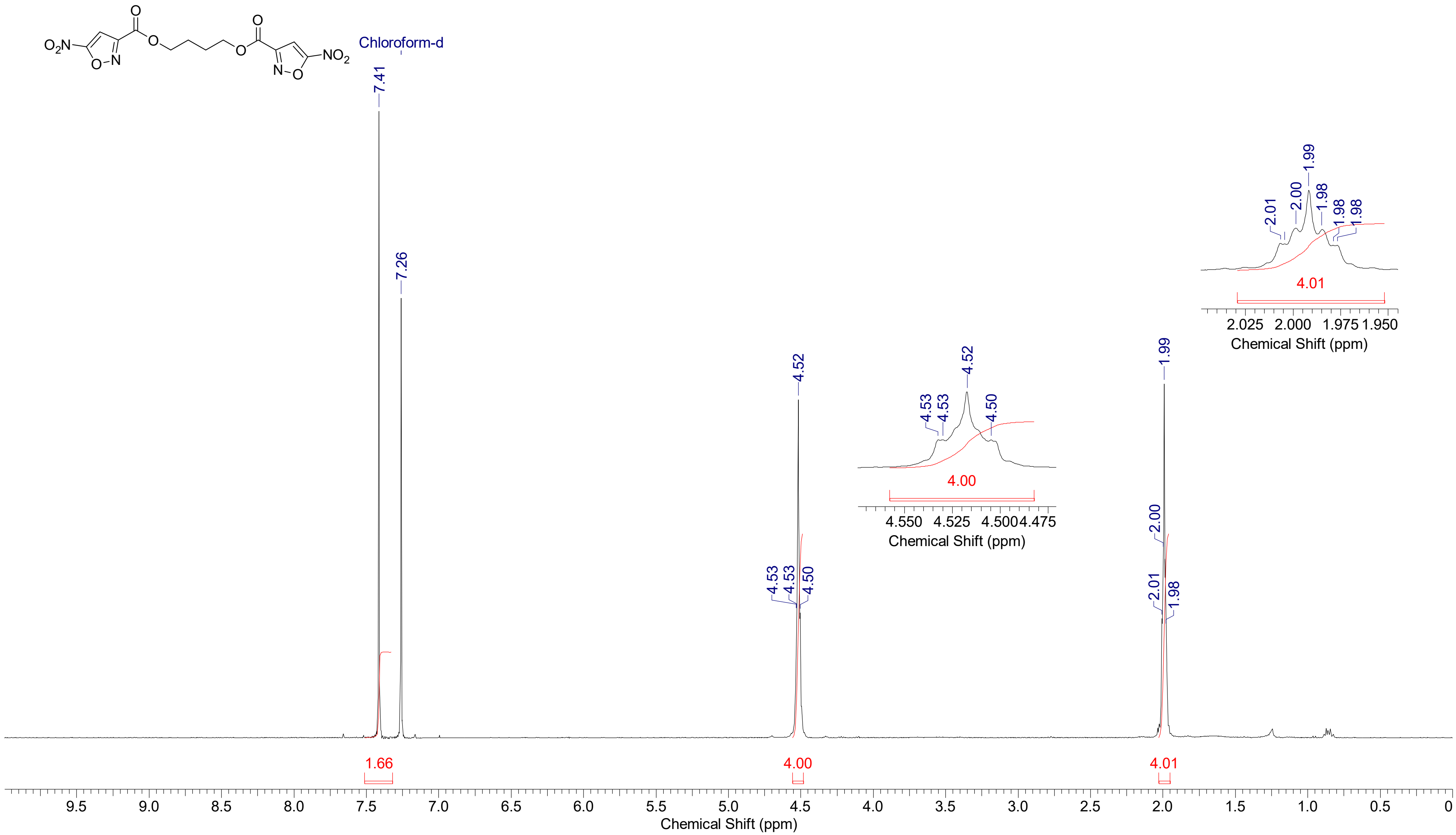

Butane-1,4-diyl bis(5-nitroisoxazole-3-carboxylate) **3c** (^{13}C NMR)

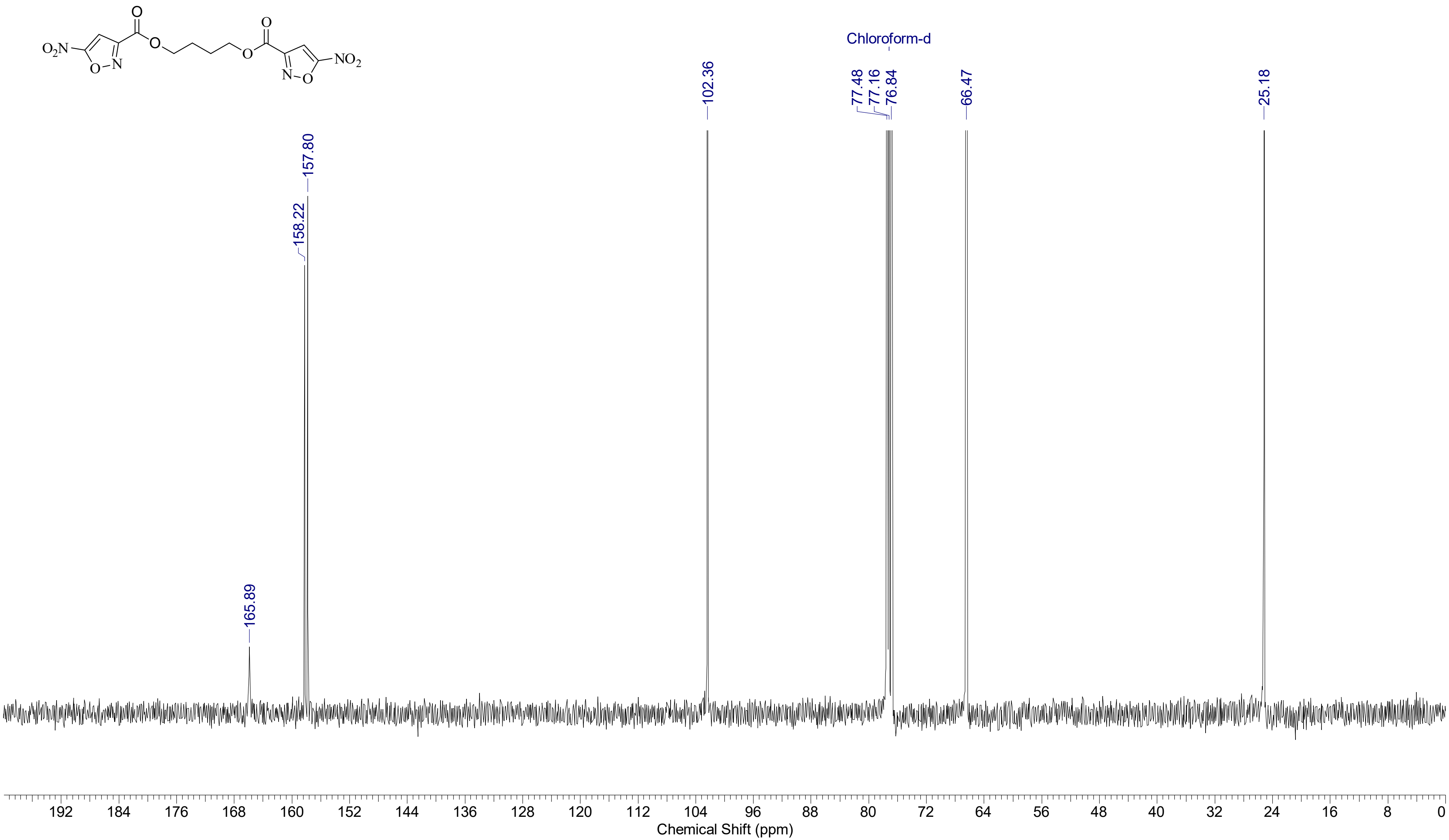

Butane-1,4-diyl bis(5-nitroisoxazole-3-carboxylate) **3c** (HMBC)

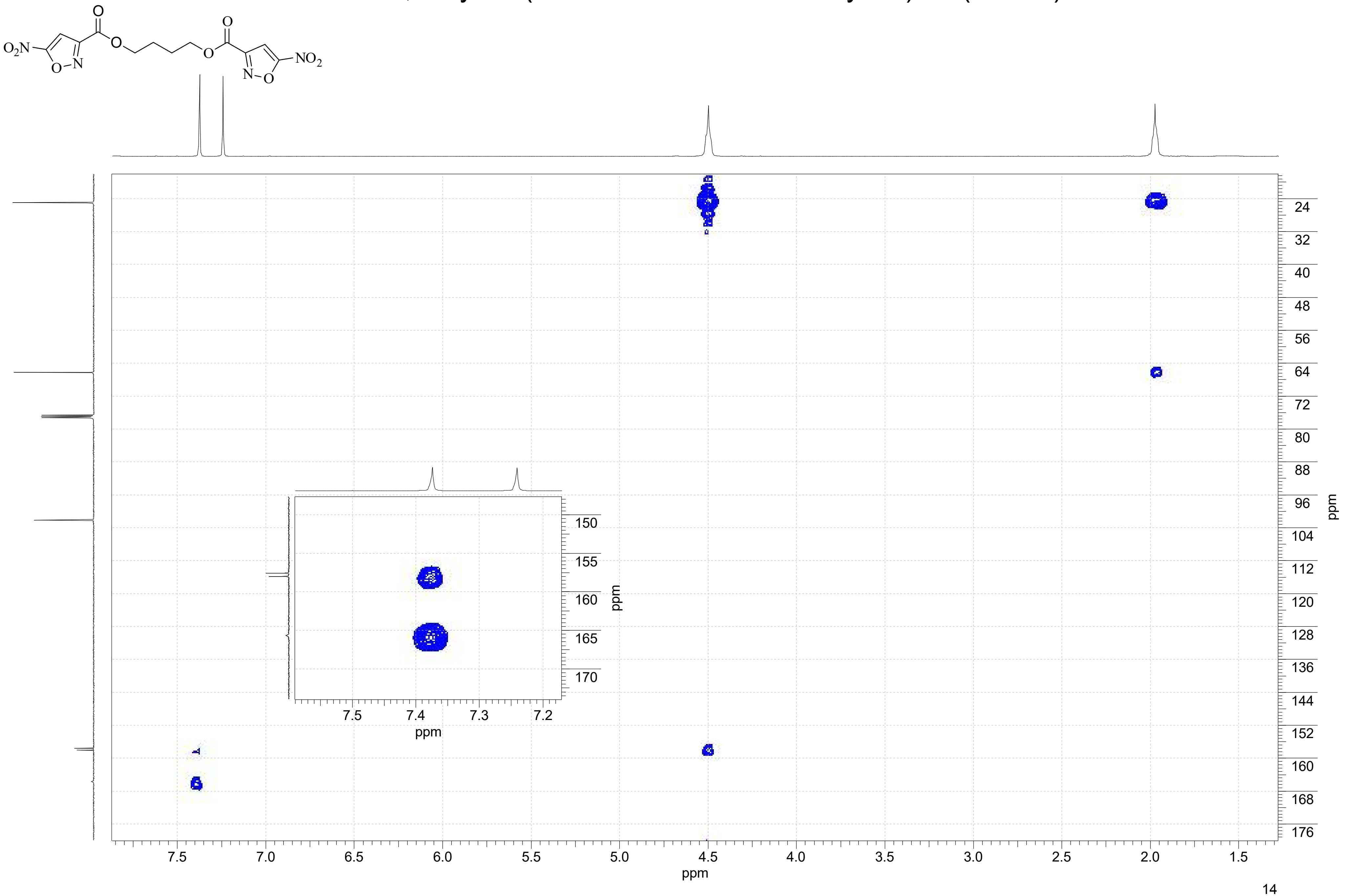

1,4-Phenylenedi(methylene) bis(5-nitroisoxazole-3-carboxylate) **3d** (^{1}H NMR)

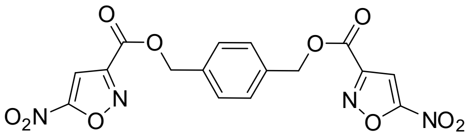

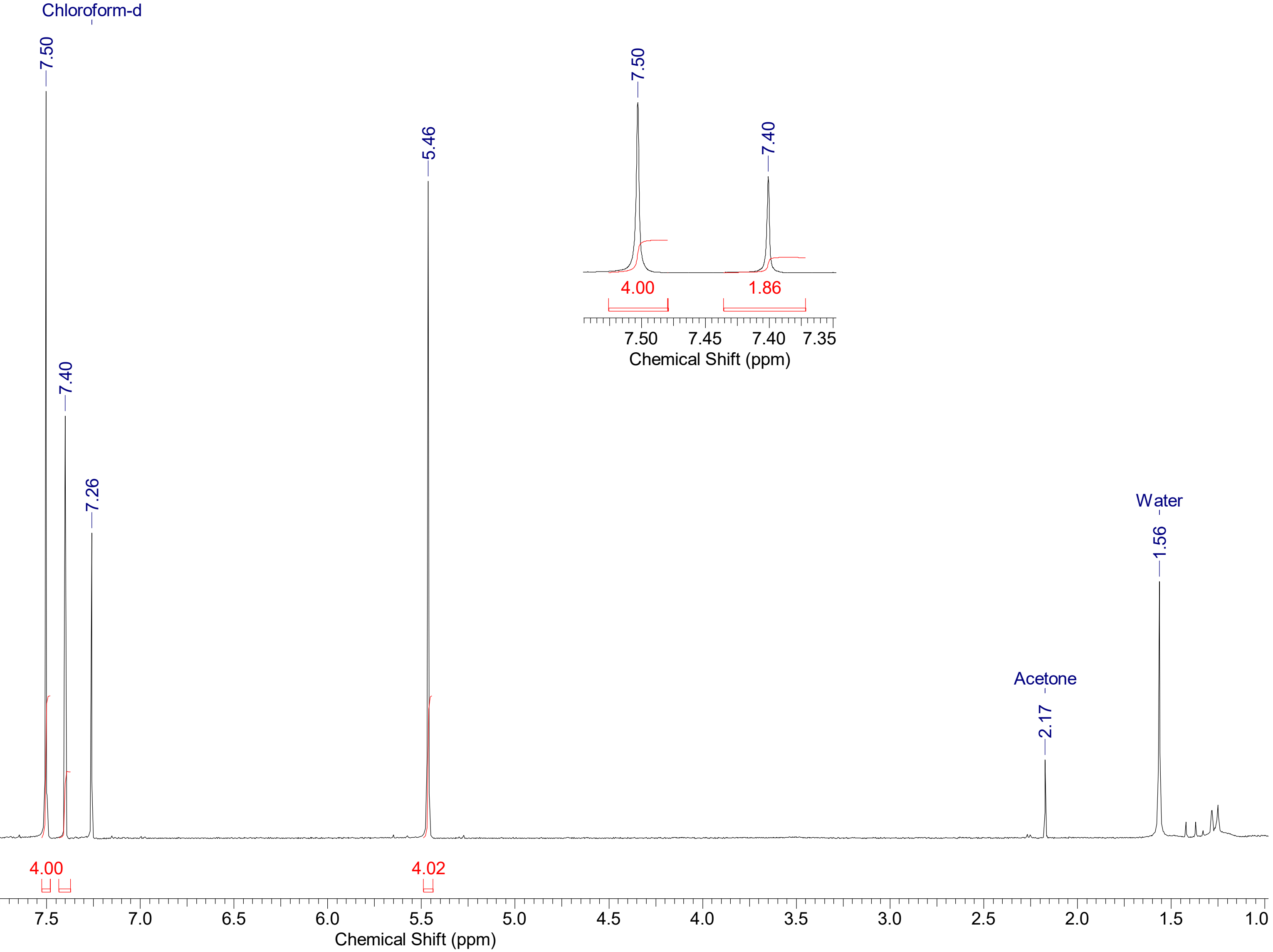

1,4-Phenylenedi(methylene) bis(5-nitroisoxazole-3-carboxylate) **3d** (^{13}C NMR)

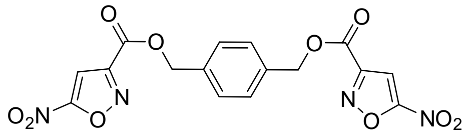

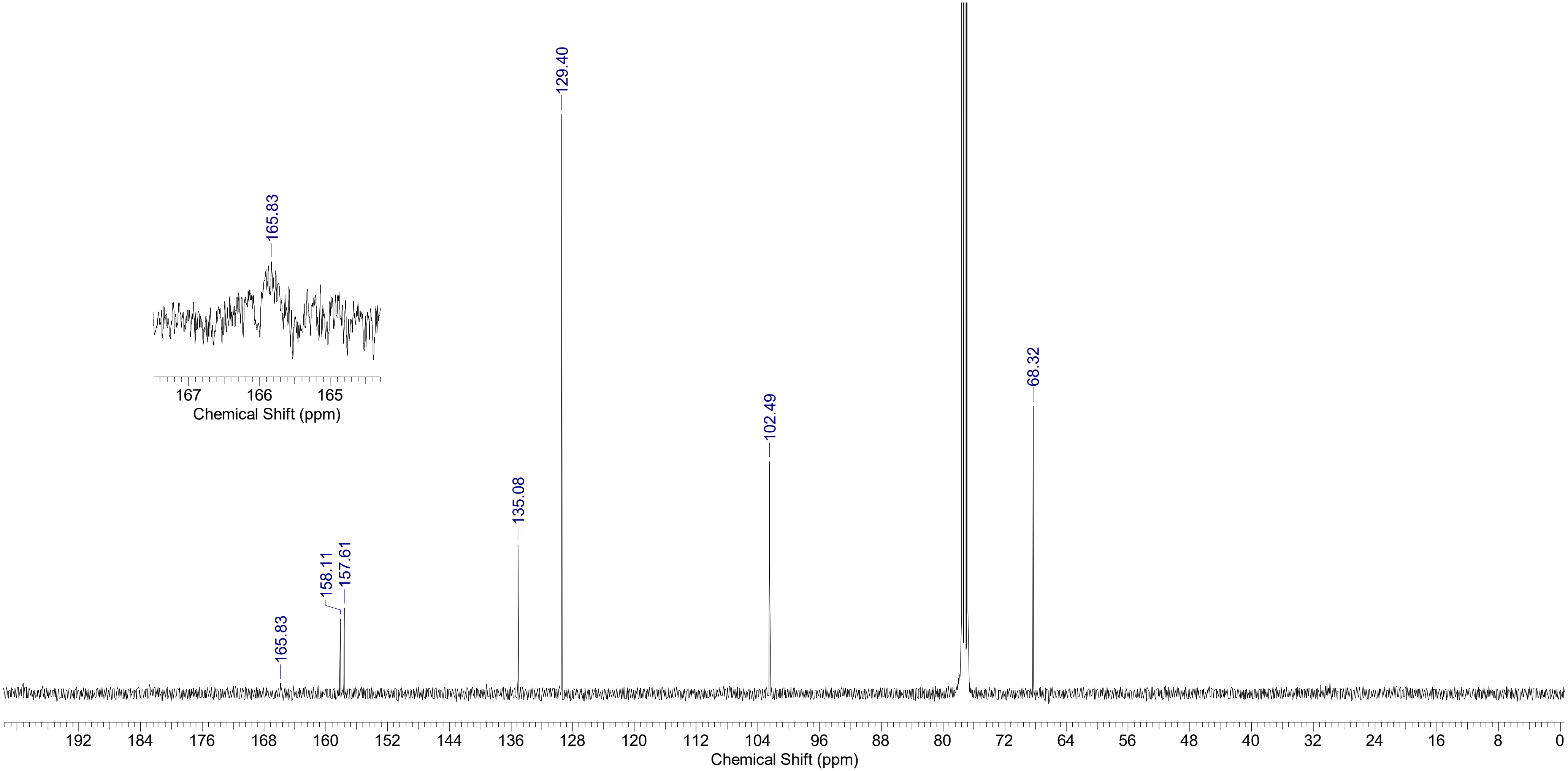

1,3-Phenylenedi(methylene) bis(5-nitroisoxazole-3-carboxylate) **3e** (^{1}H NMR)

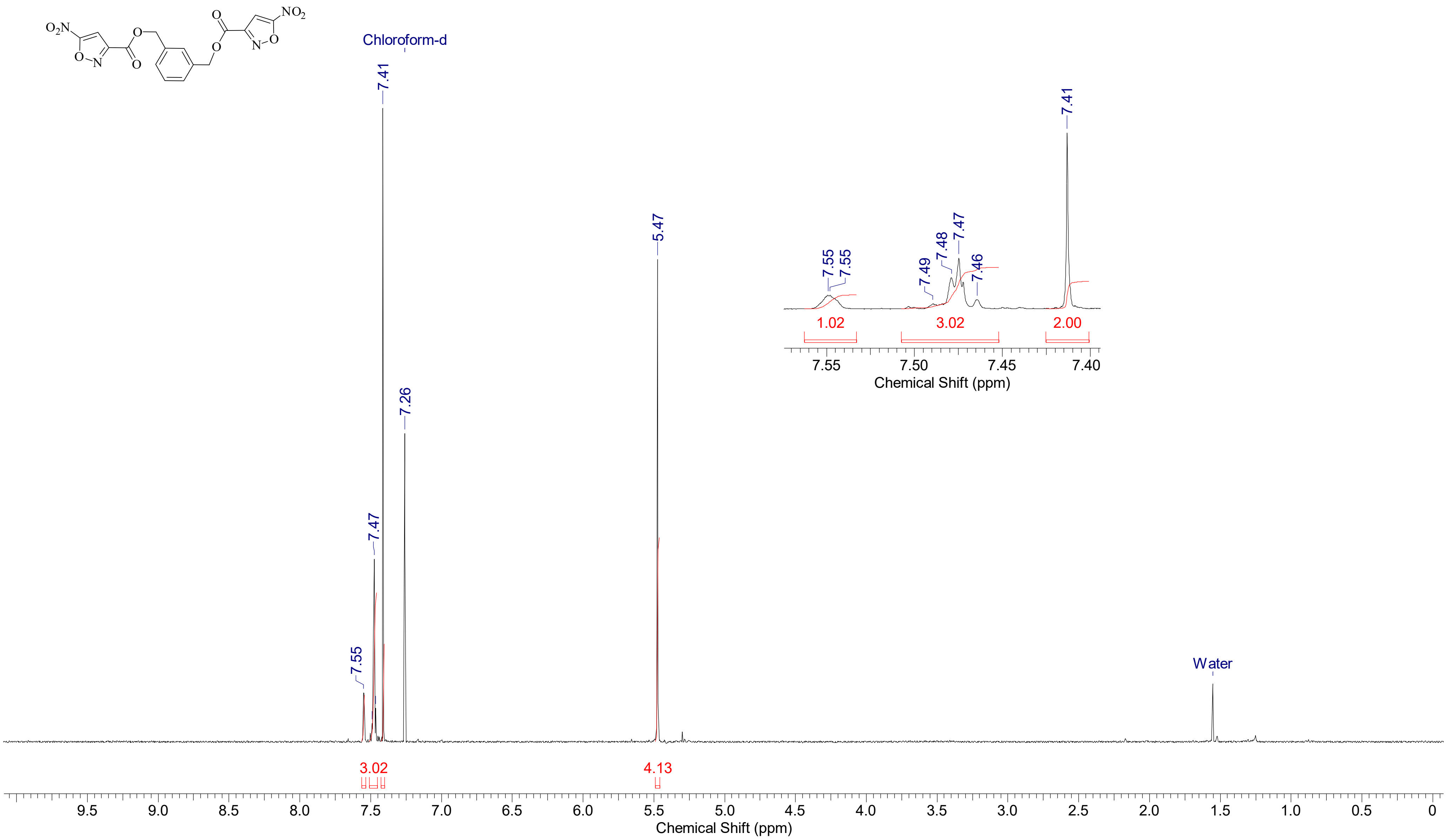

1,3-Phenylenedi(methylene) bis(5-nitroisoxazole-3-carboxylate) **3e** (^{13}C NMR)

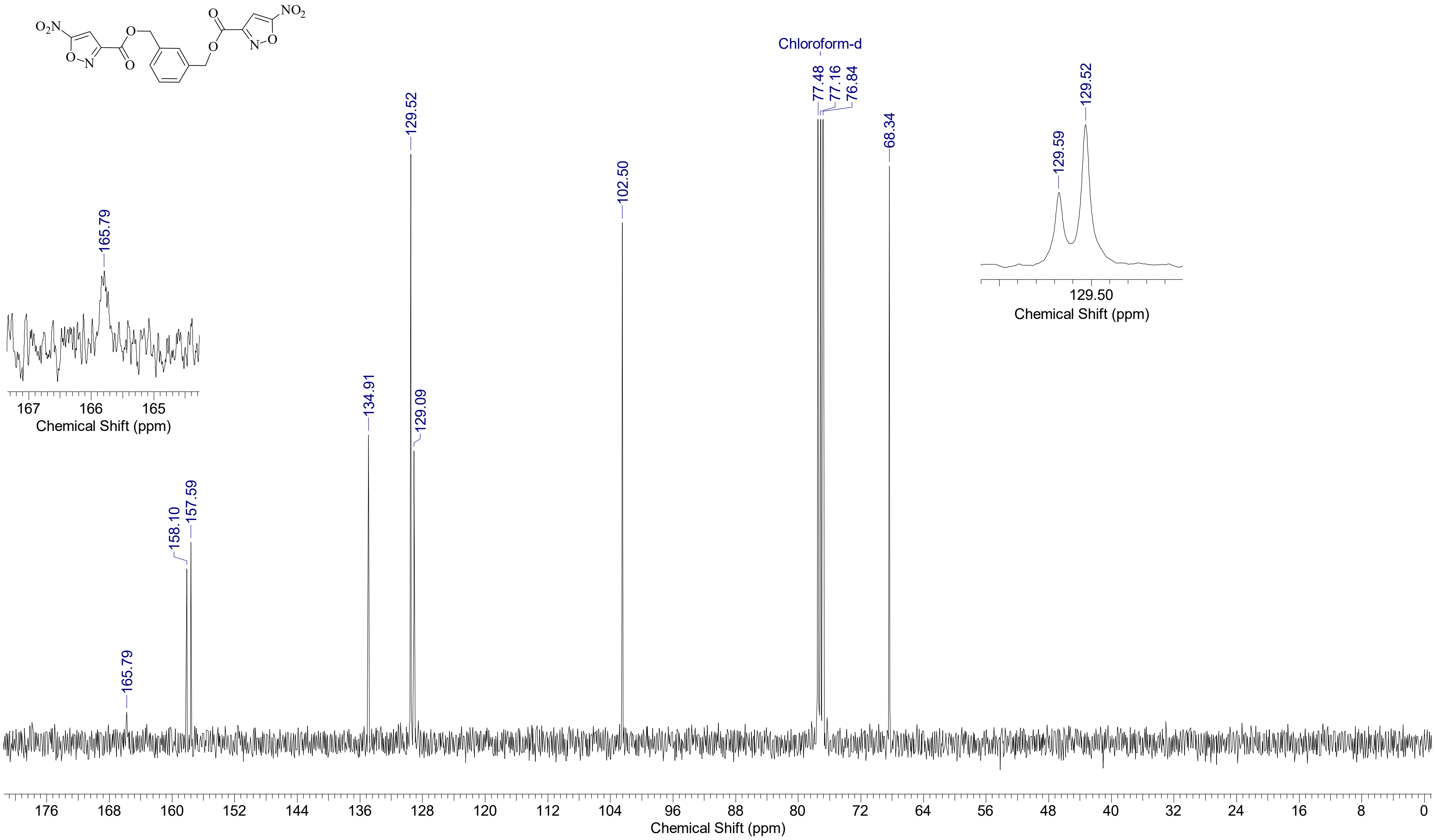

1,2-Phenylenedi(methylene) bis(5-nitroisoxazole-3-carboxylate) **3f** (^{1}H NMR)

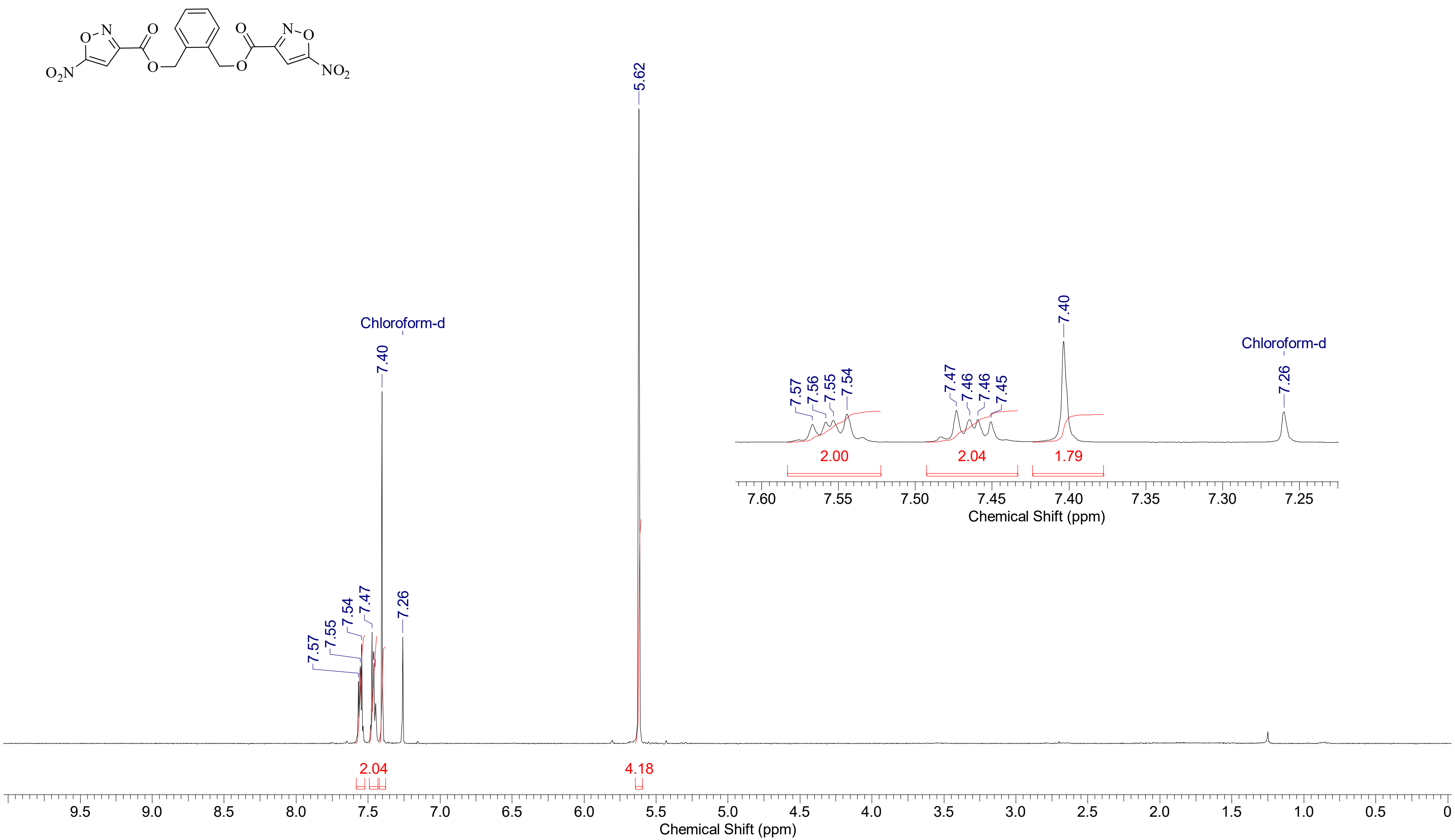

1,2-Phenylenedi(methylene) bis(5-nitroisoxazole-3-carboxylate) **3f** (^{13}C NMR)

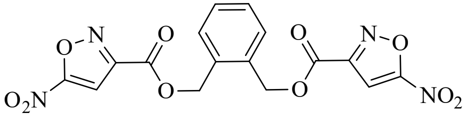

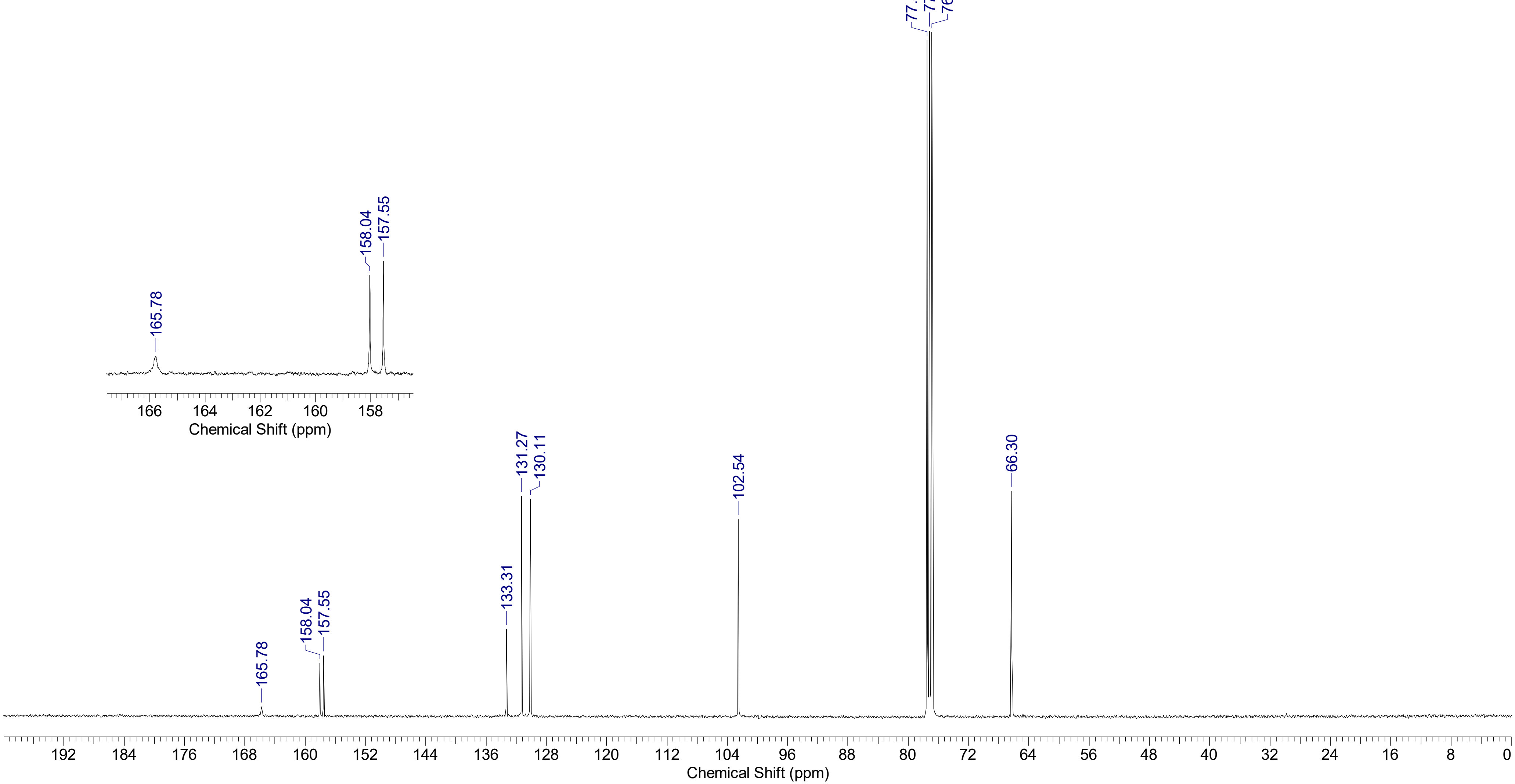

1,2-Phenylenedi(methylene) bis(5-nitroisoxazole-3-carboxylate) **3f** (HMBC)

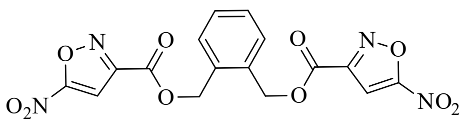

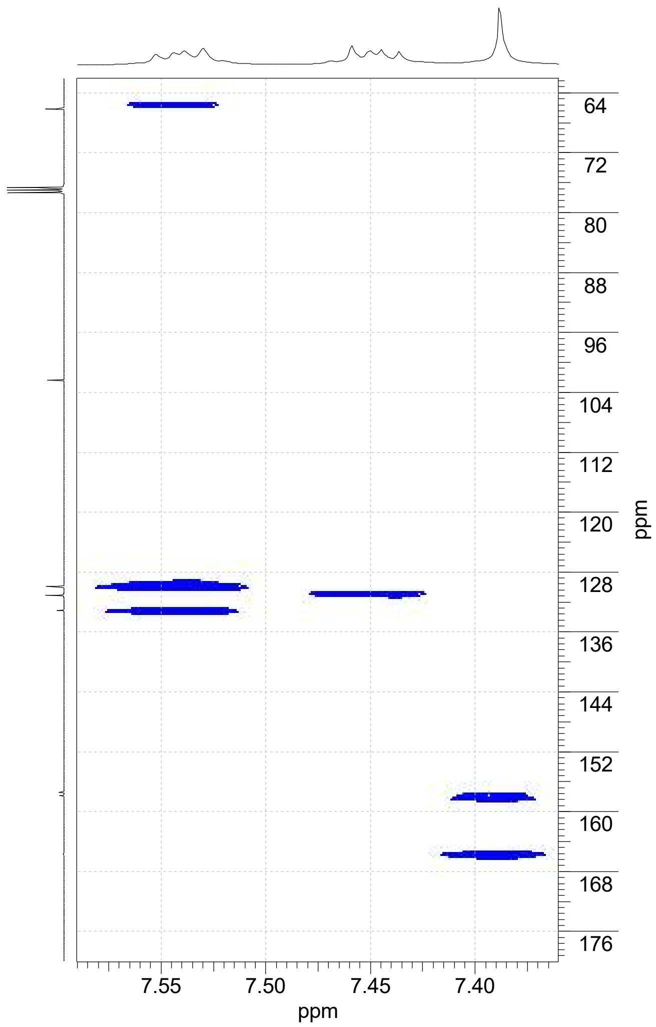

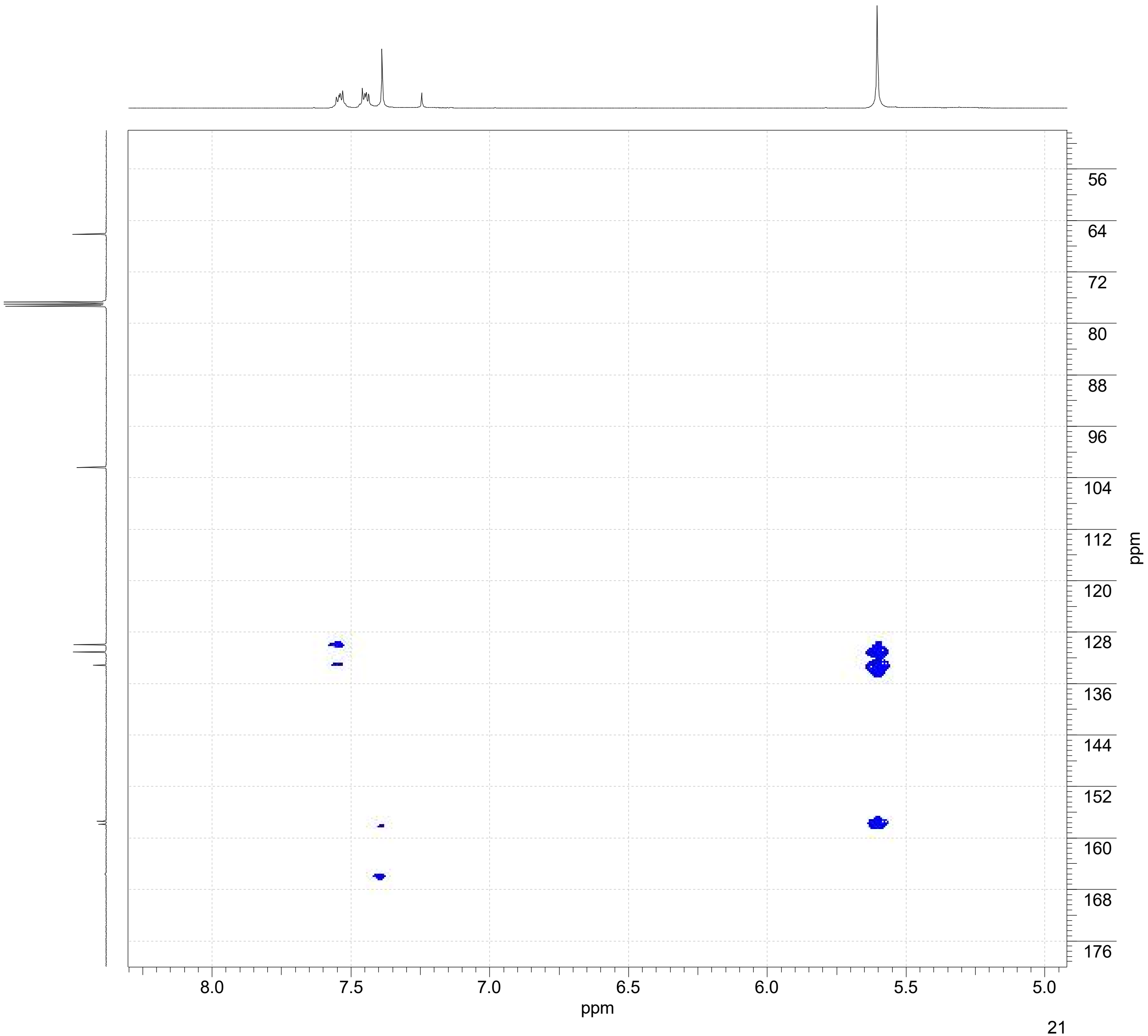

Adamantane-1,3-diyldi(methylene) bis(5-nitroisoxazole-3-carboxylate) **3g** (^{1}H NMR)

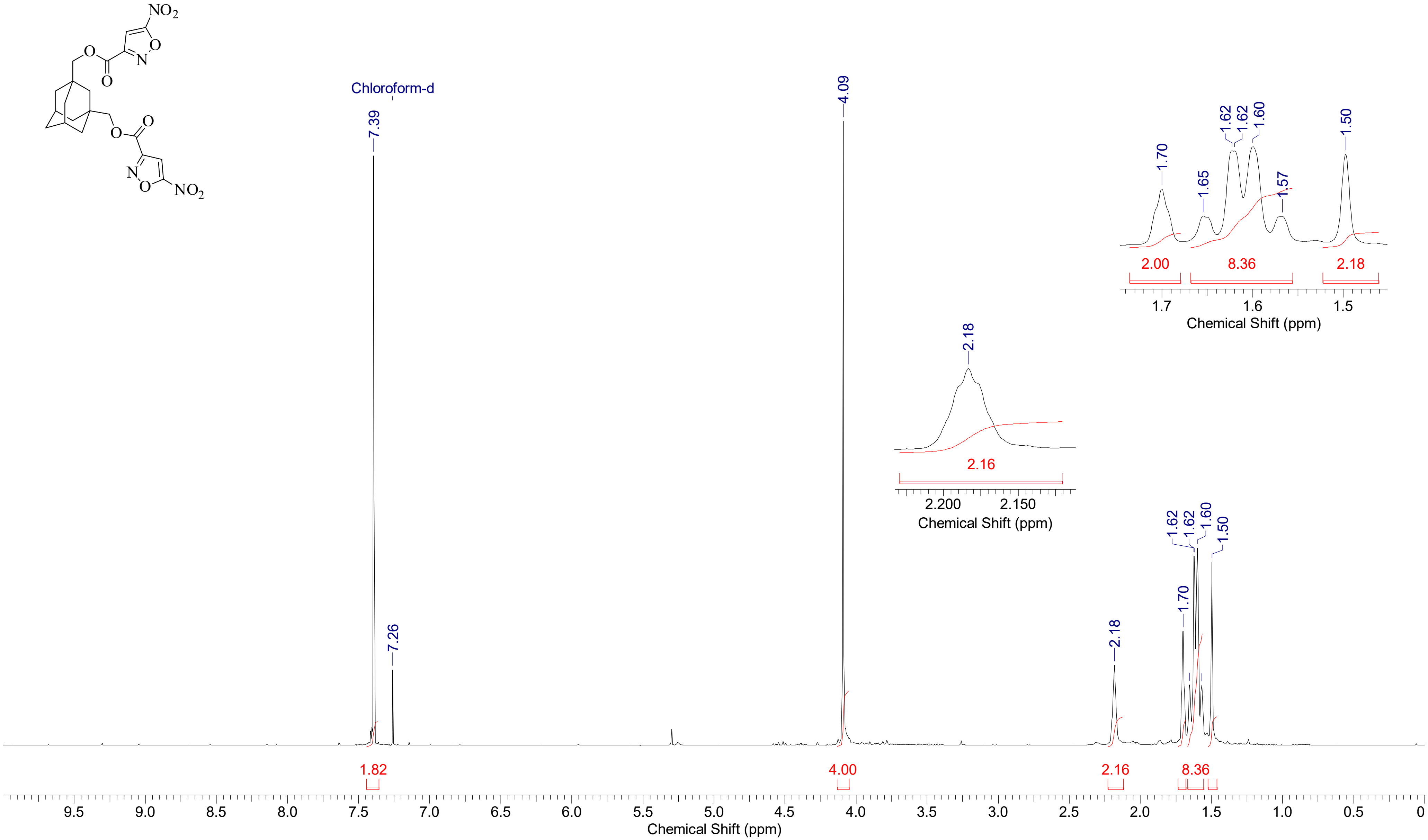

Adamantane-1,3-diyldi(methylene) bis(5-nitroisoxazole-3-carboxylate) **3g** (^{13}C NMR)

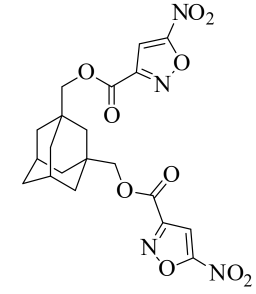

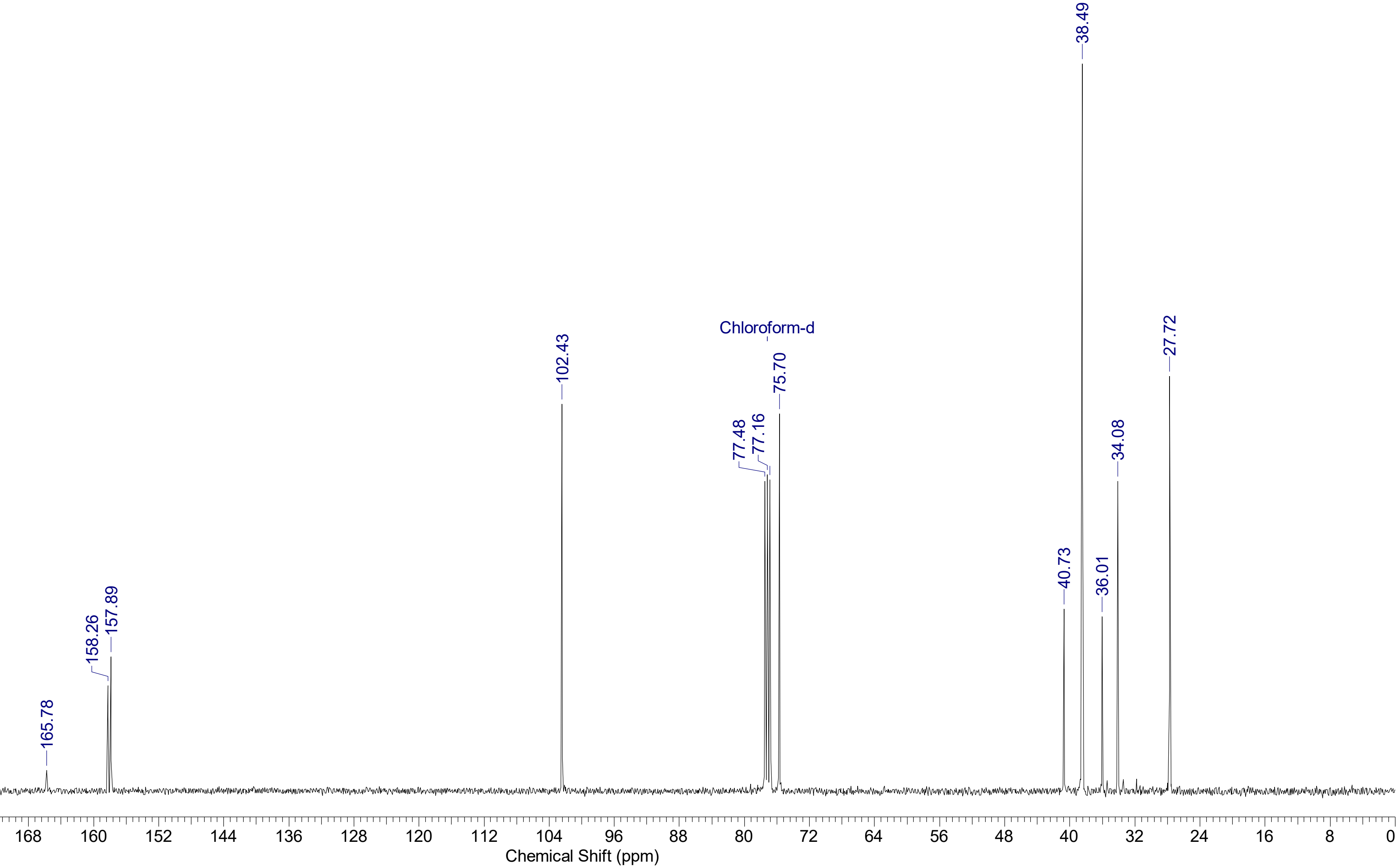

Adamantane-1,3-diyldi(methylene) bis(5-nitroisoxazole-3-carboxylate) **3g** (APT)

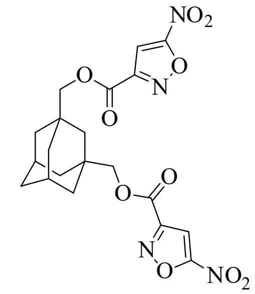

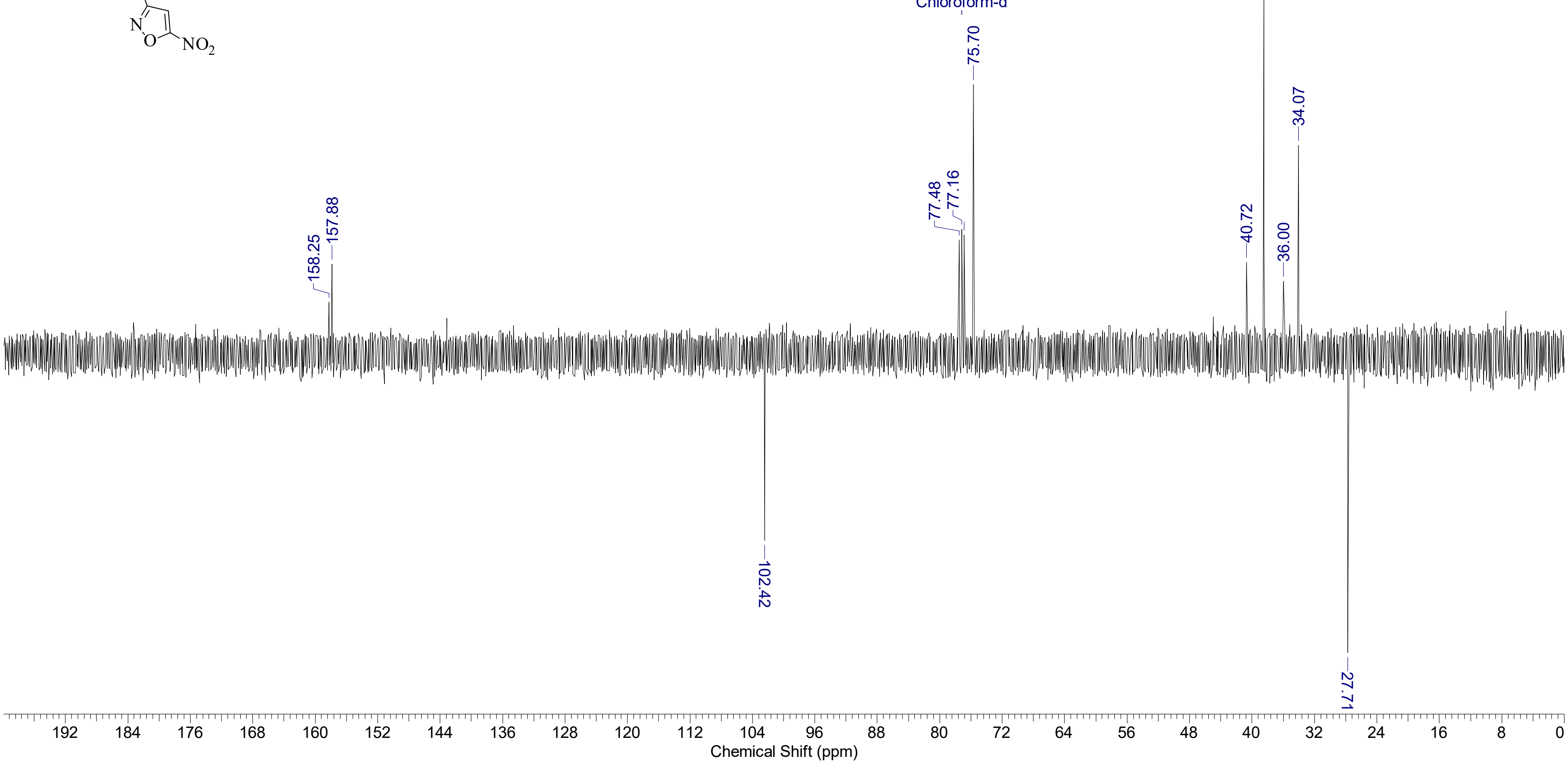

Adamantane-1,3-diyldi(methylene) bis(5-nitroisoxazole-3-carboxylate) **3g**

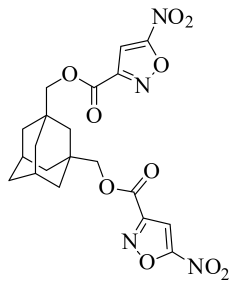

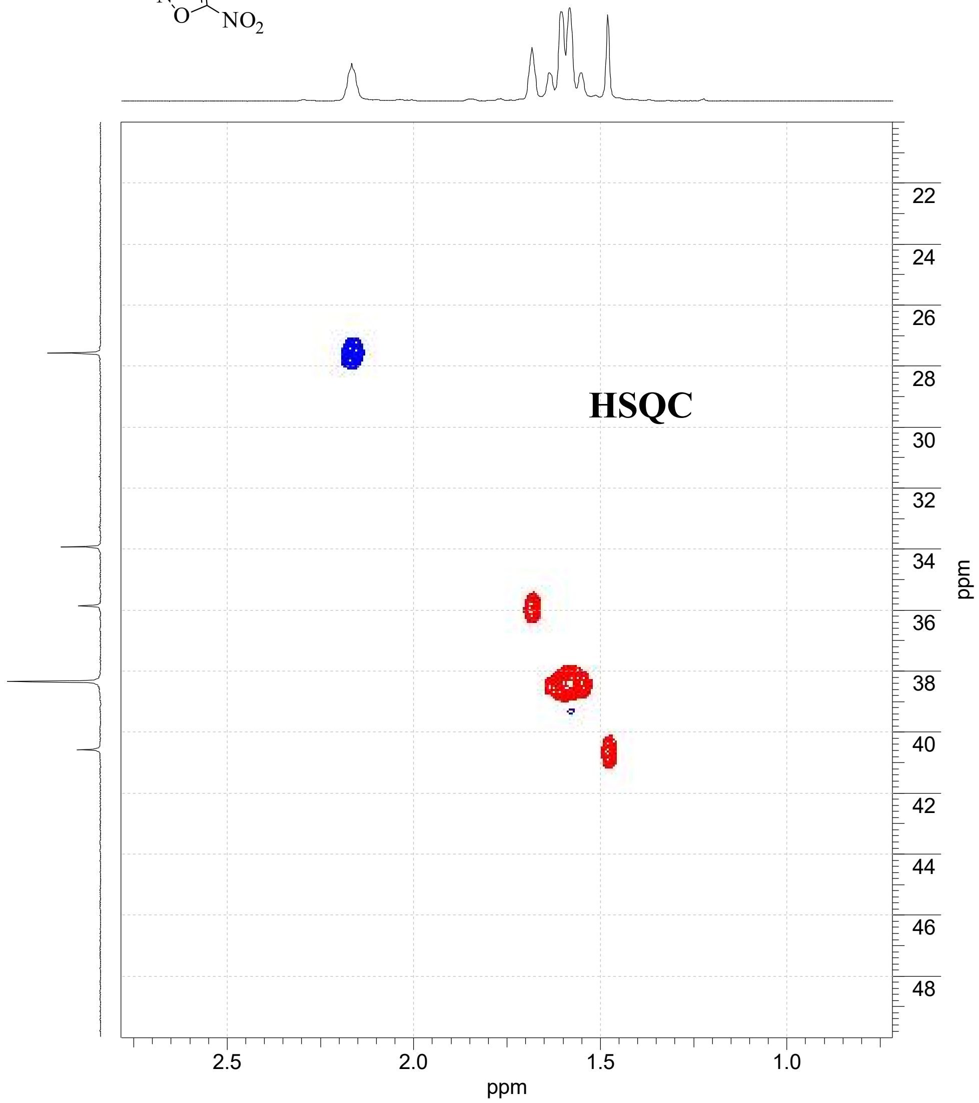

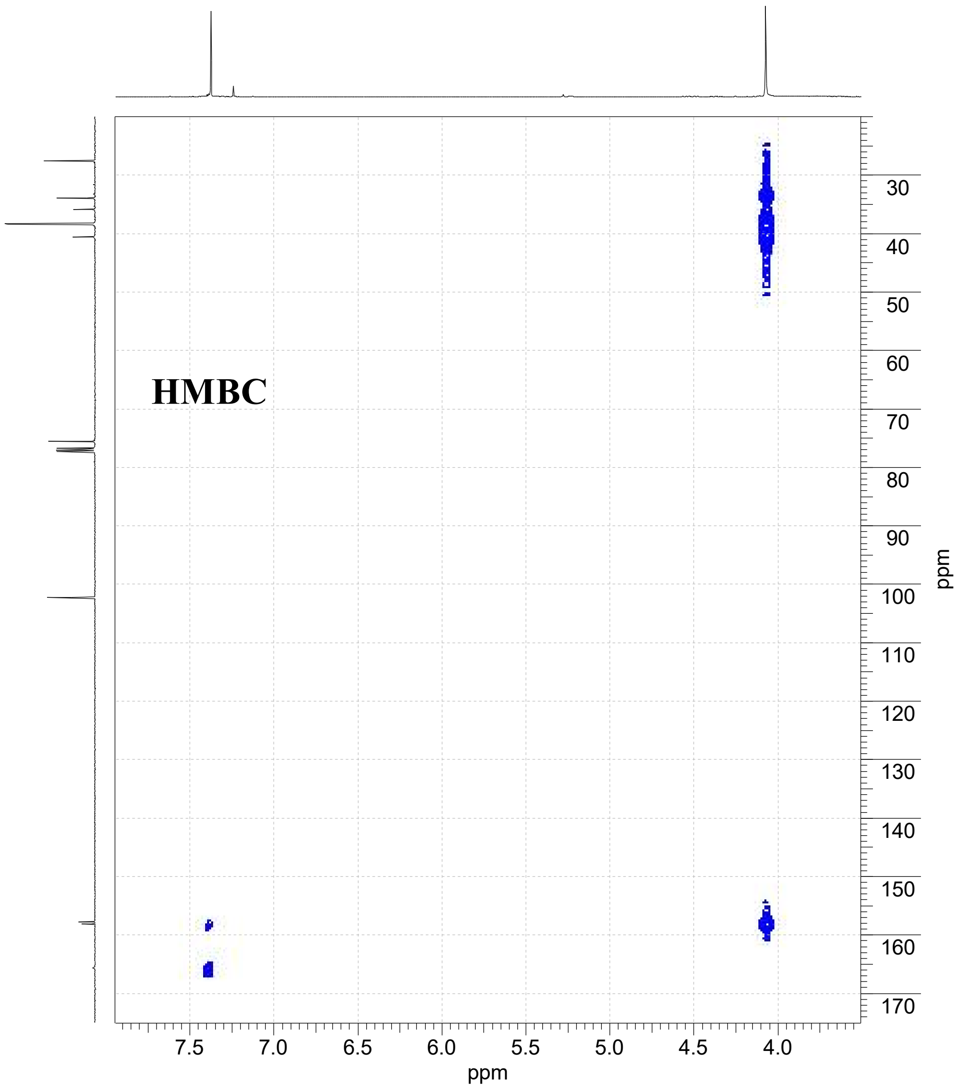

((3*s*,7*s*)-7-{[(5-Nitroisoxazol-3-yl)carbonyl]oxy}bicyclo[3.3.1]non-3-yl)methyl 5-nitroisoxazole-3-carboxylate **3h** (^{1}H NMR)

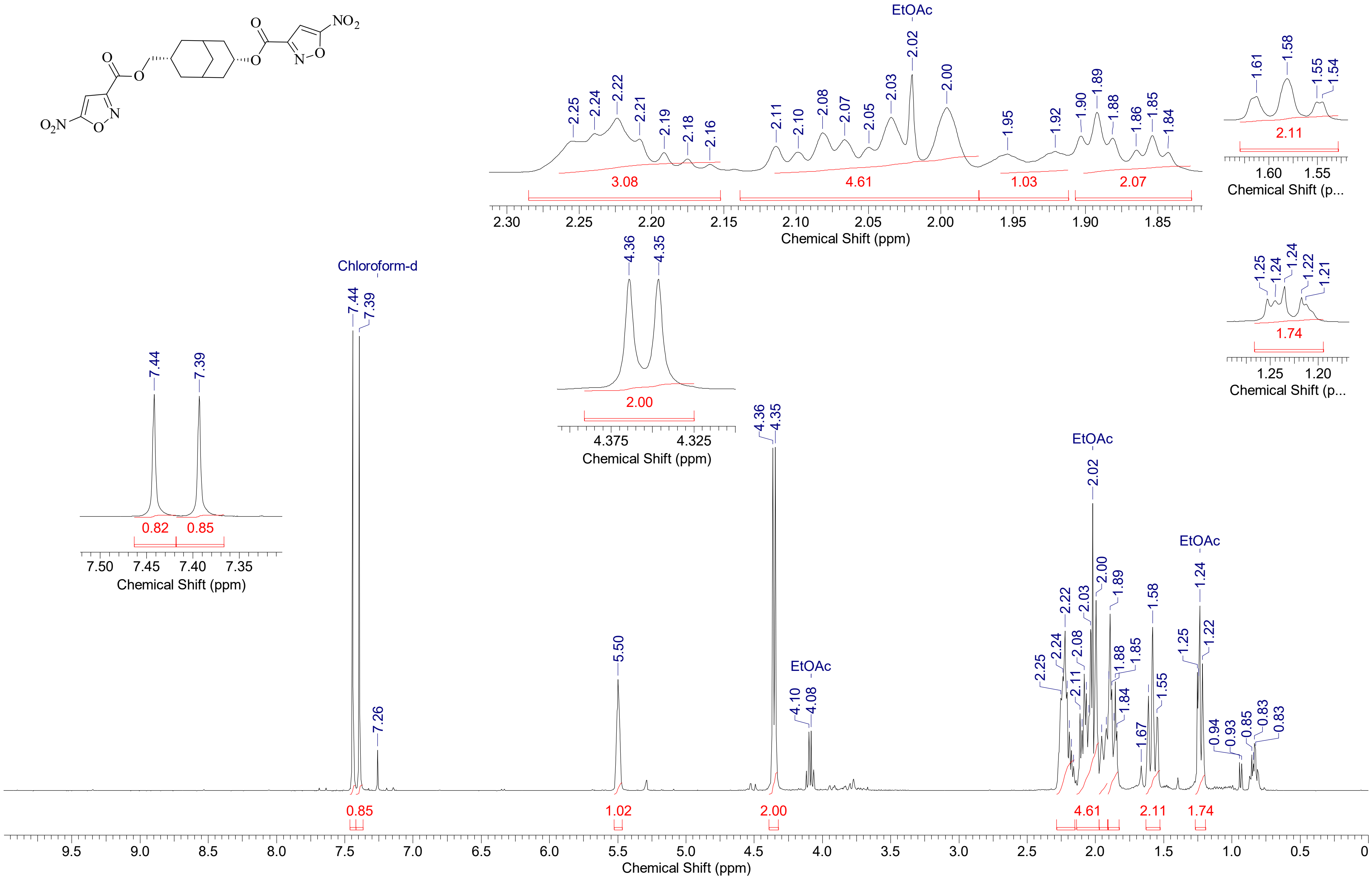

((3*s*,7*s*)-7-{[(5-Nitroisoxazol-3-yl)carbonyl]oxy}bicyclo[3.3.1]non-3-yl)methyl 5-nitroisoxazole-3-carboxylate **3h** (^{13}C NMR)

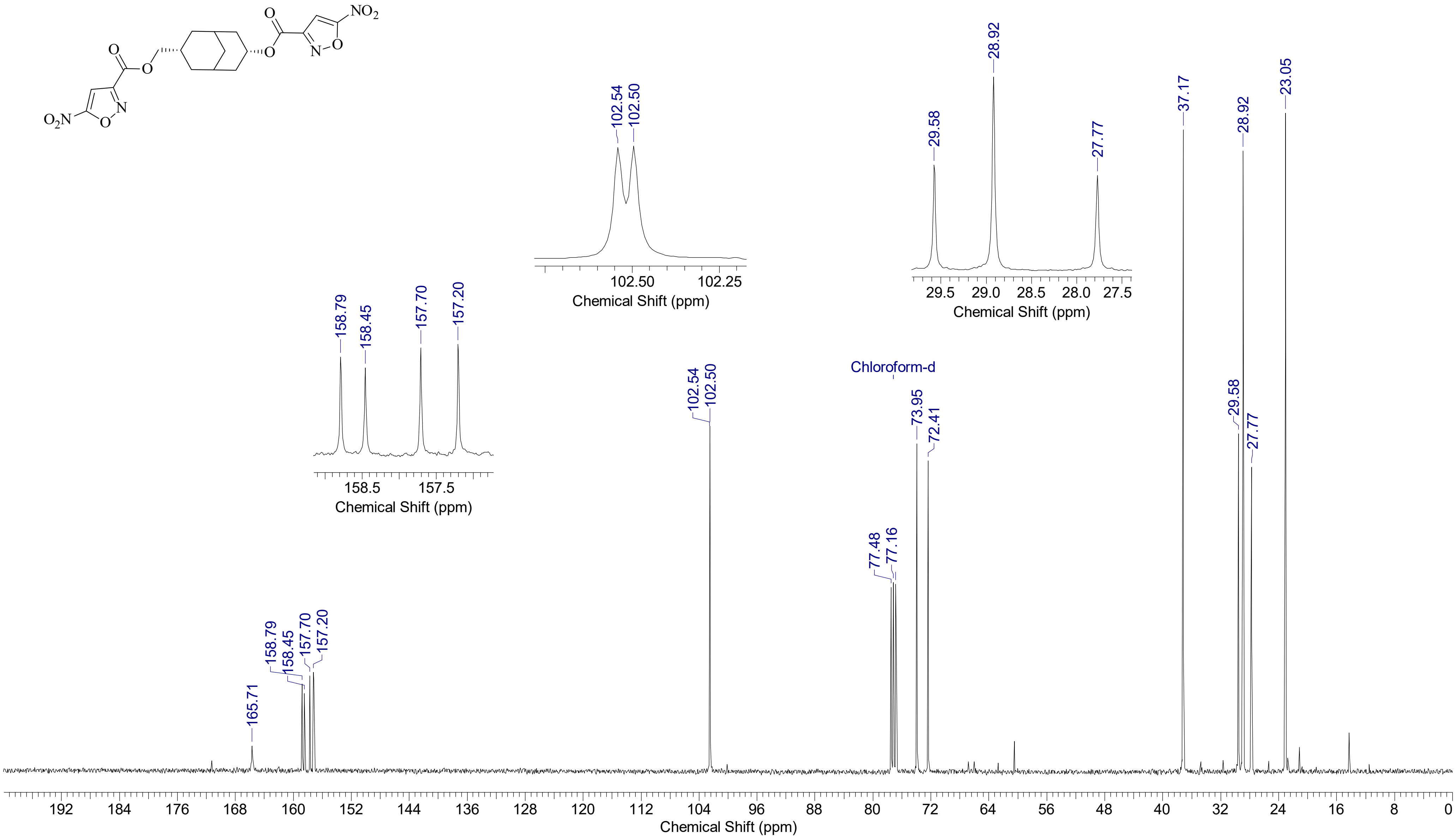

((3*s*,7*s*)-7-{[(5-Nitroisoxazol-3-yl)carbonyl]oxy}bicyclo[3.3.1]non-3-yl)methyl 5-nitroisoxazole-3-carboxylate **3h** (HSQC)

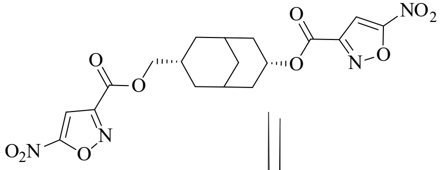

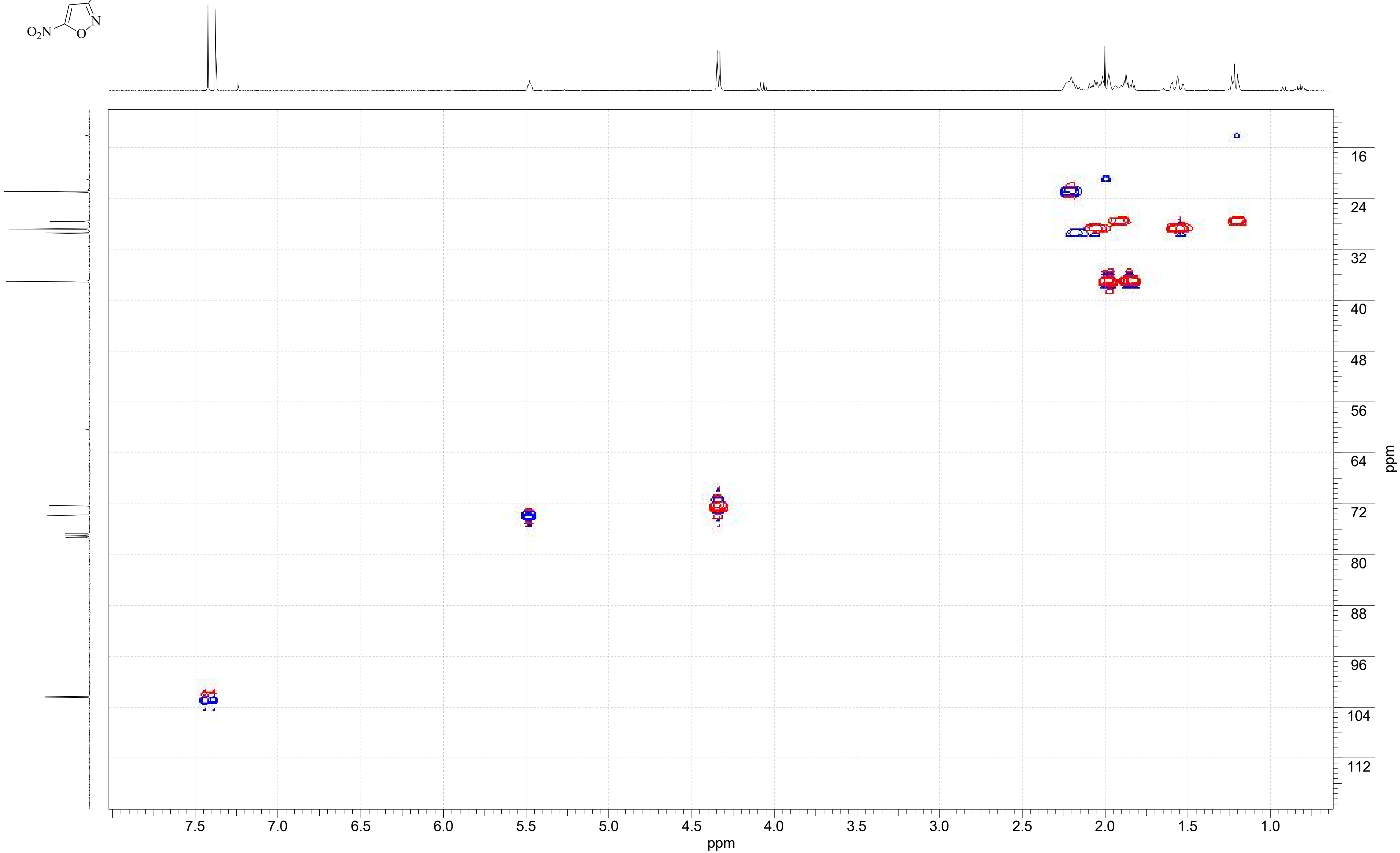

((3*s*,7*s*)-7-{[(5-Nitroisoxazol-3-yl)carbonyl]oxy}bicyclo[3.3.1]non-3-yl)methyl 5-nitroisoxazole-3-carboxylate **3h** (HMBC)

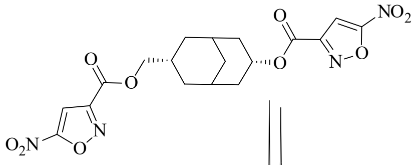

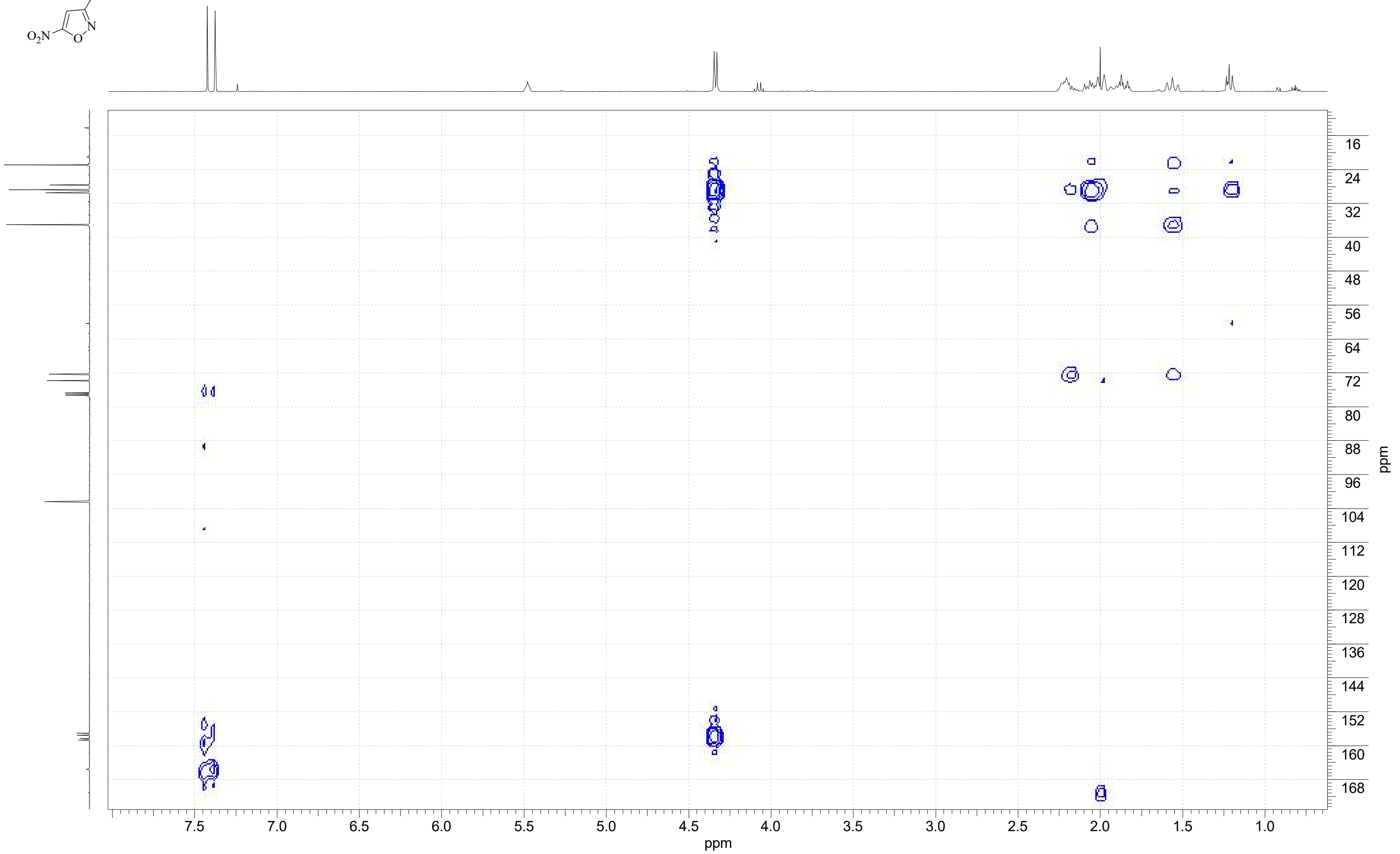

((*1r,3s,5R,7S*)-3(((Cyanocarbonyl)oxy)methyl)adamantan-1-yl)methyl 5-nitroisoxazole-3-carboxylate, **4** (^{1}H NMR)

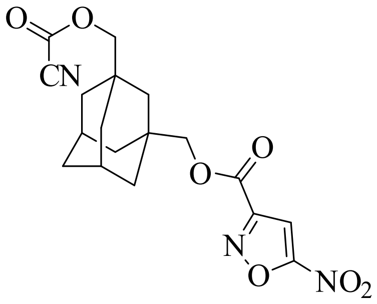

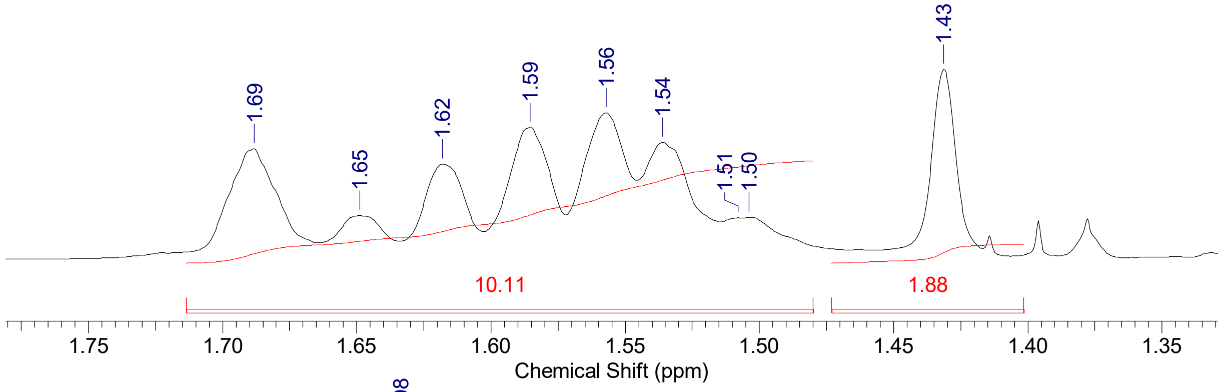

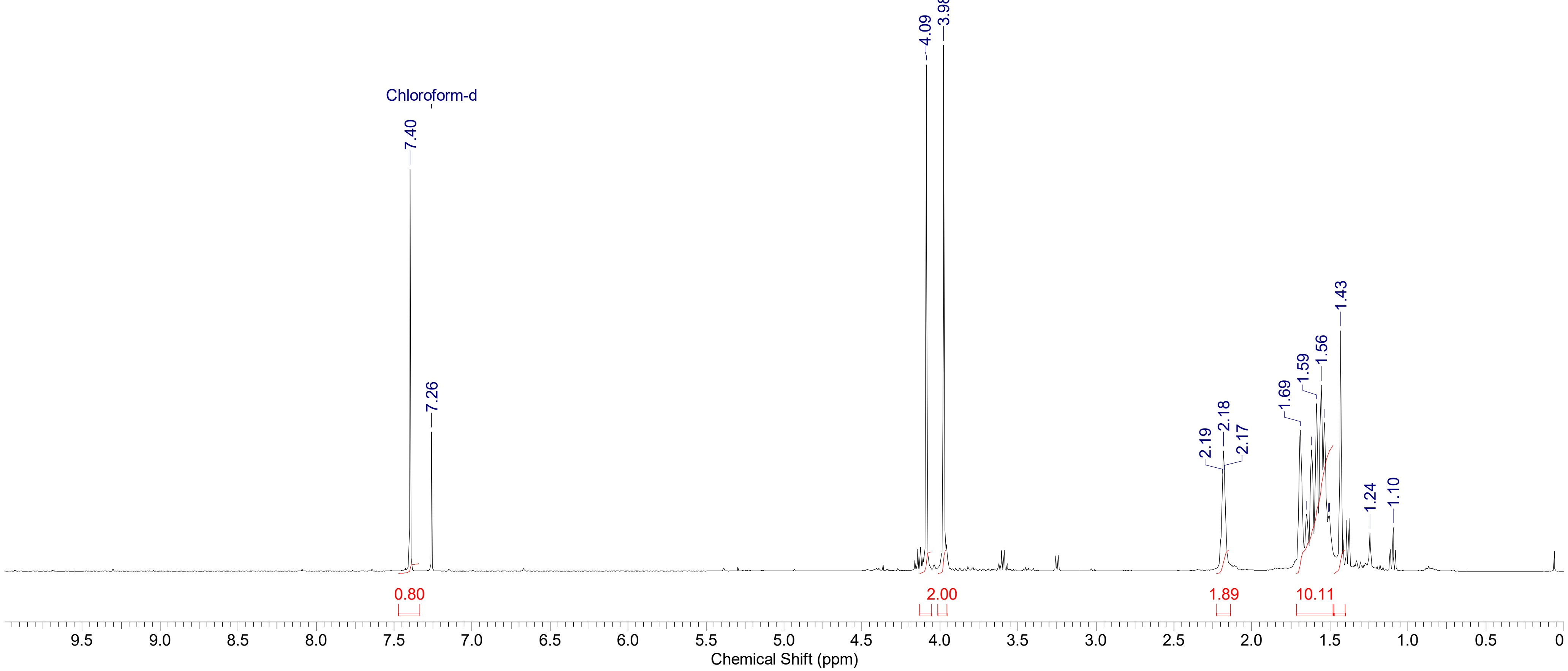

((*1r,3s,5R,7S*)-3(((Cyanocarbonyl)oxy)methyl)adamantan-1-yl)methyl 5-nitroisoxazole-3-carboxylate, **4** (^{13}C NMR)

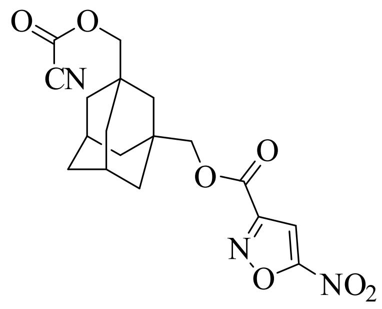

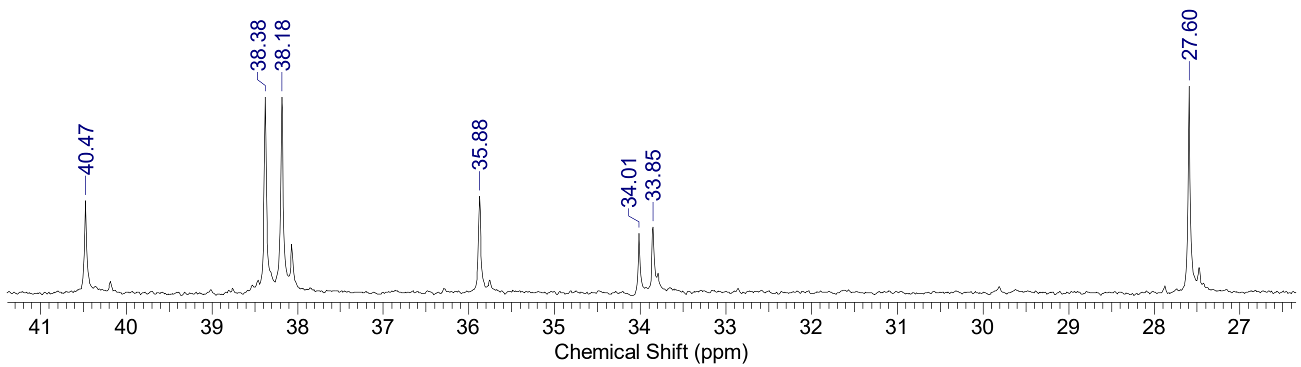

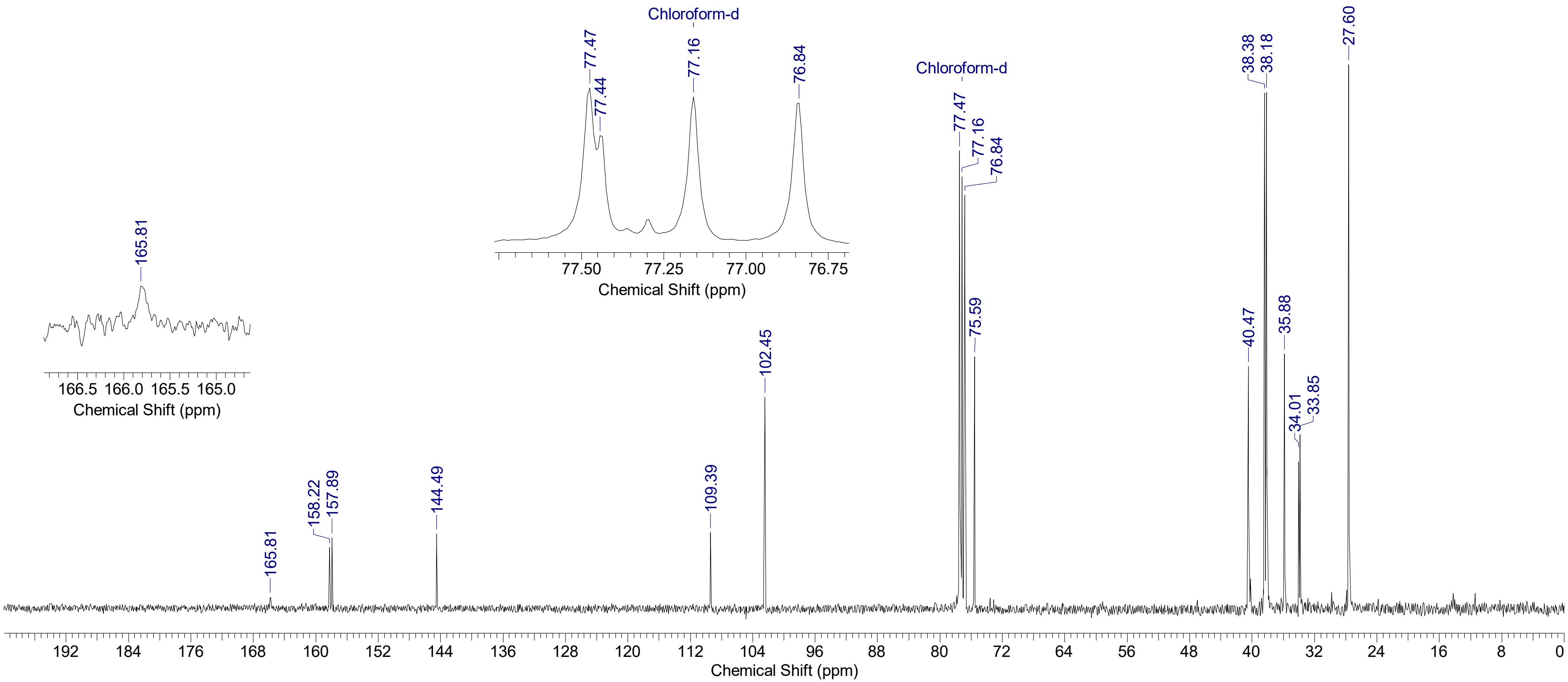

((*1r,3s,5R,7S*)-3(((Cyanocarbonyl)oxy)methyl)adamantan-1-yl)methyl 5-nitroisoxazole-3-carboxylate, **4** (HSQC)

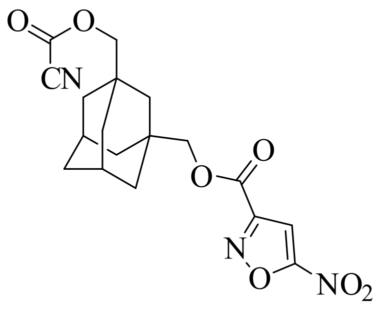

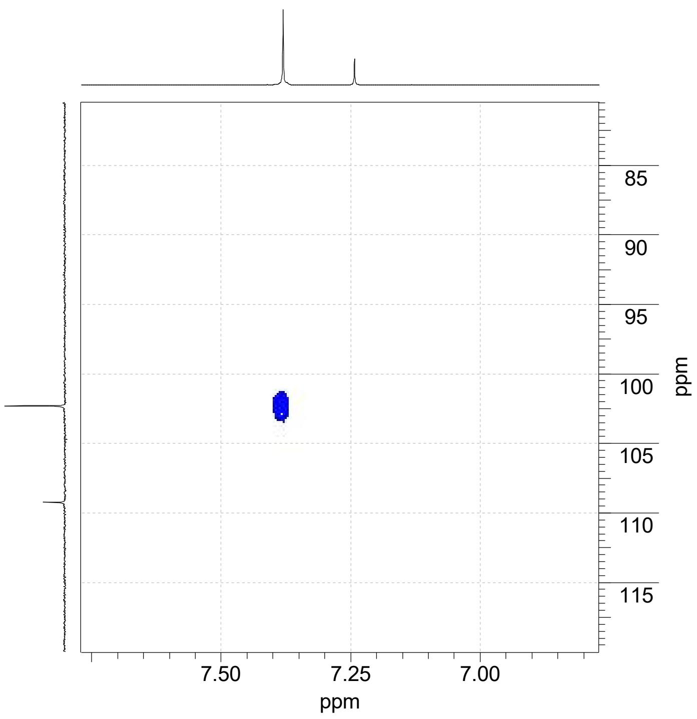

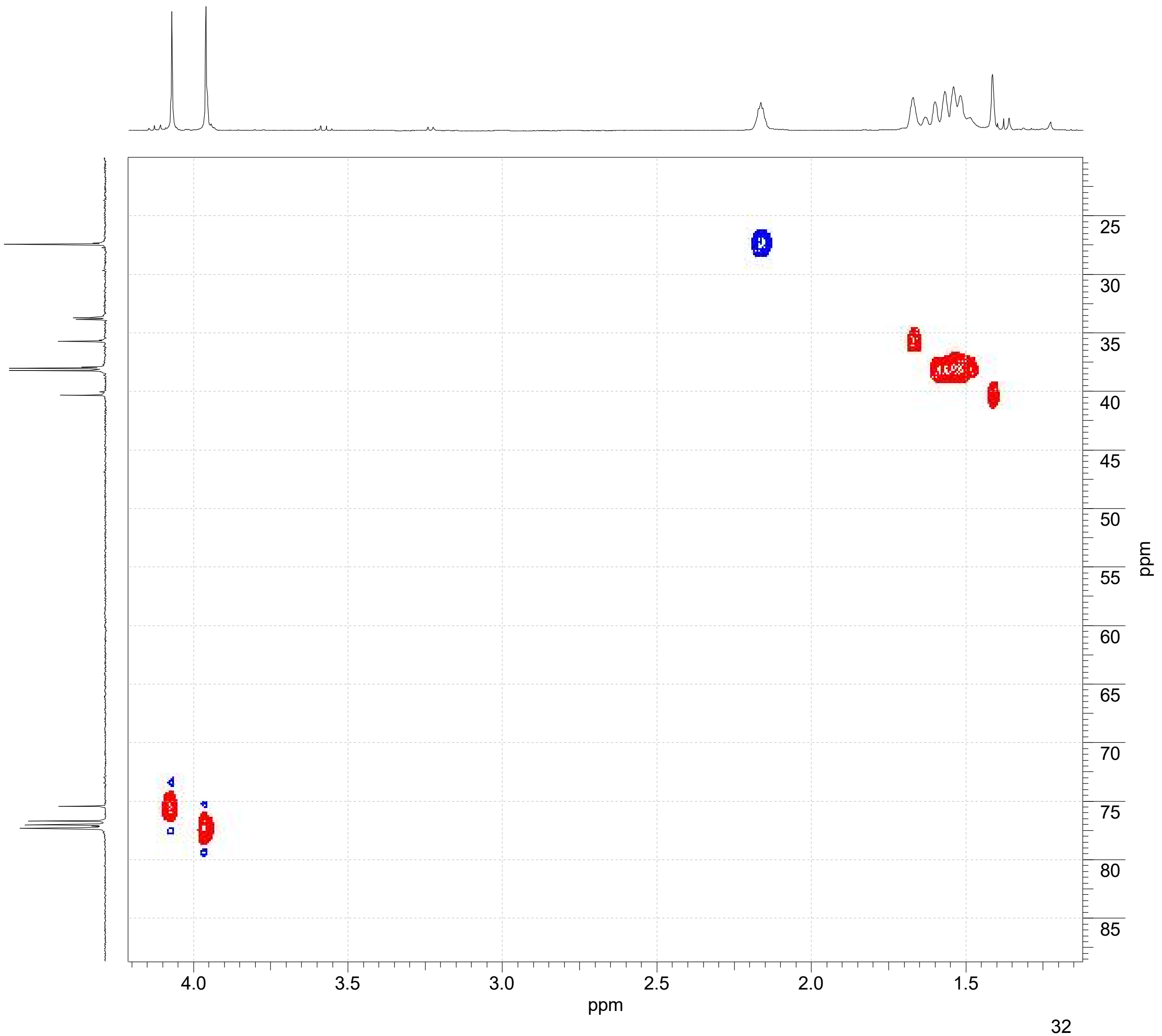

1,4-Phenylene bis(4,4,4-trinitrobutanoate) **5a** (^{1}H NMR)

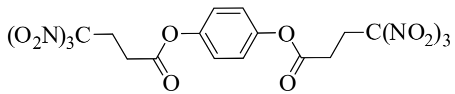

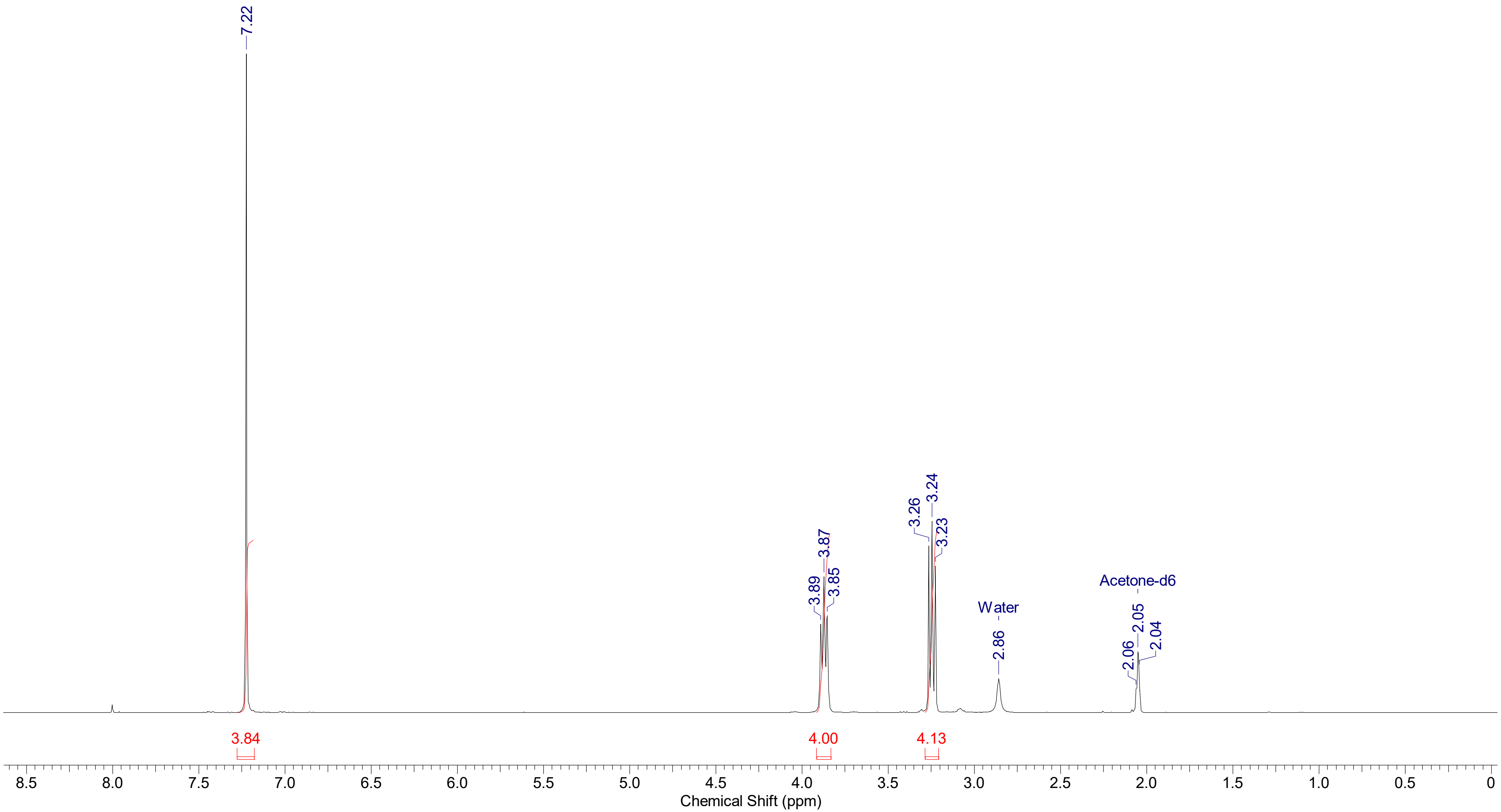

1,4-Phenylene bis(4,4,4-trinitrobutanoate) **5a** (^{13}C NMR)

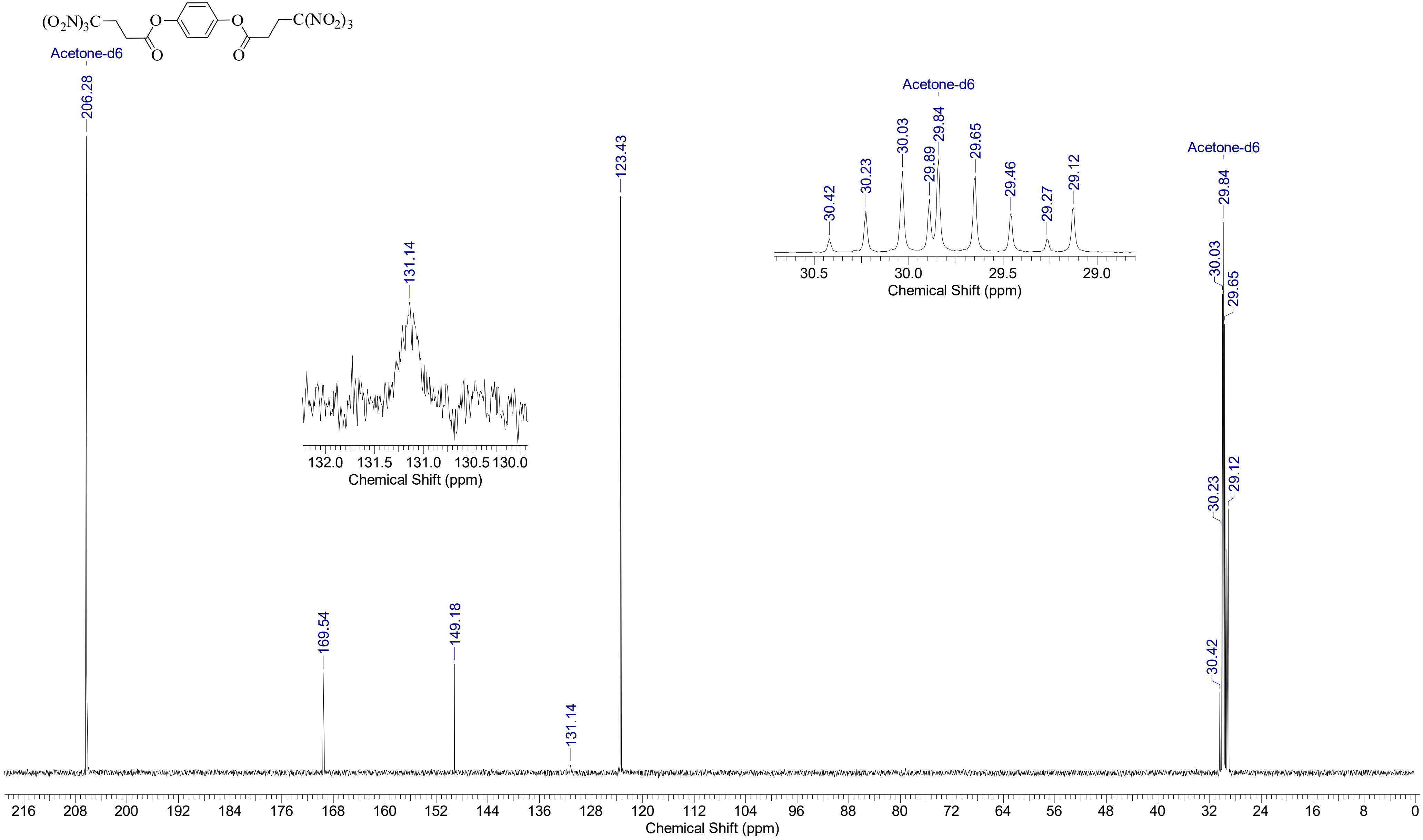

1,4-Phenylene bis(4,4,4-trinitrobutanoate) **5a** (APT)

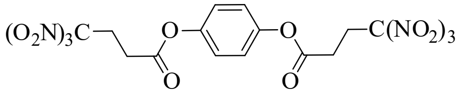

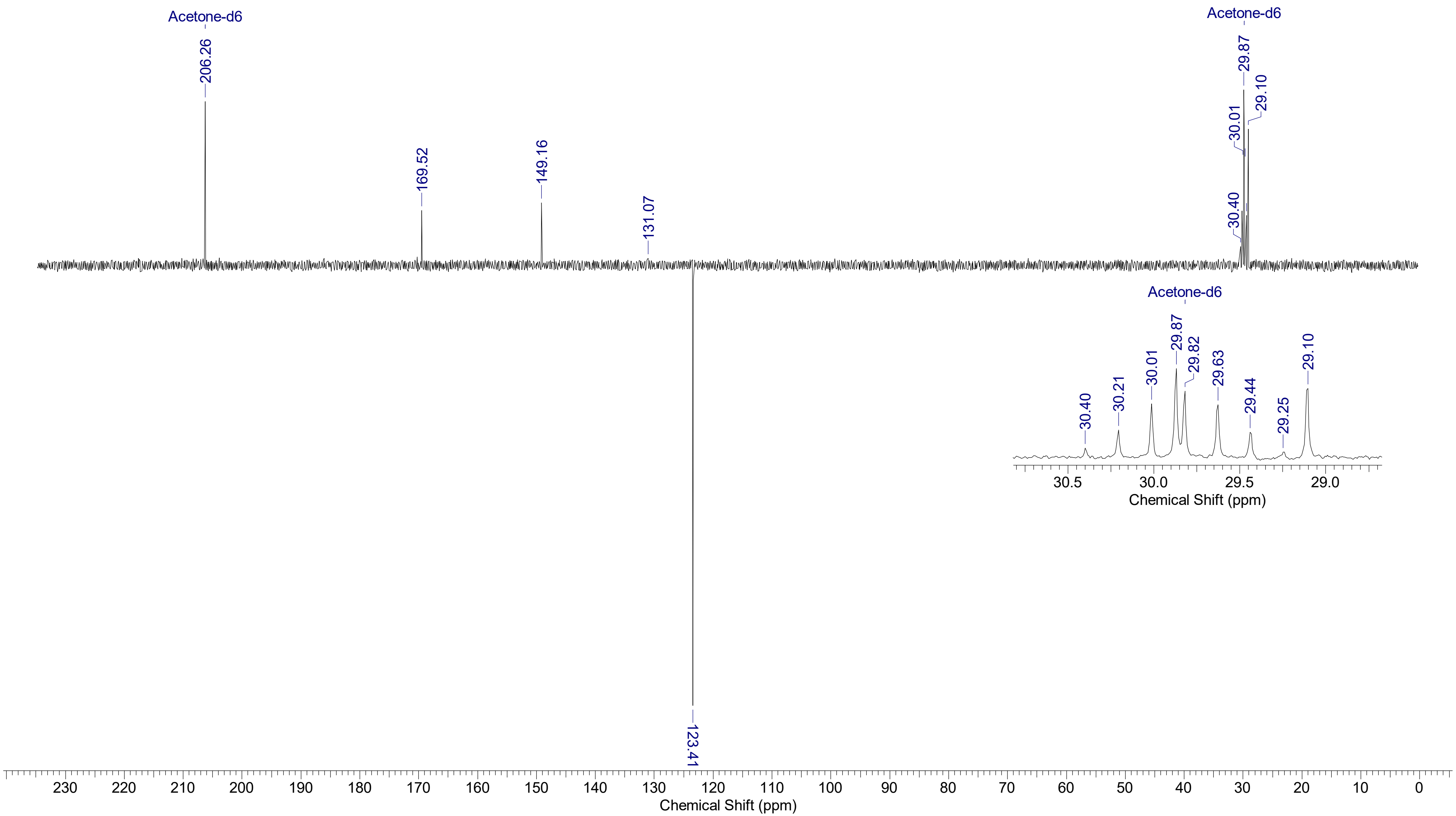

4-(Adamantan-1-yl)phenyl 4,4,4-trinitrobutanoate, **5b** (^{1}H NMR)

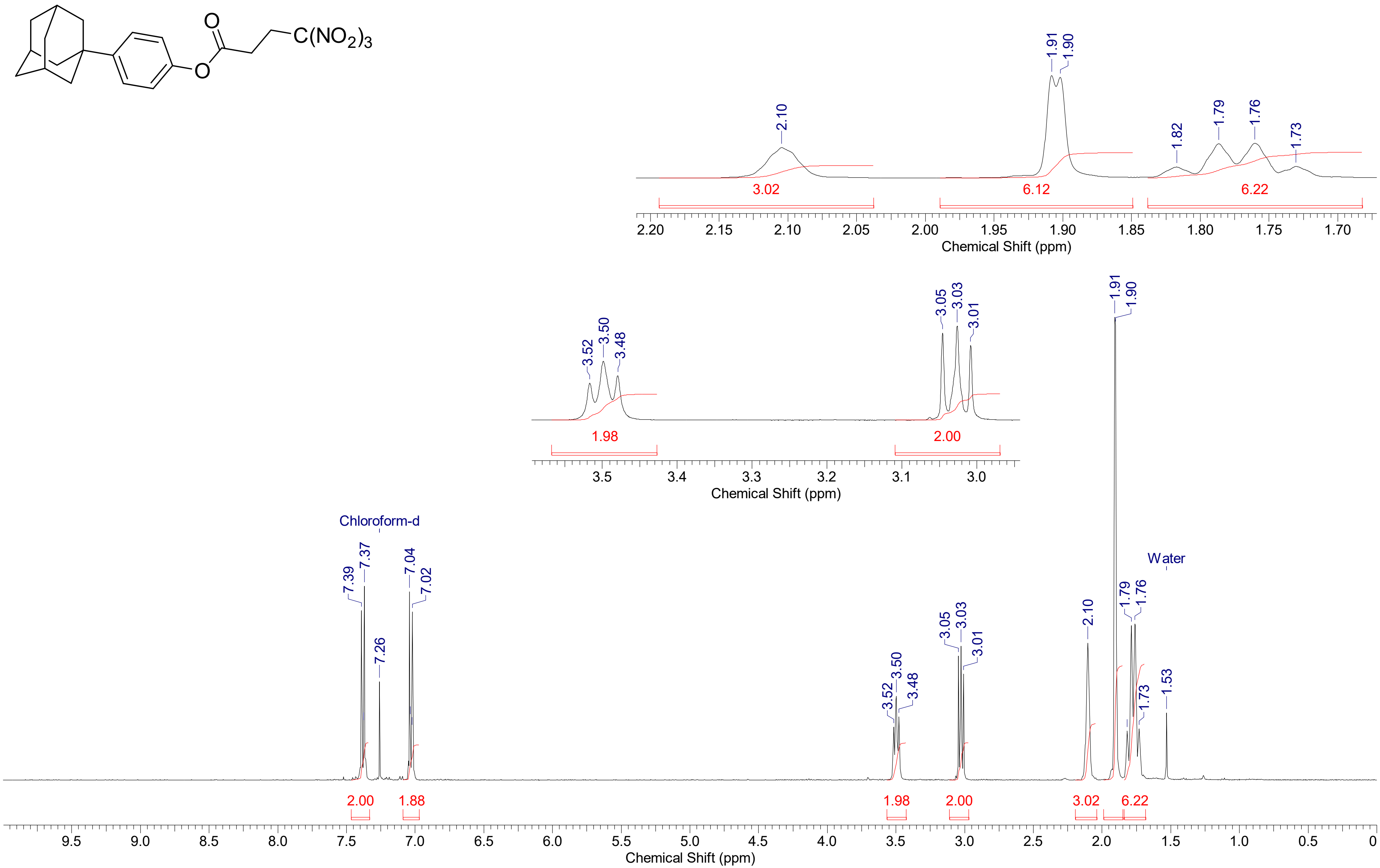

4-(Adamantan-1-yl)phenyl 4,4,4-trinitrobutanoate, **5b** (^{13}C NMR)

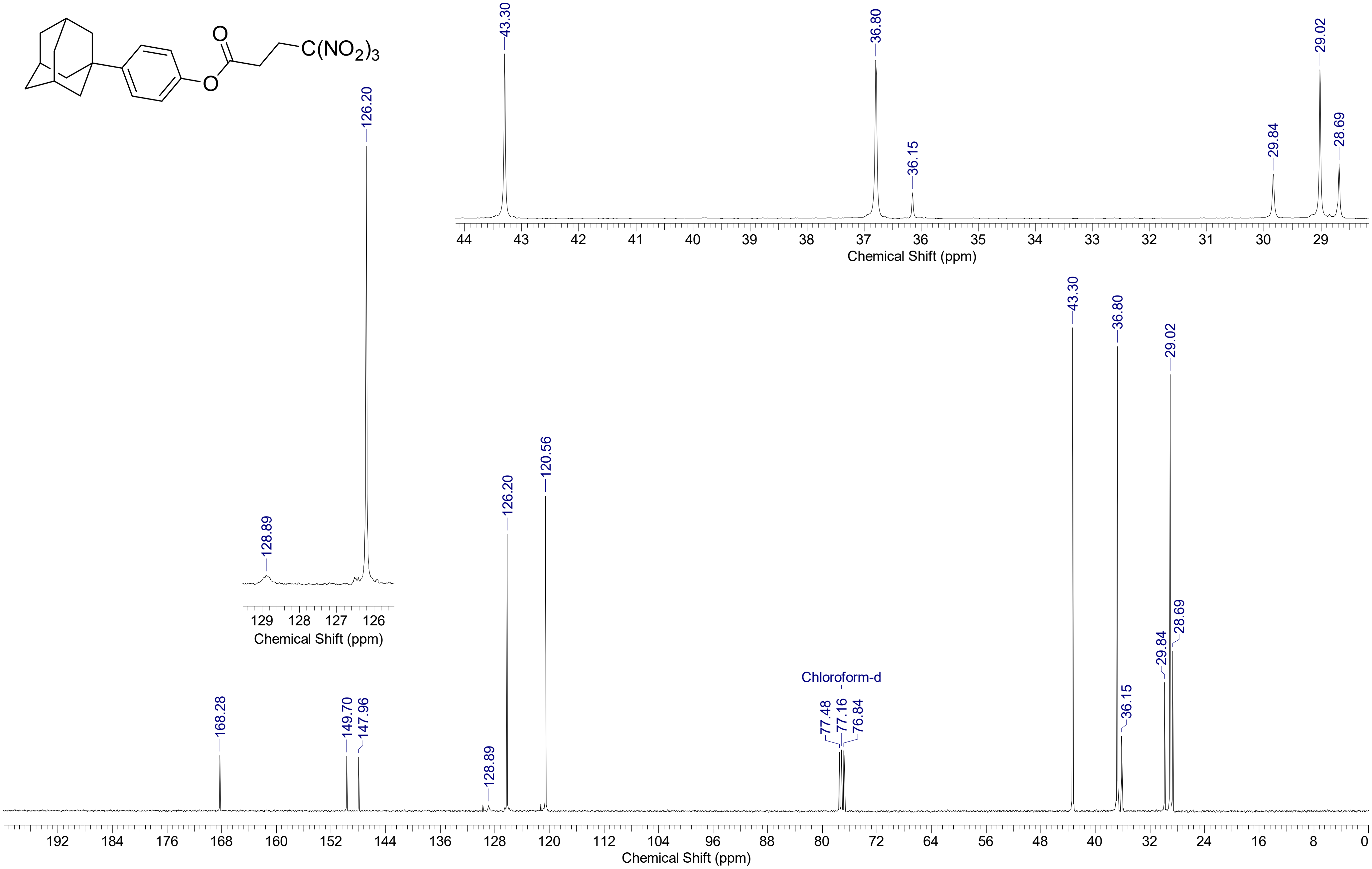

1,4-Phenylenedi(methylene) bis(5-aminoisoxazole-3-carboxylate) **6** (^{1}H NMR)

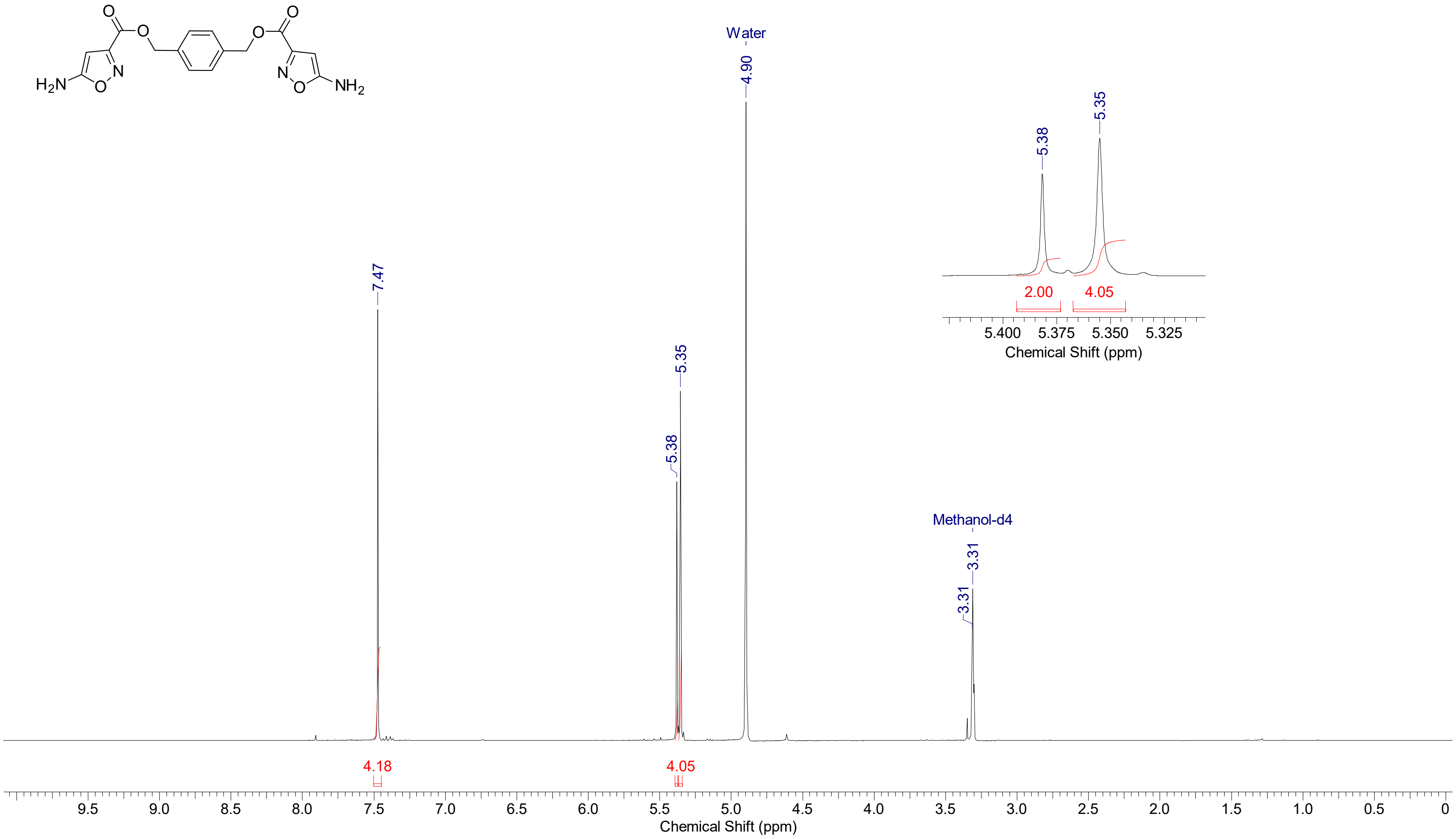

1,4-Phenylenedi(methylene) bis(5-aminoisoxazole-3-carboxylate) **6** (^{13}C NMR)

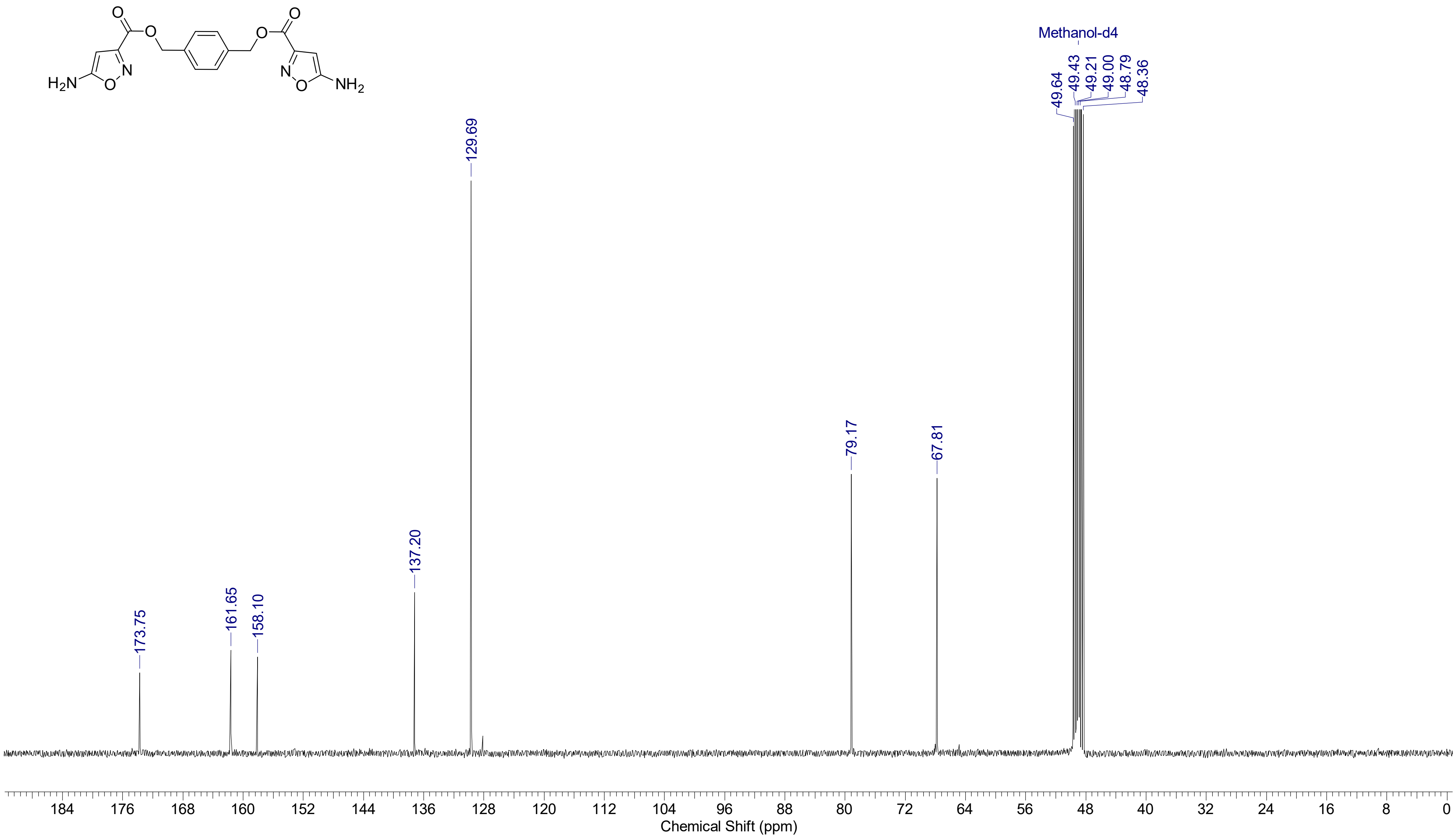

Butane-1,4-diyl bis(5-acetamidoisoxazole-3-carboxylate), **7** (^{1}H NMR)

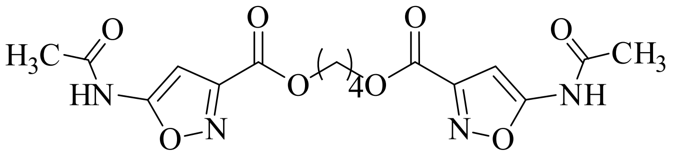

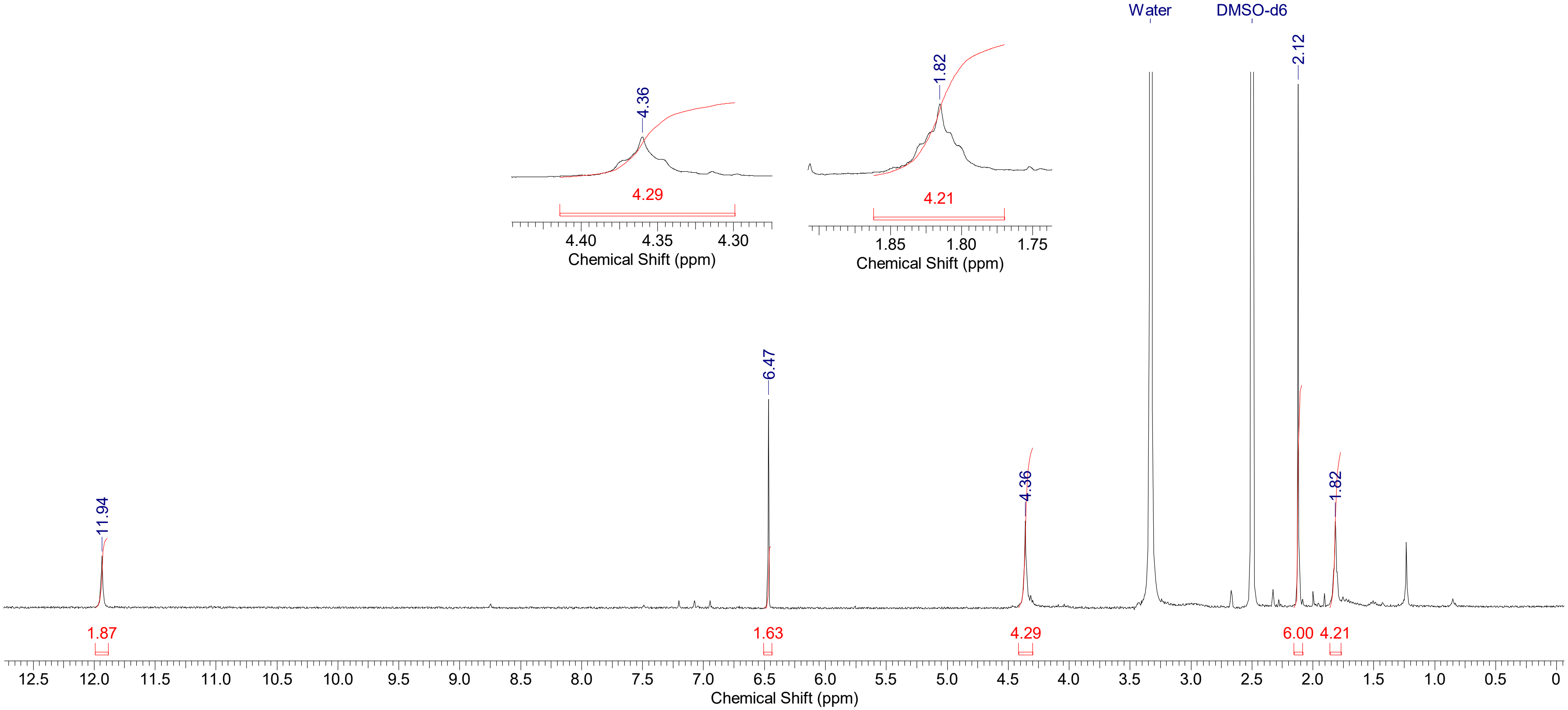

Butane-1,4-diyl bis(5-acetamidoisoxazole-3-carboxylate), **7** (^{13}C NMR)

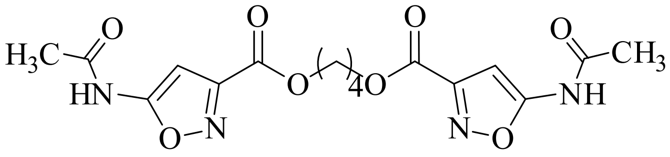

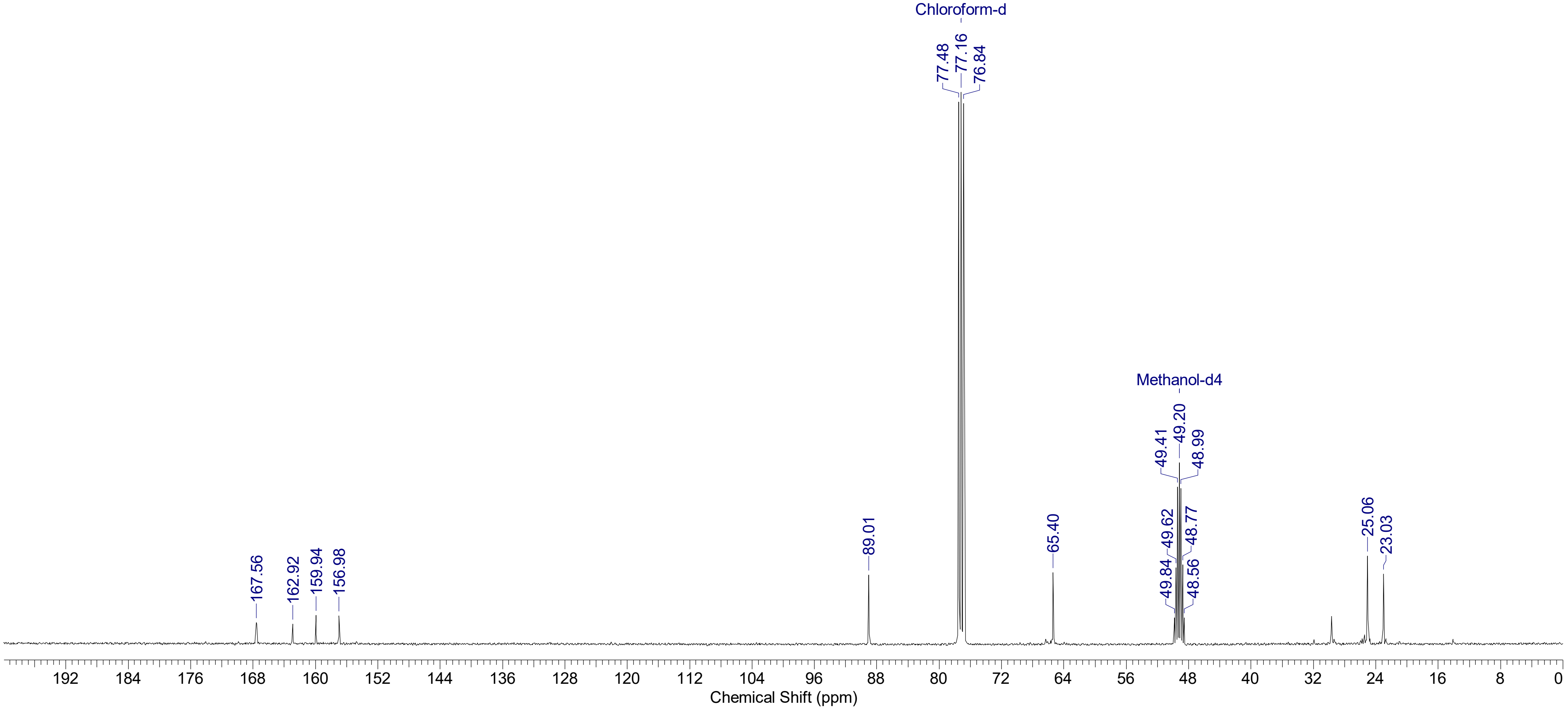

Butane-1,4-diyl bis(5-acetamidoisoxazole-3-carboxylate), **7** (HMBC)

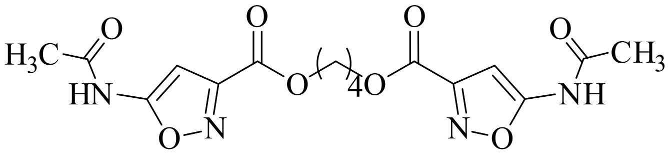

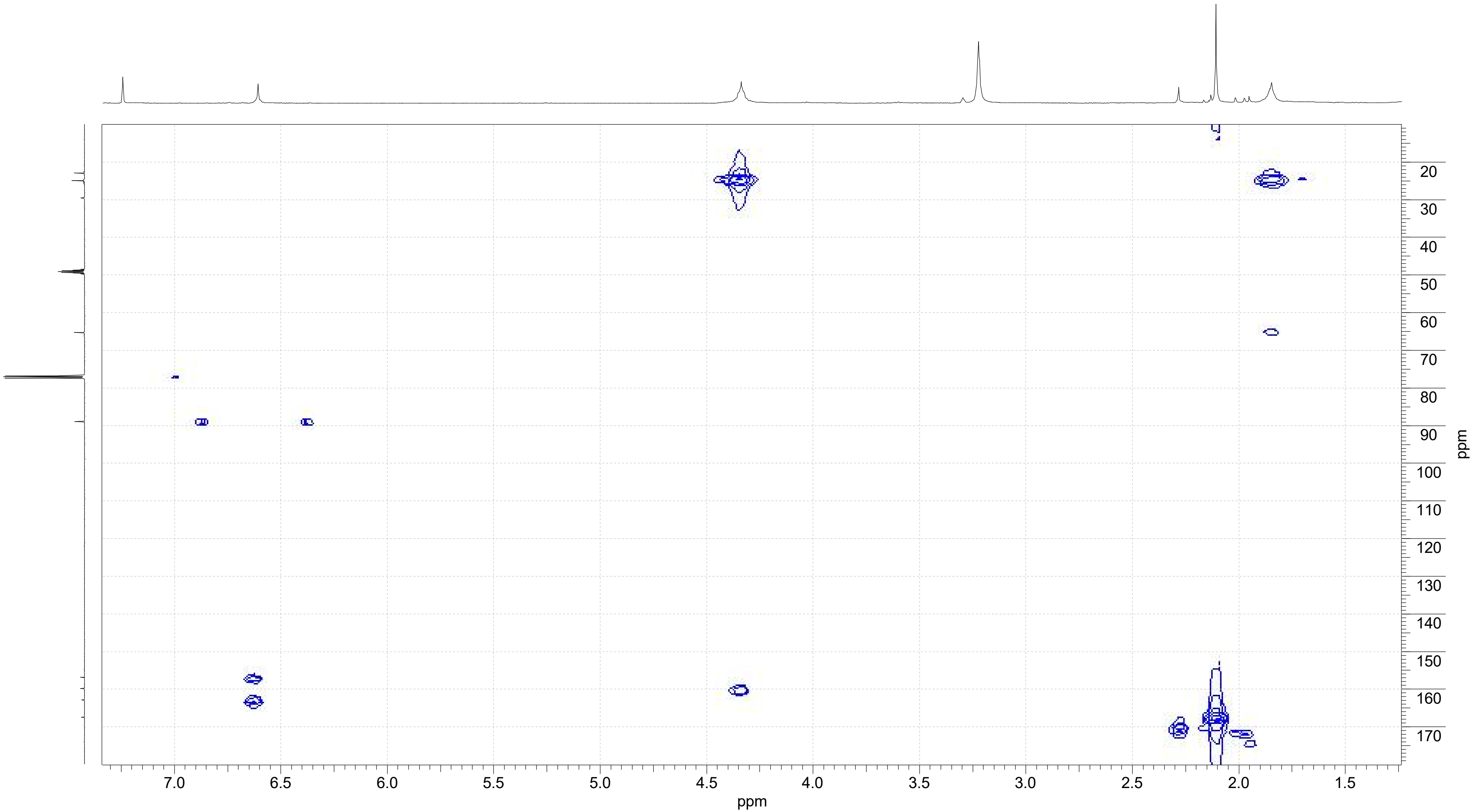

2. X-ray Diffraction Analysis

Single crystals suitable for X-ray diffraction were obtained by slow evaporation of the solvent from a chloroform solution of **3c**. Crystal data collection and refinement parameters of **3c** are summarized in Table S1. Intensity data were collected at 295 K on a Stoe STADI VARI diffractometer using focusing mirrors monochromated Cu Kα radiation, λ = 1.54186 Å. The data were corrected for decay, Lorentz, and polarization effects as well as absorption and beam corrections based on the multi-scan technique. The structures were solved by a combination of direct methods in *SHELX*S-97 and the difference Fourier technique, and refined by full-matrix least-squares procedures (*SHELXL-97*). Non hydrogen atoms were refined with anisotropic displacement parameters. The H-atoms were either located or calculated and subsequently treated with a riding model.

Table S1. Crystallographic data and structure refinement results of **3c**.

	**3c**
Empirical formula	$C_{12}H_{10}N_4O_{10}$
Formula weight	370.24
Temp, K	295(2)
Crystal system	monoclinic
Space group	$P2_1$
a, Å	9.000(2)
b, Å	9.1293(14)
c, Å	19.424(4)
α, (°)	90
β, (°)	92.197(18)
γ, (°)	90
Volume, $Å^3$	1594.8(5)
Z	4
d_{calc}, g·cm^{-3}	1.542
λ, Å	1.54186
μ, mm^{-1}	1.208
No. of data collected	4944
No. of unique data/ number restraints/ number refined parameters	4944/1/489
R_{int}	0.0217 (before absorption correction)
Goodness-of-fit on F^2	0.632
R_1, wR_2 (I > 2 (I))	0.0416, 0.0578
R_1, wR_2 (all data)	0.1244, 0.0736
